# NEK1 autophosphorylation is disrupted by amyotrophic lateral sclerosis-associated missense variants: activity biomarkers and structural insights

**DOI:** 10.1098/rsob.260209

**Published:** 2026-10-28

**Authors:** Shalini Agarwal, Syed Arif Abdul Rehman, Ivan M. Muñoz, Axel Knebel, Paul J. Hop, Robert Gourlay, Fiona Brown, Thomas Macartney, Iolo Squires, Jan H. Veldink, Kevin P. Kenna, John Rouse, Arpan R. Mehta

**Affiliations:** ^1^ MRC Protein Phosphorylation & Ubiquitylation Unit, Faculty of Life Sciences, University of Dundee, Dundee, UK; ^2^ Division of Molecular, Cell and Developmental Biology, Faculty of Life Sciences, University of Dundee, Dundee, UK; ^3^ Division of Genome Integrity, Faculty of Life Sciences, University of Dundee, Dundee, UK; ^4^ Protein Production and Assay Development, MRC Protein Phosphorylation & Ubiquitylation Unit, Faculty of Life Sciences, University of Dundee, Dundee, UK; ^5^ Mass Spectrometry Facility, MRC Protein Phosphorylation & Ubiquitylation Unit, Faculty of Life Sciences, University of Dundee, Dundee, UK; ^6^ MRC Reagents and Services, Faculty of Life Sciences, University of Dundee, Dundee, UK; ^7^ Department of Neurology, UMC Utrecht Brain Center, University Medical Center Utrecht, Utrecht University, Utrecht, The Netherlands; ^8^ Department of Translational Neuroscience, UMC Utrecht Brain Center, University Medical Center Utrecht, Utrecht, The Netherlands; ^9^ Department of Neurology, University College London Hospitals NHS Foundation Trust, Queen Square, London, UK; ^10^ Euan MacDonald Centre for MND Research, Scotland, UK; ^11^ Centre for Clinical Brain Sciences, School of Neurological and Cardiovascular Sciences, University of Edinburgh, Edinburgh, UK

**Keywords:** NEK1 kinase, autophosphorylation, amyotrophic lateral sclerosis, motor neuron disease

## Abstract

Rare variants in *NEK1*, encoding a serine/threonine kinase, are among the most consistently implicated genetic contributors to amyotrophic lateral sclerosis (ALS), reported in approximately 2–3% of cases. Yet, while recent studies have characterized the cell biological consequences of NEK1 loss-of-function, the biochemical effects of ALS-associated missense variants on kinase activity have not been directly investigated. This distinction is mechanistically important, because missense alleles encode mutant proteins rather than simply reducing protein dosage. Here, we provide the most comprehensive cell-based phosphoproteomic map of NEK1 phosphorylation to date, identifying 10 recurrent phosphorylation sites across independent expression and acquisition conditions. We experimentally assign pSer14, pThr156 and pSer418 as NEK1 autophosphorylation sites using kinase-dead controls, targeted extracted ion chromatogram analysis, phosphosite mutagenesis and phosphospecific antibodies. Leveraging activation-loop pThr156 as a read-out of NEK1 activity, we assessed nine ALS-associated missense variants spanning the major functional regions of the protein. Among catalytic-domain variants, R261C produced the most robust reduction in pThr156 autophosphorylation, R232C produced a smaller reduction, and R232H increased pThr156; the basic-region variant, A313T, also showed a smaller reduction. Structural modelling provides a mechanistic framework for understanding these variant-specific effects. Our study establishes the first activity-based framework for functional classification of *NEK1* missense variants, and provides direct evidence that ALS-associated missense variants can alter NEK1 autophosphorylation through a mechanism distinct from simple haploinsufficiency. The phosphospecific antibodies, isogenic cell lines and curated phosphoproteomic datasets generated here provide a community resource for future studies of NEK1 regulation, variant interpretation and therapeutic target validation.

## 1. Introduction

Amyotrophic lateral sclerosis (ALS) is a fatal neurodegenerative disease characterized by the progressive loss of upper and lower motor neurons [1]. While the majority of cases are apparently sporadic, a growing number of causative and risk genes have been identified through next-generation sequencing [2,3]. *NEK1* (Never In Mitosis A-related Kinase 1) encodes a serine/threonine kinase, and rare *NEK1* variants are now recognized as established contributors to ALS susceptibility worldwide, found in 2–3% of all cases [4–8]. Meta-analysis has confirmed that both nonsense and missense variants in *NEK1* are significantly associated with increased ALS risk [9].

Among ALS-associated *NEK1* missense variants, R261H has been associated with significantly increased disease susceptibility across multiple cohorts [4,10], an association now confirmed at exome-wide significance in the largest ALS sequencing study to date (OR = 2.01, *p* = 5.66 × 10^–13^). That study—a large-scale harmonized exome analysis of nearly 18 000 ALS cases and 200 000 controls across discovery and replication phases—confirmed NEK1 as one of only four established ALS genes that reached exome-wide significance in both single-variant and ultra-rare variant burden analyses in the discovery analysis (13 138 cases and 69 775 controls). Notably, the burden signal was enriched in singleton variants, consistent with strong purifying selection acting on functionally damaging *NEK1* missense alleles [11].

NEK1 is a pleiotropic kinase implicated in several critical cellular processes [12], and pathogenic variants have been linked not only to neurodegeneration, but also to a spectrum of skeletal ciliopathies and developmental dysplasias, including autosomal recessive short-rib polydactyly syndrome type Majewski [13,14], which lies within the short-rib thoracic dysplasia spectrum that also encompasses Jeune syndrome [15], and axial spondylo-metaphyseal dysplasia [16]. Cellular processes in which NEK1 has been implicated include the maintenance of genomic stability and DNA damage response [17,18], cell-cycle regulation [19,20], mitochondrial activity [21] and ciliogenesis [22–24]. Despite this breadth of cell biology, comparatively little is known about the molecular regulation of NEK1 itself, particularly its kinase regulation and substrate biology, and NEK1 has therefore been regarded as a ‘dark kinase’ [25], listed among the understudied kinases in the Dark Kinase Knowledgebase (https://darkkinome.org/kinase/NEK1).

NEK1 has a complex domain architecture comprising an N-terminal kinase domain, which contains the activation loop, followed by a basic region and a central regulatory region containing multiple coiled-coil domains (CC1–CC4) that serve as the primary protein-protein interaction interface [26,27]. The extreme C-terminus of NEK1 comprises an acidic C21ORF2-interaction domain (CID; residues 1160–1286 [28]) through which NEK1 forms a stable complex with C21ORF2 (also known as CFAP410), itself an ALS-associated ciliary and DNA-damage-response protein [29], with genetic evidence linking the NEK1–C21ORF2 axis to ALS risk [11]. This architecture provides several plausible mechanisms by which missense *NEK1* variants could alter NEK1 function, including direct catalytic impairment, altered autophosphorylation, disrupted intramolecular regulation or impaired protein–protein interactions.

Recent studies have begun to define the cellular consequences of NEK1 dysfunction, including disruption of microtubule homeostasis, nucleocytoplasmic transport and primary ciliogenesis [5,22,30]. However, these studies have largely focused on NEK1 depletion, nonsense alleles or kinase inhibition, leaving the biochemical consequences of ALS-associated missense variants comparatively unexplored. This gap is mechanistically important because heterozygous missense variants produce full-length mutant proteins and may cause disease through hypomorphic, dominant-negative or gain-of-function mechanisms rather than simple haploinsufficiency. Yet, no rigorously validated autophosphorylation sites or activity-based biomarkers currently exist for NEK1, leaving newly identified missense variants difficult to classify functionally. This mechanistic void is increasingly acute: large-scale sequencing studies continue to identify novel *NEK1* missense variants across the full domain architecture, including singleton variants that appear to carry particularly large effect sizes [11].

Here, we systematically define the autophosphorylation landscape of NEK1 and experimentally assign three NEK1 autophosphorylation sites, both within and outside its catalytic domain (pSer14, pThr156 and pSer418). These were identified by mass spectrometry, validated using kinase-dead controls and phosphosite mutagenesis and confirmed with phosphospecific antibodies. We then leveraged the activation-loop autophosphorylation (pThr156) as a read-out of NEK1 kinase activity to interrogate ALS-associated missense variants. The nine *NEK1* missense variants identified in ALS cases studied here span the full domain architecture of NEK1, from the kinase domain (V223M, R232C, R232H, R261C and R261H), to the basic region (A313T), to within or between the coiled-coil domains (R440Q, K648E and R721Q), but fall outside the CID, suggesting that any pathogenic effects are unlikely to arise from direct disruption of the mapped CID interface, and may instead involve altered catalytic regulation, autophosphorylation, conformation or protein–protein interactions. Using this activity-based approach, we show that ALS-associated variants exert distinct effects on NEK1 autophosphorylation. R232C, R261C and A313T reduced activation-loop phosphorylation, consistent with impaired kinase activation, whereas the recurrent variant R232H exhibited a modest increase in autophosphorylation, and several other variants had little detectable effect. Modelling of the catalytic-core substitutions suggests how these variant-specific differences arise. These findings establish the first activity-based biomarkers for NEK1 kinase function and provide a framework for functional interrogation of ALS-associated missense variants.

## 2. Results

### 2.1. A MANE Select-based domain framework defines ALS-associated *NEK1* missense variants for functional analysis

We first established a reference domain architecture of the NEK1 protein, anchored to isoform 3 (UniProtKB Q96PY6-3), derived from the **M**atched **A**nnotation from **N**CBI and **E**MBL-EBI (MANE) Select transcript (NM_001199397.3 / ENST00000507142.6; 34 coding exons), which encodes the longest NEK1 isoform at 1286 amino acids (figure 1A). Establishing isoform 3 as our reference framework is an important foundational step: isoform selection varies across the extant NEK1 literature, and consistent use of the MANE Select transcript ensures compatibility with current annotation standards, facilitating unambiguous cross-referencing of variant databases and published functional data.

**Figure 1.**
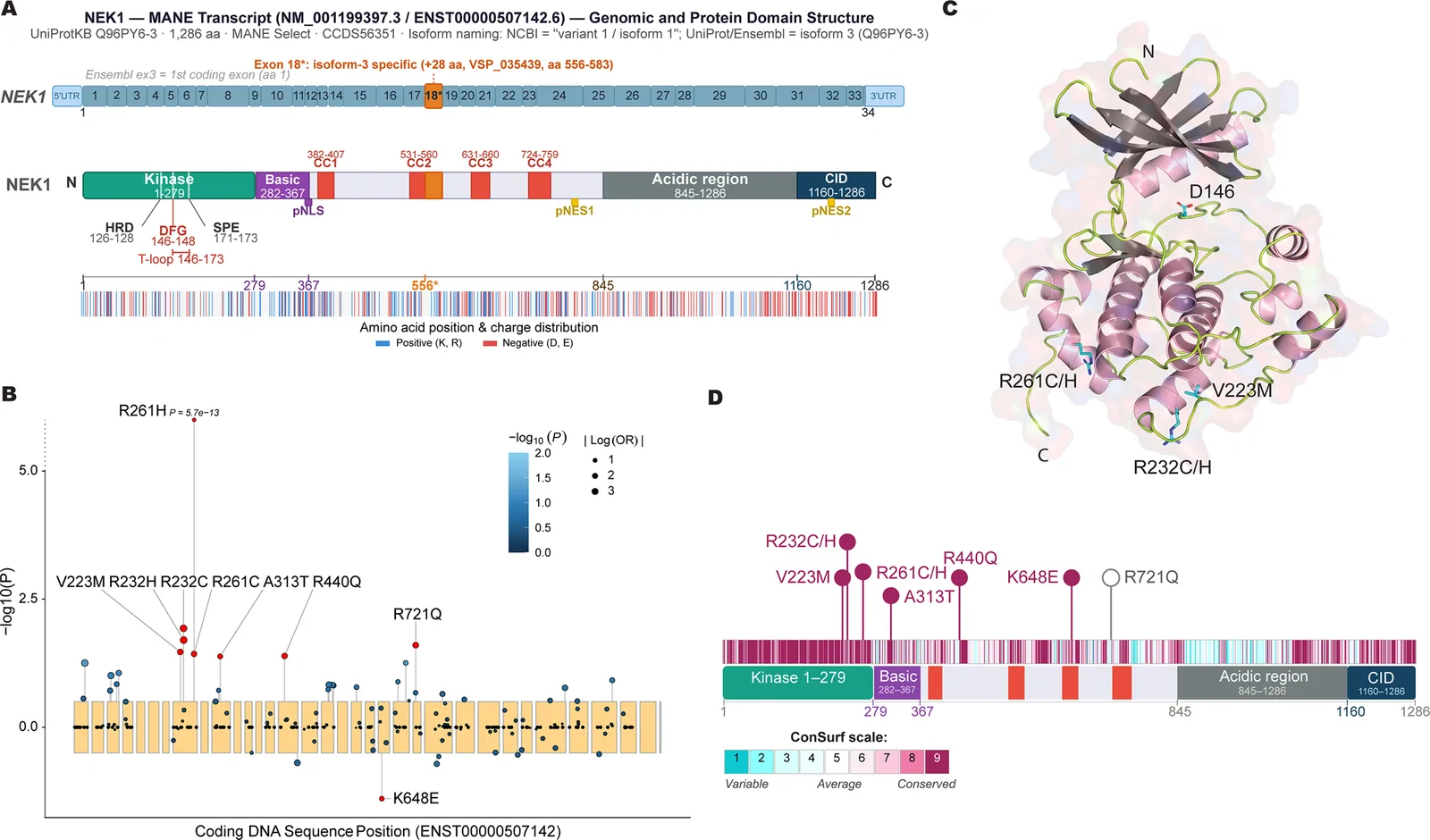
*NEK1* transcript structure, protein domain organization and prioritization of ALS-associated missense variants for functional characterization. (A) Schematic representation of the *NEK1* MANE Select transcript (NM_001199397.3 / ENST00000507142.6; UniProtKB Q96PY6-3), encoding a 1286 amino acid protein (CCDS56351; NCBI variant 1/isoform 1; UniProtKB/Ensembl isoform 3). Upper panel: exon organization of the *NEK1* gene, numbered 1–34 by coding exon (equivalent to Ensembl exons 3–36; exons 1–2 are non-coding 5′UTR), with the terminal coding exon (34) carrying only approximately four amino acids of coding sequence followed by approximately 1.7 kb 3′UTR and, therefore, appearing as a narrow terminal segment on the amino-acid-scaled axis. Exon 18* is isoform-3 specific, contributing an additional 28 amino acids encoded by alternatively spliced sequence VSP_035439 (amino acids 556–583). Middle panel: constructed linear map of NEK1 protein domains drawn approximately to scale from the N- to C-terminus. Annotated domains include the N-terminal kinase domain (aa 1–279), encompassing the HRD motif (aa 126–128), DFG motif (aa 146–148), SPE motif (aa 171–173) and T-loop (aa 146–173); a basic region containing a predicted nuclear localization signal (pNLS; aa 282–367); four coiled-coil domains (CC1–CC4; aa 382–407, 531–560, 631–660 and 724–759, respectively); a central acidic region (aa 845–1286); a C-terminal CID domain (aa 1160–1286); and two predicted nuclear export signals (pNES1, aa 794–803 and pNES2, aa 1210–1219), neither of which has been verified in human NEK1. Lower panel: amino acid charge distribution along full-length wild-type NEK1 (blue, basic (K, R); red, acidic (D, E)), plotted by position with key domain boundaries indicated. (B) Lollipop plot of single-variant ALS association statistics for rare *NEK1* variants annotated as moderate impact (missense and in-frame deletion) using SnpEff, plotted by coding DNA sequence position (ENST00000507142.6). The plot is restricted to variants passing the call-rate quality-control filters (see Methods) and with a minor allele frequency below 0.05. Each point represents one variant; point size reflects |log(OR)| and fill colour indicates –log10(*p*) on a scale truncated at 2, chosen so that variation among the majority of variants is resolvable. Variants exceeding the plotted y-axis range are shown above an axis break and annotated with their exact *p*-value. Variants drawn in red, in place of the -log10(*p*) colour scale, were nominally associated with ALS risk (*p* < 0.05); these nine variants are those selected for functional characterization in this study, and are labelled by amino acid substitution, with leader lines indicating the corresponding data points. Yellow shading demarcates individual exons. Note that K648E is oriented below the baseline, indicating a lower frequency in ALS cases than in matched controls (supplementary material, figure S1A). (C) AlphaFold3 structural model of the NEK1 kinase domain, with ALS-associated missense variants selected for functional characterization indicated at their respective positions (V223M, R232C/H and R261C/H). D146A, which renders NEK1 kinase-dead, is also indicated as a structural reference point (see supplementary material, figure S1B, for all variants mapped onto the full-length wild-type structure). (D) Lollipop schematic of ALS-associated *NEK1* missense variants selected for functional characterization, mapped onto the NEK1 protein domain structure (as in A). Circle colour reflects evolutionary conservation at each position, scored on the ConSurf scale (1–9; variable to conserved).

Structural examination of the NEK1 kinase domain crystal structure (PDB: 4APC / 4B9D [26]) placed the kinase domain boundary at residues 1–279, extending beyond the frequently cited boundary of residues 1–258 and reflecting a more complete delineation of the catalytic fold. The kinase domain harbours the conserved HRD (aa 126–128) and DFG (aa 146–148) motifs, with the activation loop spanning residues 146–173. NEK1 kinase activity is regulated by phosphorylation at multiple sites within this domain, including TLK1-dependent phosphorylation at Thr141 [31]. Structural analysis of the kinase domain, using a T162A substitution to trap an inactive activation-loop conformation, has further implicated activation-loop dynamics in autoactivation [26]. Beyond the kinase domain, we delineated a basic region harbouring a predicted nuclear localization signal (pNLS; aa 282–367), four coiled-coil domains (CC1–CC4), a large central acidic region and a C-terminal C21ORF2 interaction domain (CID; aa 1160–1286) (as detailed in Materials and methods, and summarized in figure 1A). We note that, although depicted as a discrete element in the schematic for clarity, the basic residues are distributed more broadly than the boundaries shown would suggest, as is also apparent in the Jalview alignment analysis.

To identify ALS-associated *NEK1* missense variants for functional characterization, we interrogated single-variant association statistics from the discovery cohort (13 138 ALS cases and 69 775 controls) of a large-scale exome-wide rare variant study [11]. Applying stringent call-rate quality control filters and a nominal significance threshold of *p* < 0.05, we identified nine ALS-associated *NEK1* missense variants: V223M, R232C, R232H, R261C, R261H, A313T, R440Q, K648E, and R721Q (figure 1B; see supplementary material, figure S1A and table S6, for association statistics across all *NEK1* rare variant classes, including nonsense, frameshift and splice donor/acceptor variants). All were nominally associated with increased ALS risk, except K648E, which was instead associated with decreased risk. Mapping these variants onto an AlphaFold3 structural model of full-length NEK1 revealed that several cluster within or in close proximity to the catalytic core (figure 1C; supplementary material, figure S1B). Assessment of evolutionary conservation using ConSurf scores derived from alignment of NEK1 orthologues across 26 vertebrate species demonstrated that eight of the nine variant positions are highly conserved (ConSurf grade 9), whereas the most C-terminal variant position, R721, showed only intermediate conservation (grade 5; figure 1D; supplementary material, figure S2). The high conservation of the catalytic- and central-region positions is consistent with their functional importance and supports their prioritization for experimental characterization.

### 2.2. Phosphoproteomic profiling identifies recurrent NEK1 phosphorylation sites in cells

To define the cellular phosphorylation landscape of NEK1, we used a GFP-NEK1 re-expression strategy in a genetically defined NEK1-knock-out U-2 OS background, comparing wild-type (WT) and kinase-dead (KD; D146A) NEK1. GFP-NEK1 was immunoprecipitated and phosphosites mapped by LC-MS/MS (supplementary material, tables S1 and S2); the workflow is detailed below.

To establish this knock-out background, we generated a *NEK1* biallelic knock-out Flp-In T-REx U-2 OS cell line by CRISPR-Cas9 nickase-mediated targeting of exon 3, the first coding exon shared by all *NEK1* protein-coding isoforms (supplementary material, figure S3A). A paired D10A nickase guide design was used to minimize off-target editing, with cleavage positioned in close proximity to the ATG start codon (supplementary material, figure S3B,C). Biallelic gene disruption was confirmed by amplicon-based next-generation sequencing (MiSeq), which revealed distinct frameshift-inducing indels on both alleles—a 5 bp insertion on one allele and an indel abolishing the start codon on the other (supplementary material, figure S3D)—and by immunoblotting, which confirmed complete loss of NEK1 protein (figure 2A).

**Figure 2.**
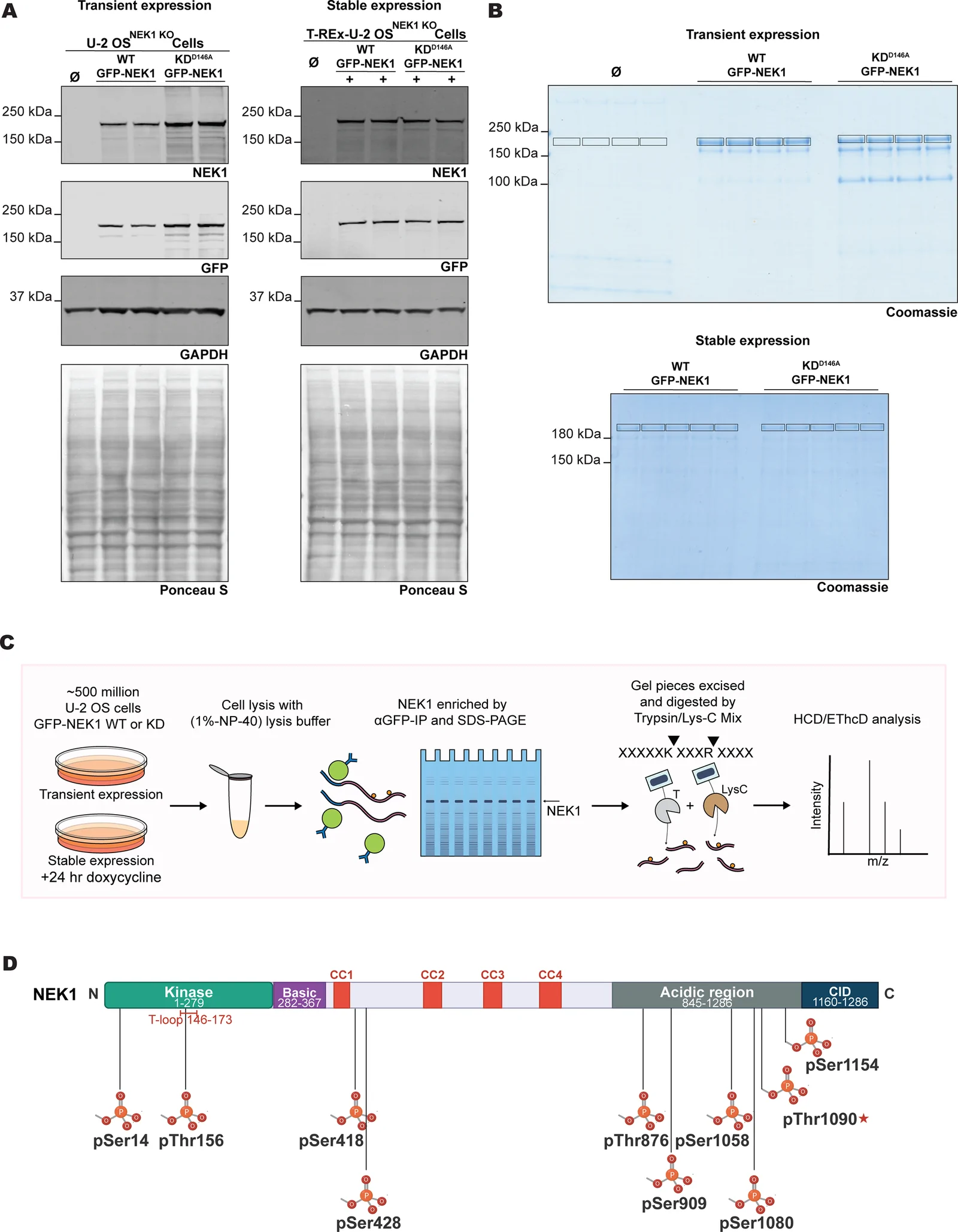
LC-MS/MS phosphosite mapping of immunoprecipitated GFP-NEK1 in cells. (A) Immunoblot validation of GFP-NEK1 expression in NEK1 knock-out U-2 OS cells. Left: transient overexpression of wild-type (WT) or kinase-dead (KD^D146A^) GFP-NEK1 or empty GFP vector (Ø). Right: stable doxycycline-inducible expression (0.1 µg ml^–1^) of WT or KD^D146A^ GFP-NEK1 in Flp-In T-REx NEK1 knock-out U-2 OS cells. Membranes were probed with antibodies against NEK1 and GFP; GAPDH and Ponceau S staining confirm comparable loading. (B) Colloidal blue-stained SDS-PAGE gels following anti-GFP immunoprecipitation from transiently (upper) or stably (lower) expressing cells, showing enrichment of GFP-NEK1 (WT and KD^D146A^) relative to empty vector control (Ø). Gel bands corresponding to NEK1 were excised for in-gel digestion and LC-MS/MS analysis. (C) Schematic of the phosphosite mapping workflow. GFP-NEK1 (WT or KD^D146A^) was expressed in NEK1 knock-out U-2 OS cells, enriched by anti-GFP immunoprecipitation, resolved by SDS-PAGE, excised and subjected to in-gel Trypsin/Lys-C digestion and LC-MS/MS using complementary HCD and EThcD fragmentation. (D) Lollipop schematic of the 10 NEK1 phosphorylation sites identified in wild-type NEK1 across all four cell-based acquisition conditions (pSer14, pThr156, pSer418, pSer428, pThr876, pSer909, pSer1058, pSer1080, pThr1090 and pSer1154), mapped onto the NEK1 protein domain structure (as in figure 1A). ★ Novel phosphosite not previously reported in PhosphoSitePlus. Wild-type versus kinase-dead detection across the cell-based datasets is summarized in supplementary material, figure S5A and table S2.

Into this NEK1 knock-out background, we introduced full-length GFP-NEK1 (WT or KD) via two complementary expression systems: transient overexpression and stable doxycycline-inducible expression using the Flp-In T-REx system (figure 2A). Stable lines were induced with a low dose of doxycycline (0.1 µg ml^–1^, 24 hours) selected to give modest expression rather than maximal induction, so that phosphosite mapping could be compared between near-endogenous stable expression and transient overexpression. Following anti-GFP immunoprecipitation, NEK1 was enriched and resolved by SDS-PAGE, and the NEK1 band was excised for in-gel Trypsin/Lys-C digestion and LC-MS/MS analysis (figure 2B,C). High sequence coverage was achieved across all samples; representative coverage maps for wild-type GFP-NEK1 are shown in supplementary material, figure S4. Phosphopeptides were identified using complementary higher-energy collisional dissociation (HCD) and electron-transfer/HCD (EThcD) fragmentation, the latter being particularly suited to unambiguous site localization on multiply phosphorylated peptides.

Across the four wild-type cell-based acquisition conditions—both transient overexpression and stable doxycycline-inducible expression, each analysed by HCD or EThcD—we detected 10 recurrent phosphorylation sites on wild-type NEK1: pSer14, pThr156, pSer418, pSer428, pThr876, pSer909, pSer1058, pSer1080, pThr1090 and pSer1154 (isoform 3 coordinates; figure 2D; supplementary material, table S1). Nine of these sites have been previously reported in PhosphoSitePlus [32], providing independent in-cell confirmation of their existence. The exception is pThr1090, located within the large central acidic region, which, to our knowledge, has not been reported previously and therefore represents a novel candidate phosphorylation site on NEK1.

In addition to these 10 recurrent sites, further phosphorylation events were detected only in subsets of the cellular datasets (supplementary material, table S1). Overall, our cell-based datasets detected 25 of the 55 phosphorylation sites currently annotated on NEK1 isoform 3 in PhosphoSitePlus (supplementary material, figure S5A, B), providing a broad in-cell phosphoproteomic map of NEK1. Several additional sites not currently listed in PhosphoSitePlus were also detected, including pSer295, pTyr565, pSer700, pSer761, pThr814, pThr1096 and pSer1252. Notably, pThr1090 was the only non-PhosphoSitePlus site detected across all four wild-type cellular acquisition conditions; the remaining non-annotated sites were detected in subset datasets only and are therefore treated as provisional pending independent confirmation.

Comparison of wild-type and kinase-dead GFP-NEK1 in the cell-based datasets provided an initial assessment of kinase-activity association (supplementary material, figure S5A and table S2). Among the 10 sites detected across all four wild-type cellular acquisition conditions, pThr156 and pThr1090 were not detected in kinase-dead cells, making them the clearest cell-based kinase-activity-associated candidates. By contrast, the remaining eight—pSer14, pSer418, pSer428, pThr876, pSer1058, pSer909, pSer1080 and pSer1154—were also detected in kinase-dead cells. Cell-based presence or absence therefore separated only pThr156 and pThr1090 from these eight, and could not rank the eight among themselves. Because discovery-mode LC-MS/MS detection is not quantitative, and absence from a dataset can reflect sampling rather than true absence, binary WT/KD detection alone could not reliably assess kinase-dependence. We therefore integrated the cell-based map with recombinant wild-type/kinase-dead NEK1 phosphosite mapping and targeted extracted ion chromatogram (XIC) analysis to quantify WT/KD enrichment across prioritized candidate sites. This workflow underpins our distinction throughout the study between recurrent phosphorylation sites, kinase-activity-associated candidate sites and autophosphorylation sites established by orthogonal biochemical validation.

### 2.3. Integrated phosphosite mapping, mutagenesis and antibody validation define pSer14, pThr156 and pSer418 as NEK1 autophosphorylation sites and activity read-outs

To refine this candidate set, we next mapped phosphorylation sites on recombinant wild-type and kinase-dead NEK1 that had been GFP-tagged for purification, co-expressed with C21ORF2 in insect cells to improve protein yield and stability, purified by anti-GFP affinity chromatography and recovered by RV-3C cleavage, leaving the GFP tag on the column, before SDS-PAGE and LC-MS/MS analysis. Recombinant wild-type and kinase-dead NEK1 were analysed across three independent biological replicates (supplementary material, figure S6 and table S3).

Applying a stringent 3/3 biological-replicate detection threshold, we identified 26 phosphorylation-site detections on wild-type recombinant NEK1, including one lower-confidence provisional site flagged in supplementary material, table S3, and seven sites on kinase-dead NEK1. Several recurrent cell-based sites were also detected in recombinant wild-type NEK1 and absent from recombinant kinase-dead—namely pThr156, pThr1090, pSer14, pSer418, pSer428, pThr876 and pSer1058, seven of the 10 recurrent cell-based sites. The three remaining recurrent sites—pSer909, pSer1080 and pSer1154—were not supported by this comparison and were not carried further. This recombinant WT/KD comparison therefore provided an independent filter for kinase-activity-associated candidate sites (figure 3A), including sites that remained detectable in kinase-dead cells and could not be resolved by cell-based presence/absence analysis alone.

**Figure 3.**
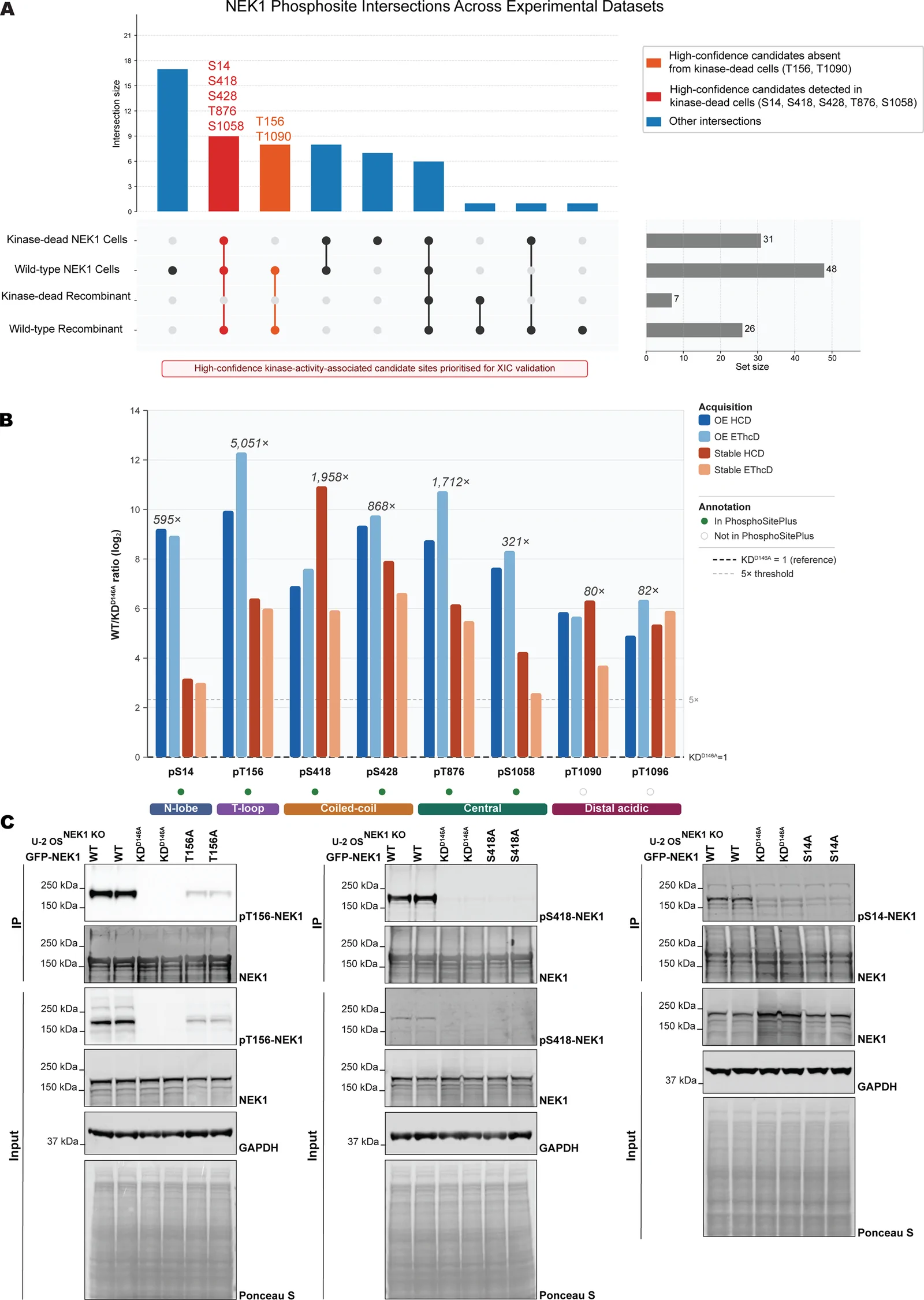
Integrated phosphosite mapping, mutagenesis and antibody validation define pSer14, pThr156 and pSer418 as NEK1 autophosphorylation sites and activity read-outs. (A) UpSet plot showing the intersection of NEK1 phosphorylation sites identified across recombinant protein and cell-based mass spectrometry datasets. Vertical bars indicate the number of phosphorylation sites shared by the dataset combination shown in the dot matrix below (filled circles, connected by a vertical line where two or more sets intersect; open grey circles indicate set non-membership). Horizontal bars (right panel) show the total number of sites detected in each individual dataset. Four experimental groups are represented: recombinant wild-type NEK1 (two fragmentation methods combined, 3/3 replicate threshold applied); recombinant kinase-dead NEK1 (two fragmentation methods combined, 3/3 replicate threshold); wild-type NEK1 expressed in cells (union of four datasets: stable and overexpression lines, HCD and EThcD fragmentation); and kinase-dead NEK1 expressed in cells (union of four datasets). Orange bars highlight high-confidence candidate kinase-activity-associated sites detected in recombinant WT and all four wild-type cellular datasets, but absent from kinase-dead cell datasets: T156 and T1090. Red bars highlight additional candidate sites that were also detected in kinase-dead cell datasets and therefore required XIC-based assessment: S14, S418, S428, T876 and S1058. The coloured bars indicate candidate-prioritization groups rather than final autophosphorylation-site assignment; experimental assignment of pSer14, pThr156 and pSer418 as NEK1 autophosphorylation sites was based on XIC analysis, kinase-dead controls, phosphosite mutagenesis and phosphospecific antibody validation. T1096 is annotated separately as a provisional candidate (detected in 3/4 cellular datasets; figure 3B). All positions refer to NEK1 isoform 3 (iso3) coordinates. (B) Targeted XIC quantification of WT/KD^D146A^ phosphopeptide enrichment across prioritized candidate NEK1 phosphosites. For each XIC-assessed candidate site, the wild-type to kinase-dead (WT/KD^D146A^) peak-area ratio is plotted on a log2 scale across all four cell-based acquisition conditions (overexpression HCD, overexpression EThcD, stable HCD and stable EThcD). Peak areas were normalized to total ion current within each run, and the KD^D146A^ signal was set to 1 (KD^D146A^ = 1; dashed line); the fivefold reporting threshold is also indicated. The value above each cluster gives the maximum WT/KD^D146A^ ratio across the four conditions. Coloured bars beneath the axis indicate the domain context of each site (N-lobe, T-loop, coiled-coil, central and distal-acidic regions). Filled green circles denote sites annotated in PhosphoSitePlus; open circles denote sites not currently annotated in PhosphoSitePlus. pThr1090 was detected across all four wild-type cellular acquisition conditions, whereas pThr1096 was detected in three of four cellular datasets and in recombinant wild-type NEK1. pThr1096 is included as a provisional kinase-activity-associated candidate (detected in three of four cellular datasets, absent from stable EThcD; localized to an independent singly-phosphorylated peptide, TLMDVPT[+80]VGDVR) and is reported alongside, but distinct from, pThr1090. Source data are provided in supplementary material, table S4. (C) Immunoblot validation of in-house generated phosphospecific sheep polyclonal antibodies against pThr156 (left), pSer418 (middle) and pSer14 (right) in NEK1 knock-out U-2 OS cells expressing the indicated GFP-NEK1 constructs, including wild-type, kinase-dead (KD^D146A^) and alanine substitution mutants at the relevant phosphosite (T156A, S418A and S14A). Phosphospecific antibodies detect signal in WT that is abolished or strongly reduced in KD^D146A^ and in the corresponding phosphosite mutant. For pThr156 and pSer418, both immunoprecipitate (IP) and input blots are shown; for pSer14, only the IP is shown, as the signal was below the limit of detection in whole-cell lysate input. Total NEK1, GAPDH and Ponceau S staining confirm comparable loading and immunoprecipitation efficiency.

The integrated comparison also highlighted pSer295 as an additional candidate outside existing PhosphoSitePlus annotation; however, unlike pThr1090, it was not detected across all four wild-type cellular acquisition conditions, and, unlike pThr1096, it was also detected in kinase-dead cells, so it was not carried forward for targeted WT/KD interrogation.

These analyses defined eight phosphosites for quantitative WT/KD interrogation by XIC analysis: the seven sites above plus pThr1096, which was detected in three of four cellular datasets and in recombinant wild-type NEK1. Representative spectra supporting localization of the additional XIC-assessed candidate sites are shown in supplementary material, figures S7 and S8. All eight sites showed at least one acquisition condition with greater than fivefold WT/KD enrichment, with maximum WT/KD ratios ranging from 80-fold for pThr1090 to 5051-fold for pThr156 (figure 3B; source data are provided in supplementary material, table S4).

From these candidates, we selected pSer14, pThr156 and pSer418 for in-depth validation because they combined recurrent detection across all four cellular acquisition conditions, independent support in recombinant wild-type NEK1, absence from recombinant kinase-dead, high localization confidence (supplementary material, figures S9 and S10), and distribution across distinct regulatory regions of NEK1: the kinase-domain N-lobe, activation loop and central regulatory region, respectively. XIC peak-area quantification confirmed that signal at all three sites was markedly reduced or absent in kinase-dead NEK1 relative to wild-type across expression systems and fragmentation methods (figure 3B). Mapping these sites onto an AlphaFold3 model of NEK1 confirmed that all three lie at the protein surface, each within or immediately adjacent to a flexible element—the glycine-rich loop, the activation loop and the predicted flexible region C-terminal to CC1, respectively. In the unphosphorylated model, the Ser14 side chain is orientated inwards, towards the body of the kinase N-lobe, whereas the Thr156 and Ser418 side chains are solvent-exposed, and none of the three residues makes a notable intramolecular contact. Modelling the phosphorylated states indicated that pThr156 and pSer418 remain solvent-exposed and similarly unengaged, whereas pSer14 is positioned to form an ionic interaction with the guanidinium group of Arg161 in the activation segment (supplementary material, figure S11A). Furthermore, assessment of evolutionary conservation using ConSurf demonstrated that all three sites are highly conserved across mammalian NEK1 orthologues, consistent with their functional importance. By contrast, conservation across the broader human NEK kinase family was more variable, suggesting that these sites may contribute to NEK1-specific regulatory mechanisms, rather than representing a general feature of NEK family kinases ([33]; supplementary material, figures S2 and S11B).

To enable direct detection of these three candidate sites in cells, we generated in-house phosphospecific sheep polyclonal antibodies against them. Dot blot analysis of affinity-purified antibodies confirmed concentration-dependent recognition of the cognate phosphopeptide, with little detectable signal against the non-phosphorylated counterpart for all three antibodies (supplementary material, figure S12). Immunoblot validation in NEK1 knock-out U-2 OS cells expressing wild-type GFP-NEK1, kinase-dead GFP-NEK1 or the corresponding phosphosite mutants S14A, T156A or S418A confirmed that all three antibodies detect signal in wild-type NEK1 that is abolished or strongly reduced in kinase-dead and in the corresponding alanine-substituted NEK1, demonstrating that detection depends on both NEK1 catalytic activity and the cognate residue (figure 3C).

Residual signal nonetheless remains in two settings. In the pThr156 T156A lanes, it is itself dependent on NEK1 catalytic activity, and so cannot be attributed to phosphorylation-independent background alone. In the pSer14 kinase-dead and S14A lanes, it is consistent with the pSer14 antiserum having been affinity-purified on the phosphopeptide alone, rather than tandem-depleted. We did not detect phosphorylation of Ser155 or Thr162 in the corresponding datasets, so neither accounts for the residual pThr156 signal. A plausible alternative is cross-reactivity with a sequence-similar phosphorylation site elsewhere in NEK1: Thr141 lies in a context resembling the immunogen (TKDG-pT-VQLG, against VLNS-pT-VELA), is detected in wild-type but not in kinase-dead cells, and does not correspond to the immunogen sequence, so antibodies recognizing it would not have been removed by the non-phosphopeptide depletion step used during purification. Thr141 is a previously reported NEK1 phosphorylation site [31]. This possibility remains to be tested directly. For pSer14, the signal was detectable after immunoprecipitation, but not in whole-cell lysate input, consistent with low abundance of this phosphorylation event and/or reduced sensitivity of the pSer14 antibody under these conditions.

For pThr156, the presence of an adjacent serine residue (Ser155) raised the possibility of phosphosite misassignment within the peptide VLNSTVELAR. To exclude this, we expressed alanine substitution mutants S155A, T156A and S155A/T156A in the NEK1 knock-out background and quantified phosphorylation by XIC (supplementary material, figure S13). Mascot site localization remained unambiguously assigned to Thr156 in both wild-type and S155A NEK1, whereas the signal was absent in T156A and the double mutant. Concordant localization by EThcD fragmentation provided further confirmation that Thr156, rather than Ser155, is the phosphorylated residue. Consistent with this assignment, the S155A substitution had no appreciable effect on phosphorylation at pSer14 or pSer418.

Exploratory analysis of the activation-loop mutants suggested interdependence between NEK1 autophosphorylation events. Although T156A alone did not substantially reduce pSer14, combined S155A/T156A substitutions caused a marked reduction in pSer14 phosphorylation. A comparable double-mutant effect was observed for pSer418: although T156A alone increased phosphorylation relative to wild-type NEK1, the S155A/T156A double mutant reduced pSer418 signal (supplementary material, figure S13). These observations suggest that the local integrity of the Ser155/Thr156 activation-loop region may influence distal NEK1 autophosphorylation events, a hypothesis that will require replicated quantitative analysis.

Co-immunoprecipitation further confirmed that C21ORF2 binding was retained across all phosphosite-mutant constructs, indicating that the substitutions do not perturb NEK1–C21ORF2 complex formation (supplementary material, figure S11C). Notably, the C21ORF2 mobility shift was abolished in kinase-dead NEK1, but retained in the S14A, T156A and S418A phosphosite-mutant constructs, indicating that this NEK1-dependent electrophoretic shift requires catalytic activity, but is not abolished by individual loss of pSer14, pThr156 or pSer418.

Collectively, comparative phosphoproteomics, quantitative XIC analysis, phosphosite mutagenesis and phosphospecific antibody validation establish pSer14, pThr156 and pSer418 as *bona fide* NEK1 autophosphorylation sites and cellular read-outs of NEK1 kinase activity.

### 2.4. ALS-associated *NEK1* missense variants differentially alter activation-loop autophosphorylation

Having established pThr156 as a robust read-out of NEK1 kinase activity, we prioritized this site for ALS-variant screening because it lies within the kinase-domain activation loop and is detected by a validated phosphospecific antibody. We next asked whether ALS-associated missense variants alter autophosphorylation at this position. We stably re-expressed all nine ALS-associated variants in the NEK1 knock-out background using the Flp-In T-REx system and assessed pThr156 levels by immunoprecipitation and immunoblot (figure 4A,B). As expected, kinase-dead NEK1 abolished pThr156 signal almost completely (mean 0.046 relative to wild-type, *p* = 0.0002).

**Figure 4.**
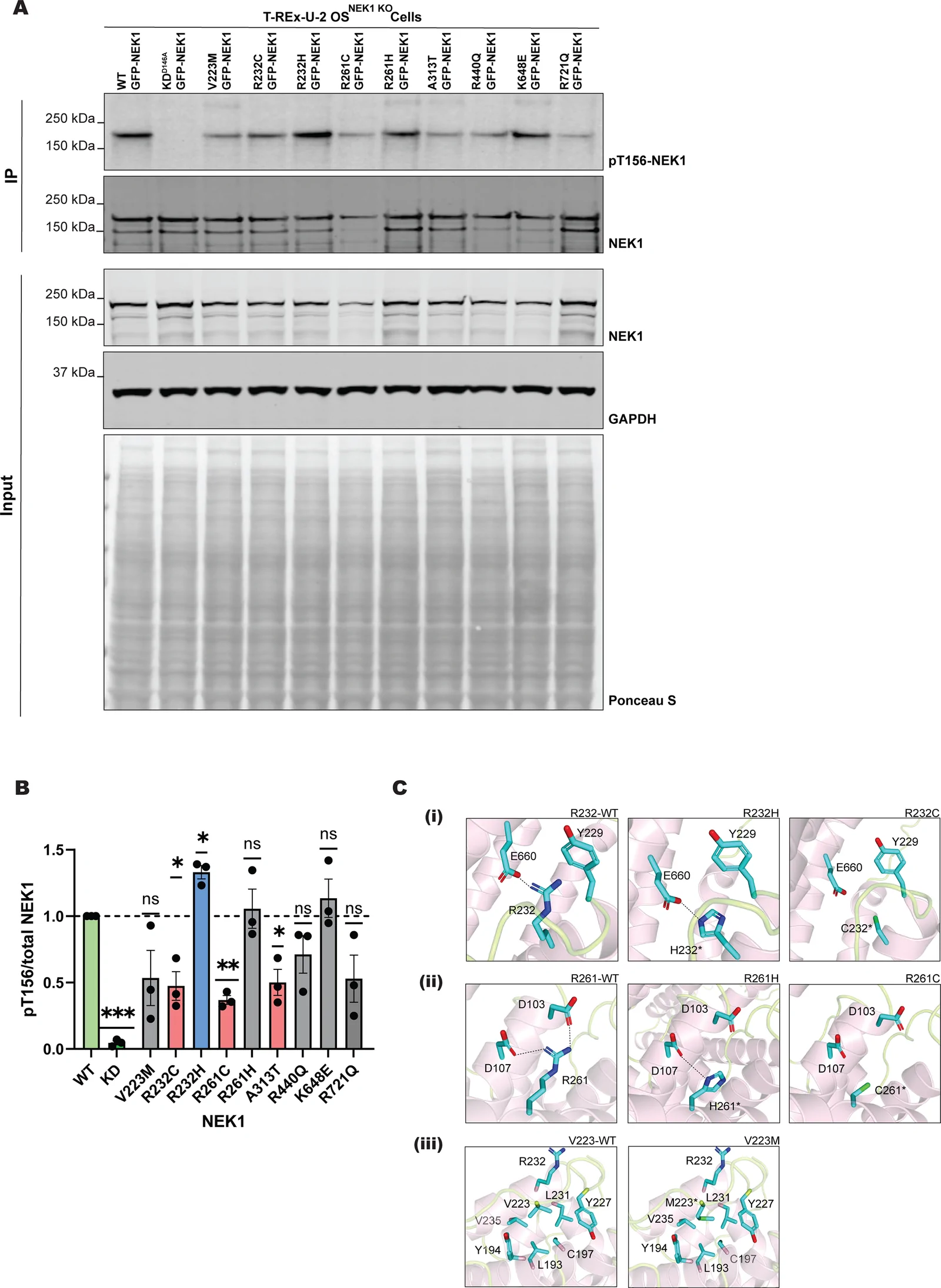
ALS-associated *NEK1* missense variants differentially alter activation-loop autophosphorylation at Thr156. (A) Immunoblot analysis of pThr156 in T-REx U-2 OS NEK1 knock-out cells stably expressing doxycycline-inducible (0.1 µg ml^–1^) GFP-tagged wild-type (WT), kinase-dead (KD^D146A^) or ALS-associated missense variant NEK1. IP fractions were probed with the in-house phosphospecific pThr156-NEK1 antibody, and total NEK1; input fractions were probed with total NEK1, GAPDH and Ponceau S to confirm comparable loading. (B) Quantification of pThr156 signal normalized to total NEK1 and expressed relative to wild-type. Data are presented as mean ± SEM from three independent experiments (*N* = 3). Each variant was compared with WT using a two-sided one-sample *t*-test against a hypothetical mean of 1.0. Constructs showing nominally significant differences from WT using unadjusted one-sample *t*-tests were: KD^D146A^ (***p = 0.0002), R232C (*p = 0.0395), R232H (*p = 0.0218; increased relative to WT), R261C (**p = 0.0027), and A313T (*p = 0.0364). V223M, R440Q and R721Q showed consistent reductions that did not reach statistical significance at *N* = 3; R261H and K648E did not differ significantly from wild-type. Multiple-comparison adjustment across the nine ALS-associated variants, with the kinase-dead control excluded as a positive control rather than a hypothesis under test, leaves R261C significant (Holm-adjusted *p* = 0.024; Benjamini–Hochberg *q* = 0.024), whereas R232H, A313T and R232C do not survive correction (Holm-adjusted *p* ≥ 0.17; *q* = 0.089 for all three). The rank order of effects is unchanged. *p < 0.05, **p < 0.01 and ***p < 0.001; *ns*, not significant. (C) AlphaFold3 structural models illustrating predicted intramolecular interactions disrupted by ALS-associated missense variants within the NEK1 catalytic core. Structural modelling was restricted to variants in the catalytic core. (i) R232 forms charge-based and cation-π interactions with E660 and Y229 in wild-type NEK1; R232H partially preserves these interactions, while R232C (asterisk; predicted strong disruptive effect) abolishes them. (ii) R261 engages in charge-based interactions with D103 and D107 in wild-type NEK1; R261H partially maintains these contacts, while R261C (asterisk; predicted strong disruptive effect) eliminates them. (iii) V223 contributes to a hydrophobic pocket involving R232, Y227, L231, V235, Y194, C197 and L193 in wild-type NEK1; V223M introduces methionine, disrupting the geometry of this pocket. Asterisks denote the mutant residue in each panel.

Of the nine ALS variants, four showed nominally significant differences from wild-type: three reductions in pThr156 and one increase. R261C produced the most severe reduction in pThr156 (mean 0.369, *p* = 0.0027), followed by R232C (mean 0.474, *p* = 0.0395) and A313T (mean 0.501, *p* = 0.0364). Conversely, R232H increased pThr156 relative to wild-type in the unadjusted analysis (mean 1.331; *p* = 0.0218). V223M, R440Q and R721Q showed lower mean pThr156 levels than wild-type, but these differences did not reach statistical significance at *N* = 3 and are therefore interpreted as trends. R261H, the variant with the strongest genetic support, did not significantly alter pThr156 under these assay conditions, indicating that ALS risk associated with this variant may involve mechanisms not captured by activation-loop autophosphorylation alone, or context-dependent effects not reproduced in this cellular system. The nominally protective variant K648E showed no reduction in activation-loop autophosphorylation.

To provide a structural framework for these observations, we generated AlphaFold3 models of full-length NEK1 bearing each variant, focusing on substitutions within the catalytic core where model confidence is highest (figure 4C). These models are a source of hypotheses, not definitive structural determinations. Substitutions at R232 elicited divergent effects on pThr156 autophosphorylation, with R232C reducing and R232H increasing the signal relative to wild-type. This opposing behaviour at a single residue argues against a simple uniform loss-of-function mechanism and suggests that the biochemical consequence of substitution at R232 is amino-acid-specific. The AlphaFold3 models suggest a structural basis for this difference: R232C is predicted to disrupt local charge-based and cation-π interactions, whereas R232H may partially preserve local interaction geometry, while introducing context-dependent histidine chemistry. The same divergence is predicted at R261, which engages D103 and D107 in wild-type NEK1: R261C is predicted to eliminate these charge-based interactions, whereas R261H partially maintains them. V223M is predicted to perturb a hydrophobic pocket within the catalytic core, consistent with its mild, non-significant reduction in pThr156, whereas A313T was not modelled because it lies in a disordered region not amenable to confident structural interpretation.

Taken together, these data show that ALS-associated *NEK1* missense variants can alter activation-loop autophosphorylation in a variant-specific manner. The largest reductions in pThr156 were observed for R261C, R232C and A313T; among these, R261C and R232C lie within the catalytic core and are predicted to disrupt conserved interaction networks. By contrast, R232H increased pThr156, indicating that ALS-associated *NEK1* missense variants do not act uniformly through simple loss of kinase activity.

## 3. Discussion

Here, we report the first comprehensively validated autophosphorylation sites on NEK1—pSer14, pThr156 and pSer418—and show that several ALS-associated missense variants reduce activation-loop autophosphorylation, providing direct biochemical evidence that missense variation can impair NEK1 kinase activity.

By integrating orthogonal fragmentation methods, cell-based and recombinant systems, and parallel wild-type versus kinase-dead comparative phosphoproteomics, we distinguished NEK1-catalytic-activity-dependent phosphorylation events from phosphorylation events that persist independently of NEK1 catalytic activity, including sites likely phosphorylated by other kinases. This distinction, often unresolved in kinase phosphoproteomics, strengthens the assignment of pSer14, pThr156 and pSer418 as NEK1 autophosphorylation sites. Because the recombinant protein was produced in Sf9 insect cells with C21ORF2 as the only co-expressed partner, no human NEK1-dependent kinase cascade was available in that setting; detection of the same three sites on tag-free recombinant wild-type NEK1, and their absence from the kinase-dead protein, therefore argues against their being installed by a downstream human kinase.

Previous work by Singh *et al.* identified pSer14, pThr156 and pSer418 (reported there as S417) in recombinant NEK1 and demonstrated that TLK1-mediated phosphorylation of Thr141 can stimulate NEK1 activity [31]. However, that study lacked kinase-dead controls and phosphospecific antibody validation, and the functional significance of these sites was not investigated. Our findings extend these observations by providing independent cellular evidence for pThr141 in the overexpression datasets (ptmRS: 100%; supplementary material, table S1) and, importantly, by establishing these sites as validated NEK1 autophosphorylation sites through the use of kinase-dead controls and phosphospecific antibodies.

Among the validated sites, pThr156 is of particular interest because it resides within the activation loop of the NEK1 kinase domain. Activation-loop phosphorylation typically stabilizes the active conformation by repositioning the DFG motif and organizing the catalytic machinery for substrate engagement [34]. The available NEK1 kinase domain structure was solved using a T162A substitution to stabilize an inactive activation-loop conformation during crystallization [26]. The structure provides a framework in which phosphorylation of the nearby Thr156 residue could promote transition from the inactive α-helical activation-loop conformation to an active kinase state. We note that Thr162, rather than Thr156, aligns with the activating Thr197 of protein kinase A, and that the same structure led to the proposal that Thr162 is positioned for autophosphorylation either in *cis* or within a domain-swapped dimer [26].

Our data argue against phosphorylation at Thr162. The tryptic peptide bearing it was identified in every wild-type cell-based acquisition and never in a phosphorylated form, and removing the competing acceptors in T156A and S155A/T156A cells did not unmask it. Thr162 is also absent from the NEK1 sites annotated in PhosphoSitePlus [32], whereas Ser155 and Thr156 are both annotated. Set against this is a single doubly phosphorylated missed-cleavage spectrum that could not be localized between Ser155, Thr156, Thr162 and Thr166. We therefore regard phosphorylation at Thr162 as unlikely in this system, while acknowledging that mass spectrometry cannot establish the absence of a low-occupancy site, and that we cannot exclude phosphorylation of Thr162 by a distinct kinase in another cell type or under conditions not sampled here. Thr156 remains the residue supported by localization-confident spectra and by loss of antibody signal in T156A cells. These considerations are not necessarily in conflict: the crystal structure was determined on an isolated kinase domain overexpressed in bacteria, where concentration-driven autophosphorylation need not reflect the physiological reaction [35], and the T162A substitution was introduced there to help the protein crystallize, not to test whether Thr162 is phosphorylated.

The Ser155/Thr156 activation-loop region also appears to influence distal NEK1 autophosphorylation. Mutation of either residue alone had limited effects on pSer14, whereas combined S155A/T156A substitution markedly reduced pSer14 and altered pSer418. These exploratory data raise the possibility that activation-loop integrity contributes to the broader organization of NEK1 autophosphorylation, although this will require replicated quantitative analysis to establish.

Beyond pThr156, the other validated sites point to regulatory regions outside the canonical activation-loop phosphorylation site. Ser14 lies within the kinase-domain N-lobe, where phosphorylation may contribute to NEK1-specific regulatory mechanisms. In AlphaFold3 models of the phosphorylated protein, pSer14 is predicted to engage Arg161 within the activation segment (supplementary material, figure S11A). If correct, this would offer a structural basis for the coupling between pSer14 and the Ser155/Thr156 region seen in our mutagenesis (supplementary material, figure S13B), and would render pSer14 less accessible in the folded protein than pThr156 or pSer418. Ser14 also occupies the position in the glycine-rich loop corresponding to Thr14 of CDK1 and CDK2, where phosphorylation is inhibitory [36,37]. If pSer14 acts similarly in NEK1, an S14A substitution would be predicted to increase kinase activity, a prediction directly testable with the isogenic S14A line and pThr156 read-out reported here. We note, however, that pSer14 is detectable only after immunoprecipitation, whereas pThr156 and pSer418 are detectable in input (figure 3C). This is consistent with lower occupancy at Ser14, which would limit the magnitude of any such effect, but equally with reduced sensitivity of the pSer14 antibody, particularly if the epitope is occluded by the predicted pSer14–Arg161 interaction (supplementary material, figure S11A); the two cannot presently be distinguished.

Ser418 lies outside the kinase domain, within the linker between CC1 and CC2 rather than within a coiled coil itself, downstream of the predicted basic/NLS-containing region and within the central region that mediates NEK1′s protein–protein interactions. Given that NEK1 has been reported to interact with importin-*β* (KPNB1) in human motor neurons, and that NEK1 loss-of-function disrupts nuclear import [5], phosphorylation at this site may influence NEK1 localization or its importin-associated interactions.

pThr1090, located within the poorly characterized central acidic region, is the most robust novel site in our dataset, being detected across all four cellular acquisition conditions with high localization confidence. Although it remains an activity-associated candidate rather than a validated autophosphorylation site, it highlights how much of the NEK1 phosphoproteome remains uncharacterized.

None of the three validated sites conforms fully to the linear substrate motif reported for NEK1 [38], which favours threonine with arginine at −1 and +2 and a bulky hydrophobic residue at −3: two of the three are serines, and only Thr156 carries the preferred hydrophobic residue at −3. This is not unexpected. The reported motif derives from positional-scanning peptide libraries, describing the preference of the isolated catalytic domain for a freely diffusible substrate presented in *trans*. Activation-loop autophosphorylation sites commonly, though not invariably, lie outside their own kinase's consensus, and are thought to be phosphorylated from a distinct, prone-to-autophosphorylate conformation [39]. Thr1090 is the exception, carrying arginine at −1 and leucine at −3, which would be consistent with direct phosphorylation by NEK1. The provisional candidate Thr1096 makes the opposite point: in the peptide TLMDVPTVGDVR it carries proline at −1, aspartate at −3 and glycine at +2, none of them a preferred residue, yet it was detected in three of four cellular acquisition conditions and in recombinant wild-type NEK1. Thr162 illustrates the limits of this reasoning: it matches the reported motif as closely as Thr1090, yet we detect no phosphorylation there, whereas Thr156—which carries a disfavoured glutamate at +2—is robustly phosphorylated. Motif conformity thus identifies candidate positions, but does not robustly predict which of them are used in the folded protein. The sites we detect, therefore, appear to fall into at least two classes: consensus-conforming positions of the kind the isolated catalytic domain prefers in a freely diffusible substrate, and geometrically constrained autophosphorylation events whose use is dictated by their position in the folded protein, rather than by local sequence.

The pThr156 assay revealed that ALS-associated *NEK1* missense variants do not behave as a single functional class. R261C robustly reduced activation-loop autophosphorylation, while R232C and A313T showed smaller reductions under the conditions tested. R232H increased pThr156, whereas R261H, K648E and the more distal variants did not significantly reduce this read-out under the conditions tested. These data support the broader genetic inference that at least some *NEK1* missense alleles are functionally damaging, but they also show that missense variation cannot be interpreted simply as uniform loss of kinase activity [11].

Structural modelling provides a plausible basis for the strongest effects. R232C and R261C are predicted to disrupt charge-based interaction networks within the catalytic core, consistent with their reduction of pThr156, whereas the corresponding histidine substitutions partially preserve these interaction networks and show different functional behaviour. Because arginine-to-histidine substitutions can introduce pH-sensitive behaviour through histidine protonation [40], it will be important to test whether R232H or R261H confer context-dependent effects on NEK1 activation-loop dynamics, particularly in ALS-relevant stress states. A related possibility that we have not tested is that the cysteines introduced by R232C and R261C are themselves subject to oxidative modification: a free thiol at this partially buried position could be oxidized in a manner dependent on the local redox environment, which is of particular interest given the oxidative stress associated with ALS [41]. Comparison with R232A and R261A, which remove the arginine side chain without introducing a reactive thiol, would distinguish this from loss of the charge network alone. The increase in pThr156 observed for R232H is especially informative because it shows that elevated activation-loop phosphorylation should not be equated with increased catalytic output. Rather, pThr156 reports one regulatory state of NEK1. Independently, the NEK1-dependent C21ORF2 electrophoretic mobility shift persists in the T156A mutant (supplementary material, figure S11C), consistent with the broader principle that activation-loop phosphorylation is not obligatory for all outputs of kinase activity [39].

This distinction is important for variant interpretation. Variants outside the catalytic core, including R440Q, K648E and R721Q, may have effects not captured by pThr156 alone, particularly through altered protein-protein interactions mediated by the coiled-coil or acidic regions. Consistent with this, R721 was the least conserved of the nine variant positions: maximum-likelihood ancestral-state reconstruction across the 26-species phylogeny inferred glutamine as the ancestral residue at the amniote, mammalian and primate ancestral nodes (PastML MAP/F81 posterior probability 0.88, 0.82 and 0.75, respectively), with arginine inferred at the catarrhine ancestor (posterior 0.98; supplementary material, figure S14). The human R721Q substitution is therefore consistent with reversion towards the inferred ancestral glutamine state, although the functional consequence of this change remains to be determined. Conversely, the lack of pThr156 reduction for the nominally protective K648E variant is consistent with the absence of a loss-of-activation phenotype in this assay, but does not establish a protective biochemical mechanism. Future functional classification of *NEK1* variants will therefore require a panel of read-outs, including autophosphorylation, substrate phosphorylation, localization and complex assembly.

The present study has several limitations. All experiments were performed in U-2 OS cells, and whether the same autophosphorylation hierarchy operates in cortical neurons and/or motor neurons remains to be established. The pThr156 read-out captures only one dimension of NEK1 kinase regulation. Variants were also expressed in a homozygous knock-out background with re-expression of a single allele, which does not fully model the heterozygous state of most ALS patients or potential dominant interactions between mutant and wild-type NEK1. *In vitro* kinase assays on dephosphorylated recombinant NEK1, and mixing experiments with distinguishable active and kinase-dead protein, have not been performed; these are the priority next experiments for discriminating *cis* from *trans* autophosphorylation. Comparing full-length NEK1 with the isolated catalytic domain, with and without C21ORF2, would further establish whether the catalytic domain can autophosphorylate independently and whether C21ORF2 promotes the reaction. Finally, we have not measured site occupancy; thus, we cannot say whether the three sites are phosphorylated on the same NEK1 molecule or distributed across different molecules—a question addressable by sequential immunoprecipitation with the phosphospecific antibodies, or by targeted parallel reaction monitoring with heavy-labelled standards.

Notwithstanding these limitations, the phosphospecific antibodies, isogenic cell lines, curated phosphoproteomic datasets and variant panel generated here constitute a resource of immediate utility to the NEK1 and ALS research communities. In particular, the pThr156 assay provides a direct, antibody-based read-out of NEK1 activation-loop autophosphorylation that can now be deployed to classify newly identified missense variants, interrogate upstream regulators of NEK1 activation, and evaluate pharmacodynamic responses in disease-relevant cellular models including human induced pluripotent stem cell-derived neurons and glia. The relevance to therapeutic progress is direct: robust target validation and patient stratification by kinase activity are prerequisites for any future therapeutic development, reflecting the broader need for mechanism-informed approaches in neurodegeneration [42].

### 3.1. Opening up

This study establishes pSer14, pThr156 and pSer418 as validated autophosphorylation-based read-outs of NEK1 kinase activity, but it also suggests that NEK1 regulation is unlikely to be governed by a simple single-site activation switch. The mutagenesis data raise the possibility of a hierarchical but nonlinear phosphorylation architecture, in which integrity of the Ser155/Thr156 activation-loop region influences distal autophosphorylation events. Future work should define how these sites are ordered within the NEK1 activation cycle, whether they mark distinct conformational or localization states, and how the additional kinase-activity-associated phosphosites fit into the broader sequence of NEK1 activation.

A second major open question concerns the architecture and regulation of the NEK1–C21ORF2 complex. Our size-exclusion chromatography and mass photometry analyses suggest that co-expression with C21ORF2 promotes formation of a higher-order assembly compatible with a putative 2:4 NEK1–C21ORF2 complex (supplementary material, figures S15 and S16). We therefore modelled a putative NEK1 dimer bound to a C21ORF2 tetramer using AlphaFold3 as a hypothesis-generating framework, rather than an experimentally determined structure or stoichiometry (figure 5A). Electrostatic surface analysis using APBS revealed marked charge complementarity at the predicted interface, with the acidic C-terminal region of NEK1 opposing a basic N-terminal surface on C21ORF2 (figure 5B,C). Defining the precise stoichiometry and architecture of this complex will be important for understanding whether NEK1 autophosphorylates in *cis*, in *trans* or within a higher-order signalling assembly, and whether ALS-associated variants in *NEK1* or *C21ORF2* alter complex formation or signalling output. Experimental structure determination of the complex, for example by cryo-electron microscopy, is the definitive route to resolving both the stoichiometry and the relative orientation of the two kinase domains.

**Figure 5.**
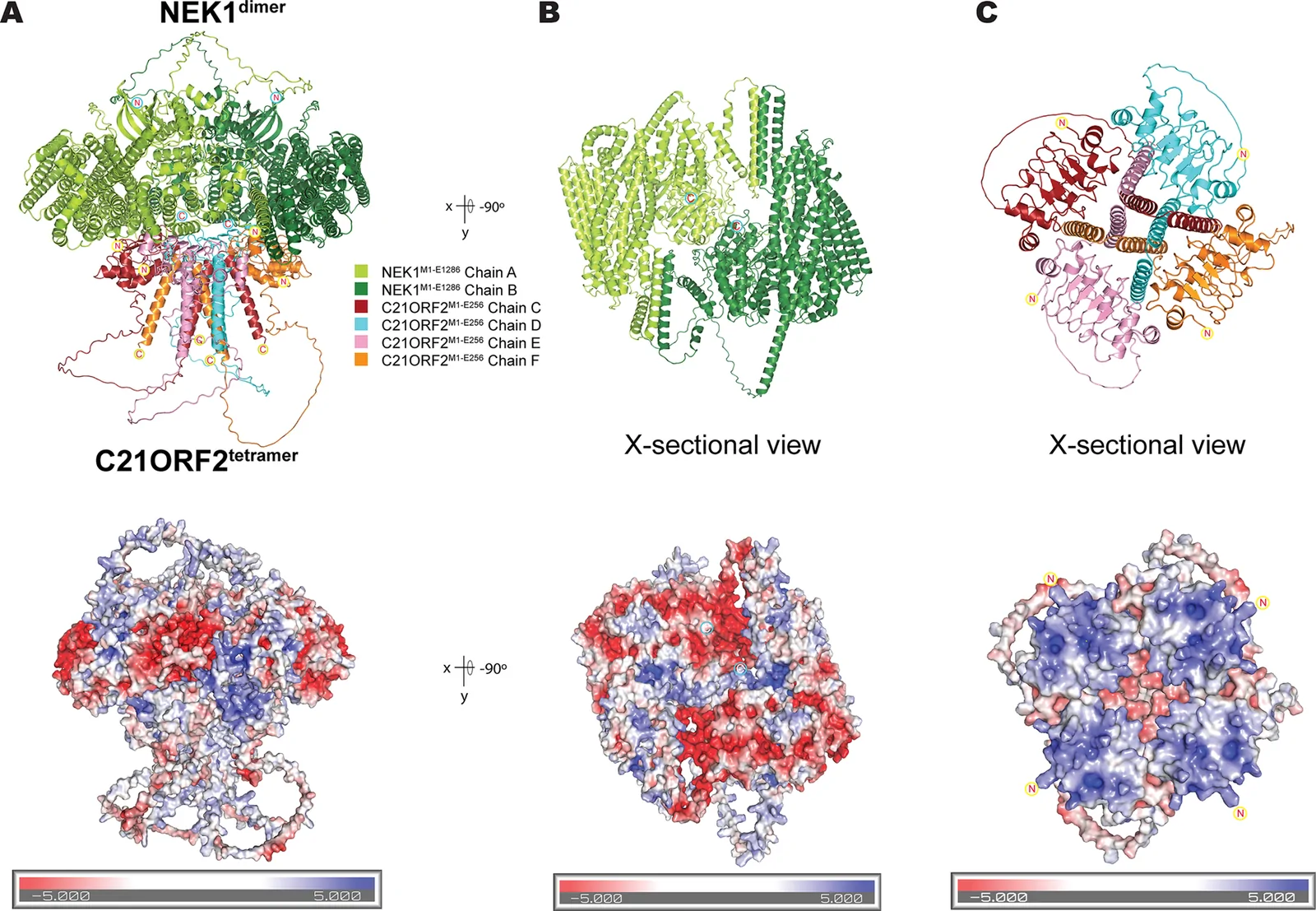
Predicted interface architecture of a putative 2:4 NEK1–C21ORF2 assembly. AlphaFold3 model comprising two full-length NEK1 chains (NEK1^M1–E1286^; chains A and B; UniProtKB Q96PY6-3) and four full-length C21ORF2 chains (C21ORF2^M1–E256^; chains C–F; UniProtKB O43822-1). The 2:4 stoichiometry modelled was informed by the mass photometry data (supplementary material, figure S16). In each panel, the cartoon representation (top) is shown above the corresponding electrostatic surface (bottom), calculated using the adaptive Poisson–Boltzmann solver (APBS) for the complete complex and for the respective interfaces, and coloured by electrostatic potential from −5 to +5 kT/e (red, negative/acidic; white, neutral; blue, positive/basic). (A) The complete assembled complex, coloured by chain: NEK1 chains A and B in light and dark green; C21ORF2 chains C–F in dark red, cyan, pink and orange. (B) Cross-sectional view of the NEK1 dimer, with the C21ORF2 chains removed and related to (A) by a −90° rotation about the x/y axis, exposing the C-terminal face that forms the interface with C21ORF2; the corresponding surface is predominantly acidic (electronegative). (C) Cross-sectional view of the four assembled C21ORF2 chains at the binding interface, with the NEK1 chains removed; the corresponding surface shows a predominantly basic (electropositive) N-terminal region, which docks onto the acidic NEK1 surface shown in (B). Chain termini are circled in cyan for NEK1 and in yellow for C21ORF2: both termini of every chain are marked in (A), whereas (B) marks the NEK1 C-termini and (C) the C21ORF2 N-termini, the ends that form the interface. Because the extended central coiled-coil region of NEK1 is predicted with low confidence, the model does not resolve whether the two kinase domains adopt a head-to-head or head-to-tail arrangement, and is presented as a hypothesis-generating framework, rather than an experimentally determined structure or stoichiometry.

The mechanism is likely to differ between the three sites. Ser14 lies over the ATP-binding pocket of its own kinase domain and is thus unlikely to access that active site intramolecularly; it is expected to be phosphorylated in *trans*. The responsible kinase could be a second NEK1 molecule or a distinct kinase: the equivalent CDK site, Thr14, is phosphorylated in *trans* by the membrane-associated kinase PKMYT1 (Myt1) rather than by the CDK itself [43,44]. Thr156, in the activation loop, may likewise be phosphorylated in *trans*, as is common for activation-loop phosphorylation, although both *cis* and *trans* mechanisms have been described and the question remains contested [35]; our data do not formally discriminate between the two. Ser418, by contrast, lies in a flexible region C-terminal to the kinase domain, 139 residues beyond its boundary, and could, in principle, fold back into the same active site, so either *cis* or *trans* phosphorylation remains possible at this site; the same argument extends to Thr1090, 811 residues C-terminal to the kinase domain in a predicted flexible region, though over a much longer tether.

More broadly, the next major challenge is to move from measuring NEK1 activation to defining the outputs of active NEK1 signalling. The NEK1-dependent electrophoretic mobility shift of C21ORF2 identifies this protein as an immediate candidate for phosphosite mapping, but the modified residues, responsible kinase and functional consequences remain unknown. Given the limited and sometimes conflicting evidence for proposed NEK1 substrates, including preprint evidence challenging RAD54 as a direct NEK1 target [45], systematic substrate and interactor mapping will be required to establish *bona fide* NEK1 substrates and distinguish direct phosphorylation events from secondary signalling effects.

Finally, the phosphorylation hierarchy suggested in our present work should be tested in disease-relevant contexts. The phosphospecific antibodies and isogenic cell lines generated here provide tools to ask whether NEK1 activation is remodelled in motor neurons, ciliated cells, DNA-damage responses, cytoskeletal stress or nucleocytoplasmic transport defects, and whether pThr156 can serve as a pharmacodynamic biomarker in future studies of NEK1-directed therapeutic strategies. Development of complementary standardized assays, including a phosphospecific pThr156 monoclonal antibody and targeted parallel reaction monitoring (PRM)-based mass spectrometry assay, would further support cross-laboratory quantification, functional variant classification, pharmacodynamic studies and patient-stratification strategies. In this sense, the tools developed here shift the field from asking whether NEK1 is phosphorylated to asking how NEK1 activation is organized, what active NEK1 phosphorylates, and how ALS-associated variants remodel those outputs.

## 4. Materials and methods

A major aim of this interdisciplinary study was to create a suite of robust NEK1 resources for the scientific community and, as such, all cell lines, reagents and antibodies generated in-house will be made available upon reasonable request from the corresponding author (A.R.M.).

All experiments used human NEK1 isoform 3 (UniProtKB Q96PY6-3**;** MANE Select transcript, 1286 amino acids long [46]), consistent with current clinical variant nomenclature standards. Accordingly, all residue numbers reported in this manuscript refer to NEK1 isoform 3. Mass spectrometry database searching used GFP-NEK1 construct entries of MRC Database 1 in which EGFP occupies construct residues 1–239, a Ser-Gly-Leu-Gly-Ser linker residues 240–244 and NEK1 isoform 3 residues 245–1530 (DU58446 for wild-type and DU80306 for the D146A kinase-dead construct, which share this numbering); raw construct positions reported in the supplementary tables are therefore converted to NEK1 isoform 3 coordinates by subtracting 244. The NEK1 isoform 3 and full-length GFP-NEK1 construct sequences are provided in supplementary material, table S1 (sequences sheet). All plasmids generated in the course of this study are listed in supplementary material, table S5, and data sheets for each unique identifier (DU number) providing direct links to cloning strategy and sequence information can be found at https://mrcppureagents.dundee.ac.uk/. Notably, because of the large size of the *NEK1* coding sequence, plasmids encoding NEK1 were propagated in NEB Stable competent cells (New England Biolabs) at 30°C to ensure plasmid stability and integrity, and purified using the NucleoBond Xtra Midi kit (Macherey-Nagel, no. 12798402). Whole plasmid sequencing was performed by Plasmidsaurus using Oxford Nanopore Technology with custom analysis and annotation. All antibodies used, including those generated in-house, are also shown in supplementary material, table S5.

### 4.1. Mammalian cell culture

All cell lines were derived from U-2 OS Flp-In T-REx cells, enabling doxycycline-inducible, single-site transgene integration. These cells were previously generated by Fulcher *et al.* [47] using the Flp-In T-REx Core Kit (Thermo Fisher Scientific, K650001). Cells were incubated at 37°C, 5% CO_2_, and routinely cultured in high-glucose Dulbecco's modified Eagle's medium (DMEM; Sigma-Aldrich, no. D5671), supplemented with: 10% (v/v) fetal bovine serum (Gibco, no. A52567, lot no. 2575645), 2 mM L-glutamine (Sigma-Aldrich, no. G7513), 1 × MEM non-essential amino acids solution (100×; Gibco, no. 11140), 1 mM sodium pyruvate (Gibco, no. 11360), penicillin (100 U ml^–1^) and streptomycin (100 µg ml^–1^) (Sigma-Aldrich, no. P4333). NEK1 knock-out Flp-In T-REx U-2 OS cells were maintained in complete medium without antibiotic selection. Stable doxycycline-inducible GFP-NEK1 re-expression lines were maintained in culture medium supplemented with 50 µg ml^–1^ Hygromycin B Gold (Invivogen, no. ant-hg-5) to maintain selection for the integrated Flp-In transgene. Cells were routinely passaged using 0.05% Trypsin/0.02% EDTA (Sigma-Aldrich, no. T3924), and for a maximum of up to 15 passages before thawing out a new low-passage vial. Monthly testing of cell culture supernatants to exclude mycoplasma contamination using the MycoAlert mycoplasma detection kit (Lonza, no. LT07-318) was carried out.

### 4.2. CRISPR-Cas9 nickase-mediated generation of NEK1 knock-out U-2 OS cell line

A transcript map of the human *NEK1* locus was constructed using NCBI genomic annotation (NC_000004.12:169390809–169614583, complement) and Ensembl annotation (ENSG00000137601). Exon 3, the first coding exon shared by all *NEK1* protein-coding isoforms, was targeted using a paired Cas9-D10A nickase strategy selected with the Sanger CRISPR webtool (https://wge.stemcell.sanger.ac.uk/find_crisprs) for low predicted off-target activity and cleavage close to the ATG start codon. The guide sequences were: sense 5′-(g)TATGTTAGACTACAGAAGAT and antisense 5′-(g)TCTCCATGATTCTTTTTCTA, where lowercase ‘g’ denotes an added base to facilitate U6 promoter-driven expression. Complementary oligonucleotides were annealed to generate BbsI-compatible inserts using the Zhang cloning method [48]. The sense guide was cloned into pBABED P U6 (DU48788) and the antisense guide into pX335 (Addgene no. 42335), yielding DU57081 and DU57089, respectively.

### 4.3. Validation of biallelic *NEK1* knock-out by amplicon-based next-generation sequencing

To validate *NEK1* knock-out at the genomic level, genomic DNA was extracted from candidate clones using the PureLink Genomic DNA Mini Kit (Thermo Fisher Scientific, no. K182001) according to the manufacturer's instructions. Amplicons spanning the CRISPR/Cas9 target site were generated by PCR using Platinum SuperFi II PCR Master Mix (Thermo Fisher Scientific, no. 12368010) in two 50 µl reactions, each containing 300 ng of genomic DNA template, 10 µM forward primer (5′-ACACTCTTTCCCTACACGACGCTCTTCCGA**TCTCCATACATTAAGTATATGCAGTATTTGGCTTTCC**-3′) and 10 µM reverse primer (5′-GACTGGAGTTCAGACGTGTGCTCTTCCGATCT**GCTCTATTGTAACTAAACCATTACTCATGAAACC**-3′), which incorporate partial Illumina adapter sequences as required for MiSeq library preparation. The expected wild-type amplicon size was 481 bp. Thermocycling: 98°C, 30 s; 30 × (98°C, 10 s; 60°C, 10 s; 72°C, 15 s); 72°C, 5 minutes. PCR products were cleaned up using Agencourt AMPure XP beads (Beckman Coulter, no. A63880) and an Invitrogen DynaMag-2 magnetic rack (Fisher Scientific, no. 17836648) at a 0.8:1 bead-to-sample volume ratio to remove primer-dimers, and DNA concentration was determined using a NanoDrop spectrophotometer. DNA was submitted to Genewiz (Azenta Life Sciences) for amplicon-based next-generation sequencing using their Amplicon-EZ service. Sequencing data were analysed to determine editing outcomes at the target locus.

### 4.4. PEI-mediated transient overexpression transfection studies

Linear PEI (MW 25 000) was purchased from Polysciences, Inc. (no. 23966-100, lot no. A862820). PEI powder was dissolved in 0.2 N HCl at 5 mg ml^–1^, and aliquots were stored at −80°C as described by Fukumoto *et al.* [49]. For transfection of one approximately 70–80% confluent NEK1 knock-out U-2 OS 15 cm dish, a mixture of 10 µg plasmid DNA and 30 µg of PEI, topped up to 1 ml with Opti-MEM medium (Gibco, no. 31985), was prepared. The transfection reaction was mixed and then incubated for 20 minutes at room temperature before being added to cells dropwise, the culture medium having been exchanged with fresh medium supplemented with 0.1 µg ml^–1^ doxycycline hyclate (Sigma-Aldrich, no. D5207). The latter is required to relieve TetR-mediated repression and permit expression of both transiently transfected and stably integrated transgenes driven by the CMV/TetO2 promoter. Cells were harvested 48 hours post-transfection.

### 4.5. Generation of a suite of cell lines stably expressing different NEK1-ALS missense mutations

To generate stable inducible cell lines, U-2 OS Flp-In T-REx NEK1 knock-out cells were co-transfected with the pcDNA5/FRT/TO expression vector containing GFP-NEK1 (either: wild-type; kinase-dead D146A in which the conserved aspartate of the DFG motif is substituted with alanine, abolishing Mg²⁺-dependent ATP positioning and phosphotransfer; V223M; R232C; R232H; R261C; R261H; A313T; R440Q; K648E; R721Q) and the pOG44 Flp recombinase plasmid (Invitrogen, no. V600520) at a 1 : 9 ratio (pcDNA5:pOG44) using PEI, as described above. Forty-eight hours post-transfection, cells were placed under dual selection with 50 µg ml^–1^ Hygromycin B Gold and 15 µg ml^–1^ Blasticidin S (Invivogen, no. ant-bl-10p) to select for stable Flp-In recombination events and maintain T-REx repressor expression, respectively. Selection medium was refreshed every 2–3 days, and resistant colonies were allowed to expand for approximately two weeks before being picked as individual clones. Stable integration was confirmed by acquisition of hygromycin resistance, maintenance of blasticidin resistance, acquisition of Zeocin sensitivity/loss of Zeocin resistance (Invivogen, no. ant-zn-5) and doxycycline-inducible expression of the transgene.

### 4.6. Cell lysis and GFP immunoprecipitation

Cells were washed with ice-cold PBS, scraped from their 15 cm dishes, pelleted, snap-frozen in liquid nitrogen and pellets stored at −80°C. Pellets were subsequently lysed in ice-cold lysis buffer comprising: 50 mM Tris-HCl pH 7.5, 150 mM NaCl, 10 mM *β*-glycerophosphate, 5 mM sodium pyrophosphate, 10% glycerol, 1% (v/v) NP-40 Alternative (Merck, no. 492016), supplemented with fresh cOmplete EDTA free Protease Inhibitor Cocktail tablet (Roche Diagnostics GmbH, no. 1187358001) and PhosSTOP tablets (Roche Diagnostics GmbH, no. 4906837001) as per the manufacturer's datasheet. Lysates were incubated on ice for 30 minutes, followed by sonication using a Bioruptor Plus sonication device (Diagenode) at high power for 15 cycles (30 seconds on / 30 seconds off) at 4°C. Lysates were clarified via centrifugation at 17 000 × *g* at 4°C for 15 minutes, and protein concentration was determined using the Pierce bicinchoninic acid assay (Thermo Fisher Scientific, no. A55864).

For GFP immunoprecipitation experiments, approximately 20 µl of ChromoTek Magnetic Particles M-270 (Proteintech) were used per 1 mg of total protein lysate. Prior to use, beads were washed three times in PBS supplemented with 0.1% NP-40, using an Invitrogen DynaMag-2 magnetic rack (Fisher Scientific, no. 17836648) for bead capture between washes. Lysates were adjusted to a total volume of 500 µl with lysis buffer and incubated with the prepared beads for 90 minutes at 4°C under end-to-end rotation. Following incubation, beads were washed once in wash buffer containing 300 mM NaCl and 0.1% NP-40, followed by two further washes in PBS supplemented with 0.1% NP-40 Alternative, with the magnetic rack used for bead capture between each wash. Immunoprecipitated proteins were eluted by resuspending beads in 2× LDS sample buffer supplemented with *β*-mercaptoethanol to a final concentration of 1%, followed by vortexing and boiling.

### 4.7. Antibody production and validation

Custom sheep polyclonal antibodies against NEK1 pSer14, pSer418 and pThr156 were generated by MRC PPU Reagents and Services (University of Dundee) using phosphopeptide antigens synthesized by Peptides and Elephants GmbH (Hennigsdorf, Germany). Corresponding non-phosphorylated peptides were used for specificity testing and, where indicated, depletion. Peptide IDs, sequences, theoretical masses, working concentrations and validation details are provided in supplementary material, table S5. These antibodies complement previously generated sheep polyclonal antibodies against total NEK1 and C21ORF2 [28].

Sheep immunizations were performed under UK Home Office Project Licence PP9490726 following review and approval by the University of Edinburgh's Roslin Animal Welfare and Ethical Review Body (AWERB). Peptides were conjugated to keyhole limpet haemocyanin and bovine serum albumin through the terminal cysteine residue, with an Ahx spacer included where indicated, as described by Harlow & Lane [50]. Sheep were immunized with antigen followed by up to four further injections administered 28 days apart, with bleeds performed 7 days after each injection.

Antisera were heat-treated, filtered and affinity-purified on peptide-coupled resin. For antibodies purified against phosphorylated peptide alone, serum was applied to the cognate phosphopeptide resin, washed extensively and eluted under acidic conditions. For the pThr156 antibody, tandem depletion was used to improve phosphospecificity: serum was first passed over resin coupled to the non-phosphorylated peptide to deplete antibodies recognizing the peptide backbone independently of phosphorylation, and the depleted flow-through was then applied to resin coupled to the phosphorylated pThr156 peptide. Bound antibodies were eluted under acidic conditions, immediately neutralized, dialysed into PBS, quantified by Bradford assay, adjusted to >0.1 mg ml^–1^ and stored in aliquots at −20°C.

Dot blot analyses were performed to evaluate antibody specificity and sensitivity against phosphorylated and non-phosphorylated cognate peptides. Serial peptide dilutions from 1000 to 0.1 ng µl^–1^ were spotted onto nitrocellulose strips (Amersham Protran 0.45 µm; Cytiva, no. 10600002) in two successive 1 µl applications per spot. Membranes were air-dried, blocked in 5% (w/v) skimmed milk (Marvel Original) in TBS-T [1× TBS-T: 80 mM Tris-HCl, 150 mM NaCl, 0.1% Tween-20, pH 7.5], incubated overnight at 4°C with purified antibody at 1 µg ml^–1^, washed in TBS-T, incubated with LI-COR secondary antibody (LI-COR Biosciences, no. 926-32214) at 1 : 20 000, and imaged using a LI-COR Odyssey CLx instrument.

### 4.8. Immunoblotting

Samples were subjected to SDS-polyacrylamide gel electrophoresis using NuPAGE Bis-Tris Midi Protein Gels (Thermo Fisher Scientific) and NuPAGE MOPS SDS Running Buffer (Thermo Fisher Scientific, no. NP000102), followed by transfer onto nitrocellulose membranes (Amersham Protran 0.45 µm; Cytiva, no. 10600002). Membranes were blocked for 1 hour at room temperature in 5% (w/v) skimmed milk (Marvel Original) in TBS-T and incubated overnight at 4°C with the indicated antibodies diluted in 5% milk/TBS-T. For immunoblot detection of NEK1 autophosphorylation sites, phosphospecific antibodies were used at 1 µg ml^–1^, with the exception of the anti-NEK1 pThr156 antibody, which was used at 0.5 µg ml^–1^. Phosphospecific antibodies were pre-incubated with the corresponding non-phosphorylated peptide at 10 µg ml^–1^ for 60 minutes at 4°C before application to membranes, to assess whether signal detection depended on phospho-epitope recognition rather than recognition of the non-phosphorylated peptide backbone. The next morning, the membranes were washed three times for 5 minutes in TBS-T. Highly cross-adsorbed H+L secondary antibodies (Life Technologies) conjugated to (IRDye 800CW or IRDye 680RD Infrared Dyes) were used at 1 : 20 000 in 5% skimmed milk for 1 hour. The membranes were then washed three times in TBS-T at room temperature and then imaged using a LI-COR Odyssey CLx instrument. For pThr156 immunoblotting of stably expressed ALS variants, SuperSignal West Atto Ultimate Sensitivity Substrate (Thermo Fisher Scientific, no. A38554) was used according to the manufacturer's instructions with HRP-conjugated secondary antibody at 1 : 250 000, and signal was acquired using a ChemiDoc MP imaging system (Bio-Rad Laboratories). For immunoblot quantification, blots were analysed using Fiji v2.16.0/1.54p (ImageJ2, National Institutes of Health [51]). Band intensities were measured after background subtraction using identical regions of interest across samples within each blot. Phosphorylation signals were normalized to the corresponding total NEK1 signal, and the resulting values were expressed relative to wild-type NEK1, which was set to 1.0 within each independent experiment.

### 4.9. Phosphosite mapping via liquid chromatography mass spectrometry (LC-MS/MS)

Phosphosite mapping was performed using two different approaches (overexpression and stable expression) in a NEK1 knock-out cellular background; moreover, two different mass spectrometry acquisition methods were deployed—HCD resulting in proton-directed bond cleavage, and EThcD (supplemental activation 35%). The latter electron-driven fragmentation technique affords complementary b/y and c/z ion series, thus increasing the unambiguous localization of NEK1-dependent phosphosites compared with standard HCD, which is known to be problematic for the interrogation of labile post-translational modifications [52].

Accordingly, U-2 OS NEK1 knock-out cells stably re-expressing GFP-tagged NEK1 (either wild-type or kinase-dead) were induced with doxycycline (0.1 µg ml^–1^) for 24 hours prior to harvest. This dose was selected to give modest expression rather than maximal induction; in our hands, single-copy Flp-In reconstitution of NEK1 in this knock-out background gives NEK1 levels comparable to those of endogenous NEK1 in parental U-2 OS cells. U-2 OS NEK1 knock-out cells overexpressing GFP-tagged NEK1 (wild-type, kinase-dead, S155A, T156A, S155A and T156A double mutant) were generated as outlined in the aforementioned section. Following GFP immunoprecipitation using ChromoTek Magnetic Particles M-270 (Proteintech), samples were eluted in 2× LDS buffer (with *β*-mercaptoethanol at a final concentration of 1%). Samples were resolved by SDS-PAGE on NuPAGE 8% or 4–12% Bis-Tris Midi Gels (Thermo Fisher Scientific, no. WG1402BOX) using NuPAGE MOPS SDS Running Buffer (Thermo Fisher Scientific, no. NP000102) at approximately 120 V for 1.5–2 hours. Gels were stained with the Colloidal Blue Staining Kit (Thermo Fisher Scientific, no. LC6025) for 3–6 hours and destained overnight in LC-MS grade water. Gel bands corresponding to NEK1 were excised, cut into approximately 1 mm³ cubic pieces and incubated in 50 mM TEAB (Fisher Scientific, no. 15215753) to permit reduction with 5 mM TCEP (15 minutes, 55°C using a thermomixer; Thermo Fisher Scientific, no. 10657344), followed by alkylation with 20 mM iodoacetamide (30 minutes, 30°C, in the dark).

Using a standard operating procedure [53], the cut gel pieces were then washed in water (Fisher Scientific, no. 10434902) for 10 minutes using a thermomixer at room temperature. Gel pieces were dehydrated in 100% acetonitrile (Fisher Scientific, no. 10055454) and rehydrated in 50 mM ammonium bicarbonate, repeated three times, followed by overnight incubation at 4°C with gentle shaking to wash out the Coomassie Brilliant Blue stain. Once the gel pieces were colourless, they were further dehydrated with 100% acetonitrile, and then were air-dried. Digestion was set up by incubating the gel pieces in Trypsin/Lys-C Protease Mix (Thermo Fisher Scientific, no. A41007; 2 µg enzyme per sample) in 50 mM TEAB (Fisher Scientific, no. 15215753) and 2.5 mM CaCl₂ for 1 hour at 37°C, followed by overnight digestion (12–14 hours) at 30°C. The following morning, the supernatant containing digested peptides was collected in low-binding tubes. Gel pieces were shrunk by addition of acetonitrile (25°C for 15 minutes of incubation). The supernatants were collected (pooled with previous). The gel pieces were next rehydrated with 80% ACN + 0.5% TFA (25°C for 15 minutes incubation). The supernatant was pooled and snap-frozen on dry ice, and vacuum-dried using a Thermo Scientific Savant SpeedVac SPD140DDA vacuum concentrator. Samples were then stored at –20°C before LC-MS/MS analysis.

LC-MS/MS analysis was performed on an Orbitrap Lumos Tribrid mass spectrometer (Thermo Scientific, San Jose, CA) equipped with an EASY-nESI source (Thermo Scientific) and coupled to a Dionex Ultimate 3000 nano-LC system. Kinase-dead samples were always injected first to minimize the possibility of phosphopeptide carryover from wild-type samples into kinase-dead controls. Peptides were trapped on an Acclaim PepMap 100 C18 trap column (150 mm × 0.1 mm, 5 µm; Thermo Fisher Scientific, no. 164750) and separated on a PepMap RSLC C18 reverse-phase analytical column (2 µm, 100 Å, 75 µm × 50 cm) at a flow rate of 300 nl min⁻¹. Peptides were eluted using a 40-minute linear gradient from 97% solvent A (0.1% v/v formic acid in water) to 35% solvent B (80% acetonitrile, 0.08% formic acid), followed by a ramp to 95% solvent B at 47 minutes, within a total run time of 70 minutes. The instrument was operated in positive ion data-dependent top speed mode with a cycle time of 3 seconds. Spray voltage was set to 2 kV, RF lens level to 30%, and ion transfer tube temperature to 300°C. Full-scan MS spectra were acquired in the Orbitrap over a mass range of 375–1500 m/z at a resolution of 120 000 at 200 m/z with AGC target set to Standard. Precursor ions above an intensity threshold of 50 000 with charge states 2–7 were selected for MS2 fragmentation using an isolation width of 1.6 m/z and a dynamic exclusion duration of 30 seconds. Fragmentation was performed in separate runs using both HCD (normalized collision energy 30%) and EThcD (supplemental activation 35%) methods. All MS2 spectra were recorded in centroid mode with an AGC target set to Standard and a maximum fill time of 50 ms.

Raw data were processed using Proteome Discoverer 2.4 (Thermo Scientific) with Mascot (v2.8.3, Matrix Science, www.matrixscience.com) as the search engine against our own in-house database (MRC Database 1), with no download date or taxonomy restriction applied. A precursor mass tolerance of 10 ppm and fragment mass tolerance of 0.06 Da were applied, with a maximum of two missed cleavages permitted. Carbamidomethylation of cysteine was set as a fixed modification, while the oxidation and dioxidation of methionine, and phosphorylation of serine, threonine and tyrosine were set as variable modifications. Phosphosite localization was assessed using both Mascot Site Analysis, which calculates localization probabilities from the delta between Mascot scores of competing site assignments (MD-score/Mascot PTM confidence [54]), and the ptmRS node from Proteome Discoverer [55]; for ptmRS, a mass tolerance of 0.5 was applied, and neutral loss peaks were considered for both HCD and EThcD fragmentation. Both Mascot PTM confidence and ptmRS localization probabilities were extracted from the raw data and are reported in the compiled supplementary phosphosite tables. For the curated high-confidence analyses presented in the manuscript, phosphosite assignments were considered high-confidence where peptides achieved a Mascot ion score >19 together with ptmRS ≥85%. In cases where ptmRS was not computed by Proteome Discoverer, sites with Mascot PTM confidence ≥85% were retained and flagged accordingly in the supplementary tables. Lower-confidence or exploratory detections are retained separately within some supplementary worksheets for completeness, but were not used for high-confidence overlap analyses or classification of candidate autophosphorylation sites. Phosphosites detected exclusively via multiply-phosphorylated peptides were excluded where the co-phosphorylated residue on the same peptide was independently localized by a singly-phosphorylated species. The Max Mascot PTM Conf and Max ptmRS values reported may derive from different peptide-spectrum matches: the former is taken from the PSM with the highest Mascot PTM Conf at that site, while the latter represents the maximum ptmRS probability across all singly-phosphorylated PSMs. The Mascot ion score is taken from the same PSM that yielded the highest Mascot PTM Conf. For a subset of sites detected in recombinant protein datasets, phosphopeptides containing methionine were observed exclusively in their Met-oxidized (M+16) form; these are flagged in the curation notes of the supplementary tables. Localization confidence for these sites was assessed from fragment ions unaffected by Met oxidation, and all sites are confirmed by non-oxidized phosphopeptides in the cell-based datasets. Phosphosite data were then conveniently visualized using AlphaMap [56], which renders sites on built-in AlphaFold2-predicted structural models, enabling spatial interpretation of phosphorylation sites relative to domain architecture.

### 4.10. Extracted ion chromatogram analysis

XICs were generated from raw mass spectrometry data using Skyline (v25.1, MacCoss Lab, University of Washington [57]). Thermo.RAW files were imported into Skyline, and NEK1 phosphopeptides of interest, along with their corresponding fragment ion transitions, were configured as targets. Peak detection was performed automatically by Skyline, and peak areas were extracted for quantitative comparison across conditions. Peak boundaries were manually inspected and adjusted where necessary to ensure accurate area integration. To account for differences in sample loading and protein recovery between conditions, peak areas were normalized to the total ion current (TIC) of each LC-MS/MS run using Skyline's Total Ion Current normalization function. XIC analyses were used for relative comparison of phosphopeptide abundance rather than absolute phosphorylation stoichiometry.

### 4.11. Expression and purification of recombinant NEK1, and preparation of samples for mass spectrometry

Human *NEK1* (construct DU79459), *NEK1^D146A^*/*C21ORF2* (DU79595), or *NEK1/C21ORF2* (DU79475) carrying an N-terminal GFP-RV3C tag were cloned into the pFBDMb vector and transformed into DH10Bac cells. White colonies were selected on insect selection plates and cultured in TB medium containing kanamycin (10 µg ml^–1^), tetracycline (5 µg ml^–1^) and gentamicin (7 µg ml^–1^). Bacmid DNA was prepared using Qiagen miniprep reagents, precipitated with isopropanol and washed with 70% ethanol. Bacmid DNA was transfected into *Spodoptera frugiperda* Sf9 cells cultured in Sf-900 II SFM medium (Thermo Fisher Scientific, no. 10902088), and P0 virus was harvested after 7 days and amplified in Sf9 cells for 5 days.

For protein expression, Sf9 cells were seeded at 2.5 × 10^6^ cells ml^–1^ in Sf-900 II SFM medium supplemented with L-glutamine and infected with virus at a 1 : 50 dilution. Cells were harvested after 48–52 hours by centrifugation, snap-frozen in liquid nitrogen and stored at −80°C. Cell pellets were thawed and resuspended in six pellet volumes of lysis buffer containing 50 mM HEPES pH 7.5, 1 mM EDTA, 1 mM EGTA, 1 mM AEBSF, 10 µg ml^–1^ leupeptin, 1 mM TCEP and 1 mM DTT, and incubated for 20 minutes to allow osmotic swelling. Cells were lysed using a tight-fitting Potter–Elvehjem homogenizer (2 × 10 strokes), NaCl was adjusted to 250 mM, and insoluble material was removed by centrifugation at 40 000 × *g* for 25 minutes.

Clarified lysates were incubated with anti-GFP Sepharose for 2.5 hours at 4°C. The resin was washed seven times with wash buffer containing 50 mM HEPES pH 7.5, 250 mM NaCl, 1 mM EDTA, 1 mM EGTA, 1 mM AEBSF, 10 µg ml^–1^ leupeptin, 1 mM TCEP and 1 mM DTT. The second and third washes were supplemented with 10 mM MgCl₂ and 2 mM ATP to remove HSP70, followed by additional washes to remove residual Mg-ATP. Bound protein was cleaved overnight at 4°C on-resin using His-SUMO-GFP-RV3C protease. Ni-Sepharose resin was subsequently added to remove the His-tagged protease, and purified NEK1 protein was collected, concentrated, aliquoted, snap-frozen in liquid nitrogen and stored at −80°C.

For mass spectrometry analysis, purified proteins were resolved by SDS-PAGE and visualized using Coomassie Brilliant Blue staining. Protein bands corresponding to NEK1 were excised and cut into approximately 1 mm³ pieces. Gel pieces were transferred to 1.5 ml microcentrifuge tubes, destained with 40% methanol in 50 mM triethylammonium bicarbonate (TEAB; pH 8.0), and alkylated using 50 mM iodoacetamide in 50 mM TEAB containing 2 mM TCEP. Gel pieces were dehydrated with 100% methanol, after which the solvent was removed, and proteins were digested using Trypsin Gold, Mass Spectrometry Grade (Promega, no. V5280; 10 ng µl^–1^ final concentration in 50 mM TEAB) for at least 16 hours at 30°C. The resulting peptide solution was transferred to fresh tubes, and gel pieces were briefly incubated in 100% methanol to maximize peptide extraction. Methanol extracts were pooled with the peptide solution, leaving the gel pieces behind. Samples were dried completely using a SpeedVac concentrator and submitted for mass spectrometry analysis. LC-MS/MS acquisition was performed using the same instrument, gradient, and data-dependent top-speed parameters as described above for cell-based samples, with both HCD and EThcD fragmentation applied across three replicates. Raw data were processed using Proteome Discoverer 2.4 with Mascot (v2.8.3) using the same search parameters, with phosphosite localization assessed by ptmRS (threshold ≥85%) and Mascot site analysis as described.

### 4.12. Size-exclusion chromatography

NEK1 and the NEK1–C21ORF2 complex were expressed in Sf9 insect cells and purified by anti-GFP affinity chromatography followed by RV-3C (PreScission) protease cleavage to remove the GFP tag. Size-exclusion chromatography was performed at 4°C on an ÄKTA Pure system (Cytiva) using a Superose 6 Increase 10/300 GL column (column volume 23.6 ml) at a flow rate of 0.3 ml min^–1^ in running buffer (50 mM Tris-HCl pH 7.5, 150 mM NaCl, 0.5 mM TCEP). A sample volume of 200 µl was injected onto the column. Fractions of 0.5 ml were collected into a 96-well deep-well plate and analysed by SDS-PAGE and Western blotting to confirm protein identity. Fraction B2 (NEK1 alone, eluting at 8.4 ml) and fraction B9 (NEK1–C21ORF2, eluting at 12.3 ml) were selected for mass photometry analysis.

### 4.13. Mass photometry

Mass photometry measurements were performed using a OneMP mass photometer (Refeyn Ltd, Oxford, UK). High-precision borosilicate glass coverslips (24 × 50 mm, No. 1.5H, 170 ± 5 µm; Marienfeld-Superior, Lauda-Königshofen, Germany) were used as measurement surfaces, with sample wells formed using CultureWell reusable gaskets (Grace Bio-Labs, CW-50R-1.0, Bend, OR, USA) cut to size. All buffers were filtered through a 0.2 µm membrane filter and equilibrated to room temperature prior to use.

NEK1 alone and the NEK1–C21ORF2 complex (produced from recombinant proteins) were diluted in freshly prepared 50 mM Tris-HCl pH 7.5, 150 mM NaCl and 0.5 mM TCEP to concentrations in the range of 5–100 nM to identify the optimal concentration for single-molecule detection. For each measurement, 10 µl of sample was pipetted into the well containing 10 µl of buffer, and data acquisition was initiated immediately. Measurements were acquired for 60 seconds. Mass calibration was performed using thyroglobulin, aldolase, conalbumin and ovalbumin (all Cytiva). Contrast values were converted to mass (kDa) using DiscoverMP software (Refeyn Ltd), and mass histograms were fitted with Gaussian distributions to determine the mean mass and relative abundance of each detected species. The ‘merge’ feature was used to merge two technical replicates for each of NEK1 alone and NEK1–C21ORF2 complex.

### 4.14. Phosphosite dataset comparison and visualization

Phosphorylation sites identified by LC-MS/MS from cell-based and recombinant protein datasets were compiled and cross-referenced against all NEK1 isoform 3 entries in PhosphoSitePlus [32]. For the cell-based dataset, sites were included if they met a ptmRS localization probability ≥85% in at least one of the four acquisition conditions, or if they were independently detected with consistent site localization in two or more separate acquisition conditions; sites with ambiguous residue assignment (comparable localization probabilities for two residues within the same peptide) were excluded (*n* = 34 of 48 detected sites; six sites excluded for sub-threshold localization in all conditions, eight excluded for ambiguous assignment). For the recombinant dataset, only sites detected in 3/3 biological replicates in wild-type NEK1 were included (*n* = 26); sites additionally present in kinase-dead NEK1 were retained in the total but annotated separately as constitutive or trans-phosphorylated rather than kinase-activity-dependent candidates. Intersection analysis across the four datasets was visualized as an UpSet plot [58] (figure 3A) using custom code. Sites present in both cell-based and recombinant wild-type datasets, but absent from PhosphoSitePlus, were reviewed according to cross-dataset support and localization confidence. pThr1090 and pSer295 were classified as the strongest candidate sites not currently annotated in PhosphoSitePlus, whereas pSer736 was retained as a lower-confidence provisional site because its cell-based support was limited and recombinant localization confidence was sub-threshold; sites detected exclusively in the recombinant dataset were noted separately and not classified as cell-validated.

### 4.15. Statistical analysis

Data were graphed using GraphPad Prism 11. Phospho-NEK1 signal was normalized to total NEK1 for each sample and subsequently to the wild-type condition within each independent experimental replicate. Each NEK1 construct was compared with wild-type using a two-sided one-sample *t*-test against a hypothetical mean of 1.0. *p*-values are reported unadjusted for multiple comparisons because each ALS-associated variant was treated as a pre-specified independent hypothesis. Data are presented as mean ± SEM from three independent biological experiments.

### 4.16. Bioinformatic determination of NEK1 domain structure and its sequence evolutionary conservation across the NEK family

An up-to-date NEK1 domain structure was constructed by examining human NEK1 isoform 3 (UniProtKB Q96PY6-3) using AlphaFold3 [59], together with NEK1′s known crystal structure of the kinase domain (PDB: 4APC / 4B9D [26]). Acidic and basic regions were determined by analysing the multiple sequence alignment in Jalview (version 2.11.5.1 [60]). Regions rich in acidic or basic residues were projected onto the schematics. For the coiled-coil regions, manual inspection of the prediction files generated by pCOILS and MARCOIL algorithms (accessed via the MPI Bioinformatics Toolkit available via: https://toolkit.tuebingen.mpg.de/; [61,62]) was performed to consistently identify residue ranges predicted as high-confidence coiled-coil regions by both algorithms [63–66]. Boundaries were defined based on the overlap between the two prediction methods. In the pCOILS analysis, which uses the Lupas coiled-coil scoring matrix, predictions were evaluated using sliding windows of 14, 21 and 28 residues. Regions were considered significant when both window 21 and window 28 probabilities exceeded 0.5 over a continuous stretch of residues, indicating a stable coiled-coil pattern. Predictions from MARCOIL, which employs a hidden Markov model (HMM) for detecting coiled-coil sequences, were considered significant when the coiled-coil probability was at least 60%. Nuclear localization signal was predicted using cNLS Mapper, offering motif-level prediction (available via: https://nls-mapper.iab.keio.ac.jp/cgi-bin/NLS_Mapper_form.cgi; [67]), and NucPred, offering protein-level prediction (available via: https://nucpred.bioinfo.se/nucpred/; [68]). The nuclear export signal was predicted by aligning the predicted leucine-rich consensus sequences from mouse NEK1 contained within [24], which used NetNES v1.1 [69], with human NEK1 isoform 3′s FASTA sequence (this is +28 amino acids different from mouse NEK1, which is similar to human NEK1 isoform 1). The C21ORF2 interaction domain (CID) was previously determined experimentally [28] and was, therefore, used as an experimentally defined domain boundary.

For NEK1–C21ORF2 complex modelling, AlphaFold3 was used to model a putative assembly containing two NEK1 chains and four C21ORF2 chains. This stoichiometry was selected as a hypothesis-generating model guided by the mass determined by mass photometry for the higher-order species resolved by size-exclusion chromatography, and was not treated as an experimentally determined stoichiometry. Electrostatic surface potentials were calculated using APBS for the complete assembly and for the respective interfaces.

NEK1 protein sequences from 26 vertebrate species were retrieved and aligned using Multiple Alignment using Fast Fourier Transform (MAFFT) [70] with default parameters, generating a multiple sequence alignment of 1330 columns; alignment of the 1286-residue human NEK1 query required the introduction of 44 gaps distributed across 20 stretches, the largest being a nine-residue insertion after human residue 680 that was present in all birds, but absent from mammals. Per-residue evolutionary conservation scores were calculated using a locally installed version of ConSurf kindly provided by Professor Nir Ben-Tal, Tel-Aviv University, Israel, with human NEK1_isoform3 as the query and the multiple sequence alignment as input [71,72]; ConSurf assigned each residue a discrete conservation grade from 1 (most variable) to 9 (most conserved).

ConSurf grades were mapped from ungapped residue numbering (1–1286) to Jalview alignment columns (1–1330) using a custom Python script, producing a Jalview annotation file (.jva) with the standard nine-colour ConSurf palette (gap columns left uncoloured). Per-residue colouring was applied across all 26 sequences via *Colour → By Annotation* using the original ConSurf colours. Maximum-likelihood phylogenetic reconstruction was performed using IQ-TREE v3.0.1 [73] through the CIPRES Science Gateway [74], with ModelFinder [75] model selection, 1000 ultrafast bootstrap replicates [76] and 1000 SH-aLRT replicates. The best-fit model according to BIC was Q.BIRD+I+G4. The resulting tree was rooted using the avian taxa as the outgroup. Human NEK1 isoform 3 residue 721 (UniProtKB Q96PY6-3) was mapped to column 745 of the MAFFT alignment by iterating over the gapped human sequence and counting non-gap residues; residue states at this column were extracted for all taxa and used for ancestral-state reconstruction in PastML [77] using MAP prediction under the F81 model.

### 4.17. Bioinformatic identification of NEK1-ALS missense variants

Prioritization of genetic variants was performed using summary statistics from a recent exome-wide rare variant association study involving 13 138 ALS patients and 69 775 controls [11]. Variants were annotated with a pass_callrate flag indicating whether they met the stringent call-rate quality control thresholds required for unbiased exome-wide discovery analyses. An additional set of variants failing these thresholds, but present in genes curated by the ALS Gene Curation Expert Panel (GCEP), was also evaluated in the original study, consistent with the rationale that relaxing call-rate filters for established ALS-associated loci enables more comprehensive assessment of known disease genes ([11], figure 1B therein). For the purposes of variant prioritization in the present study, only variants passing the stringent call-rate filters (pass_callrate = TRUE) were considered, and missense variants in *NEK1* nominally associated with ALS risk (*p* < 0.05) were selected for functional characterization. This threshold was used to prioritize variants with genetic evidence of enrichment for functional follow-up, rather than to define pathogenicity. The complete single-variant association statistics for *NEK1*, including both pass_callrate = TRUE and FALSE variants, are provided in supplementary material, table S6. Statistical associations with ALS risk were tested at the variant, gene and domain level using Firth logistic regression and the Rare Variant Association Toolkit (RVAT) [78]. All association testing analyses included correction for sex, genome-wide burden of synonymous variants and the first 10 principal components derived from principal component analysis of common variant profiles. Variant annotations were generated using SnpEff for nonsense and frameshift mutations [79] and splice variants via dbscSNV [80], with annotations based on Ensembl release 105 for build GRCh38 of the human genome.

## Supplementary Material



## Data Availability

All data needed to evaluate the conclusions in the paper are present in the paper and/or the supplementary material. Primary/source data associated with the main and supplementary figures, sequencing data for CRISPR/Cas9-generated U-2 OS NEK1 knock-out cells, and sequencing data for all plasmids generated or used in this study are publicly deposited in the Zenodo Data Repository [81]. Annotated peptide spectra shown in the supplementary data are Mascot-derived peptide-spectrum match reports rather than raw mass spectra. The mass spectrometry proteomics data have been publicly deposited to the ProteomeXchange Consortium via the PRIDE [82] partner repository with the dataset identifier PXD079815. A step-by-step phosphomapping protocol is published in Bio-protocol [83]. One of the major aims of this work was to create a suite of robust resources for the community; accordingly, all cell lines, reagents and antibodies generated in-house will be made available upon reasonable request from the corresponding author, A.R.M. Supplementary material is available online [84].
