# Supplementary material for: NEK1 autophosphorylation is disrupted by amyotrophic lateral sclerosis-associated missense variants: activity biomarkers and structural insights
Source: Open Biol. 2026 Oct 28;16(10):260209. doi: 10.1098/rsob.260209 (PMC13714390; doi:10.1098/rsob.260209)
Supplement: Supplementary file 1 [file rsob.260209.zip › rsob.260209/rsob260209_si_001.pdf]

FIG S1

A

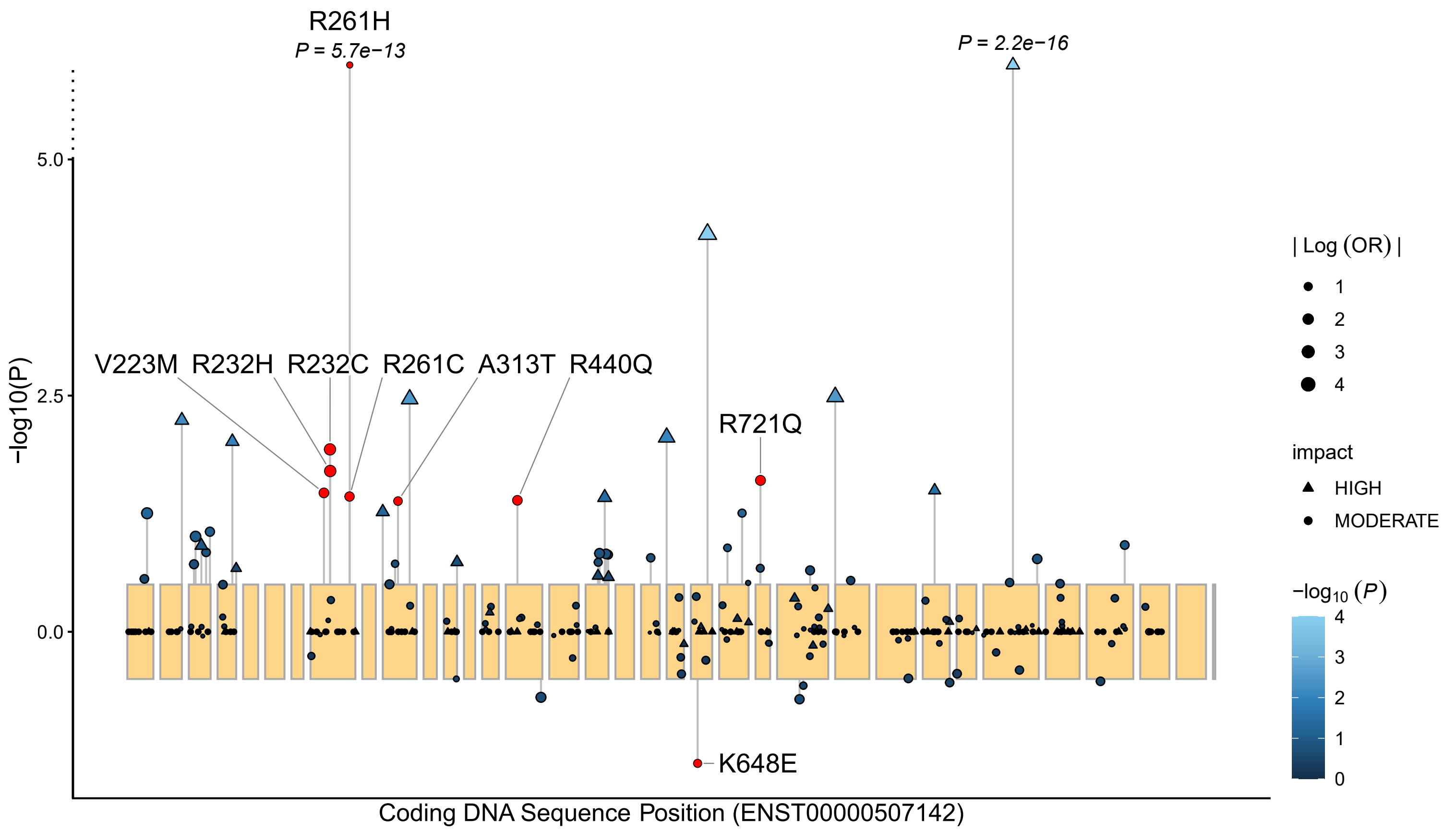

R261H
P = 5.7e−13
P = 2.2e−16
V223M R232H R232C R261C A313T R440Q
R721Q
K648E
5.0
2.5
0.0
-log10(P)
Coding DNA Sequence Position (ENST00000507142)
| Log (OR) |
1
2
3
4
impact
HIGH
MODERATE
−log10 (P)
4
3
2
1
0

B

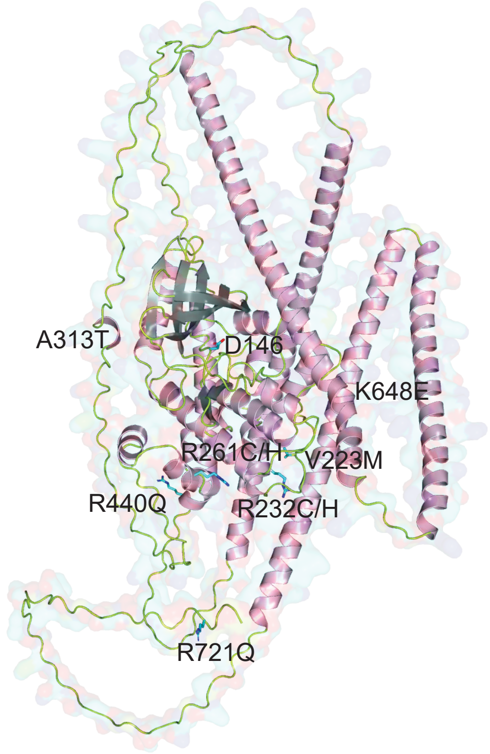

A313T
D146
K648E
R261C/H
V223M
R440Q
R232C/H
R721Q
