# Supplementary material for: NEK1 autophosphorylation is disrupted by amyotrophic lateral sclerosis-associated missense variants: activity biomarkers and structural insights
Source: Open Biol. 2026 Oct 28;16(10):260209. doi: 10.1098/rsob.260209 (PMC13714390; doi:10.1098/rsob.260209)

FIG S2

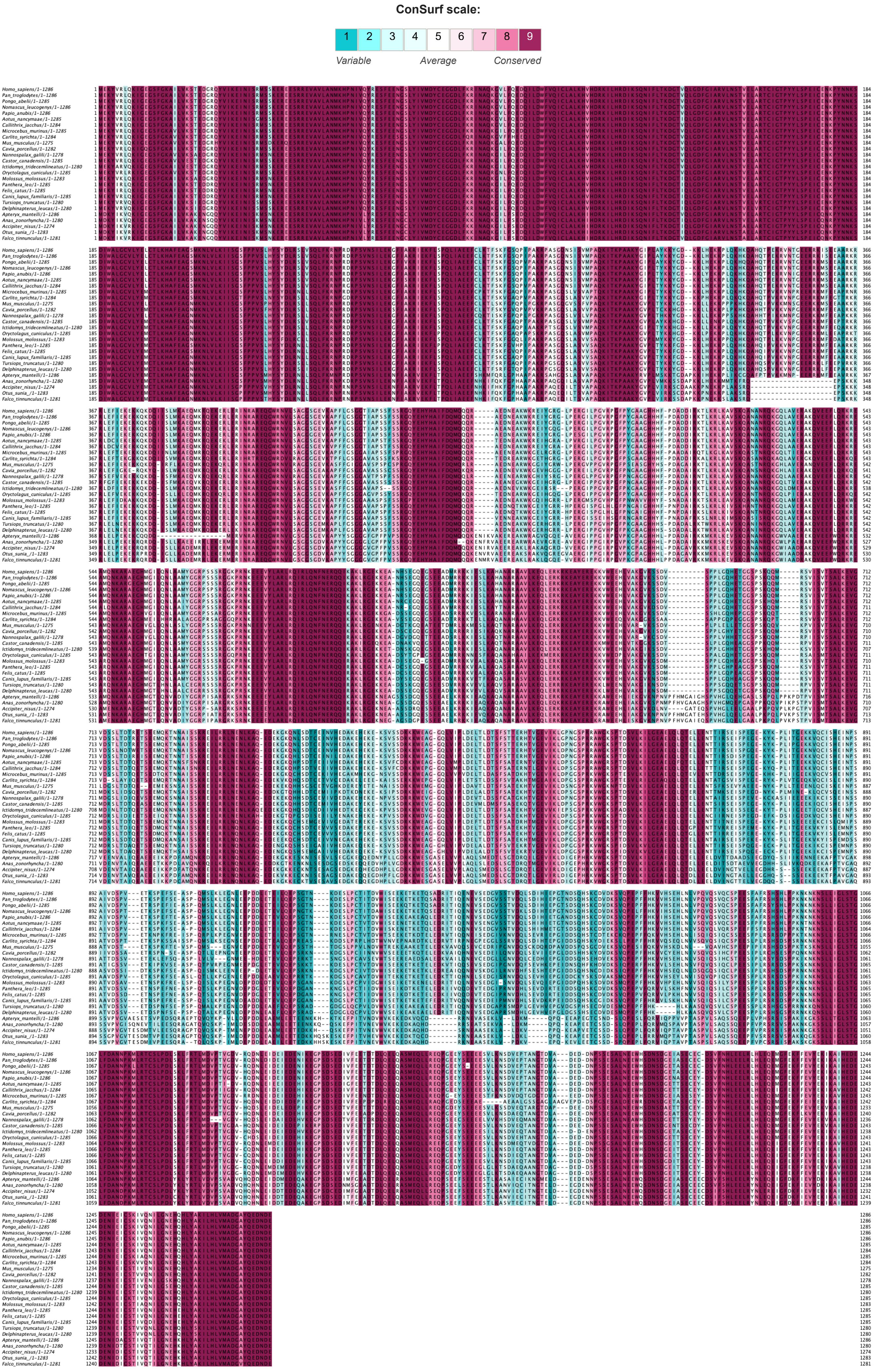

Supplement: Supplementary file 1 [file rsob.260209.zip › rsob.260209/rsob260209_si_002.pdf]
