# Supplementary material for: NEK1 autophosphorylation is disrupted by amyotrophic lateral sclerosis-associated missense variants: activity biomarkers and structural insights
Source: Open Biol. 2026 Oct 28;16(10):260209. doi: 10.1098/rsob.260209 (PMC13714390; doi:10.1098/rsob.260209)
Supplement: Supplementary file 1 [file rsob.260209.zip › rsob.260209/rsob260209_si_003.pdf]

FIG S3

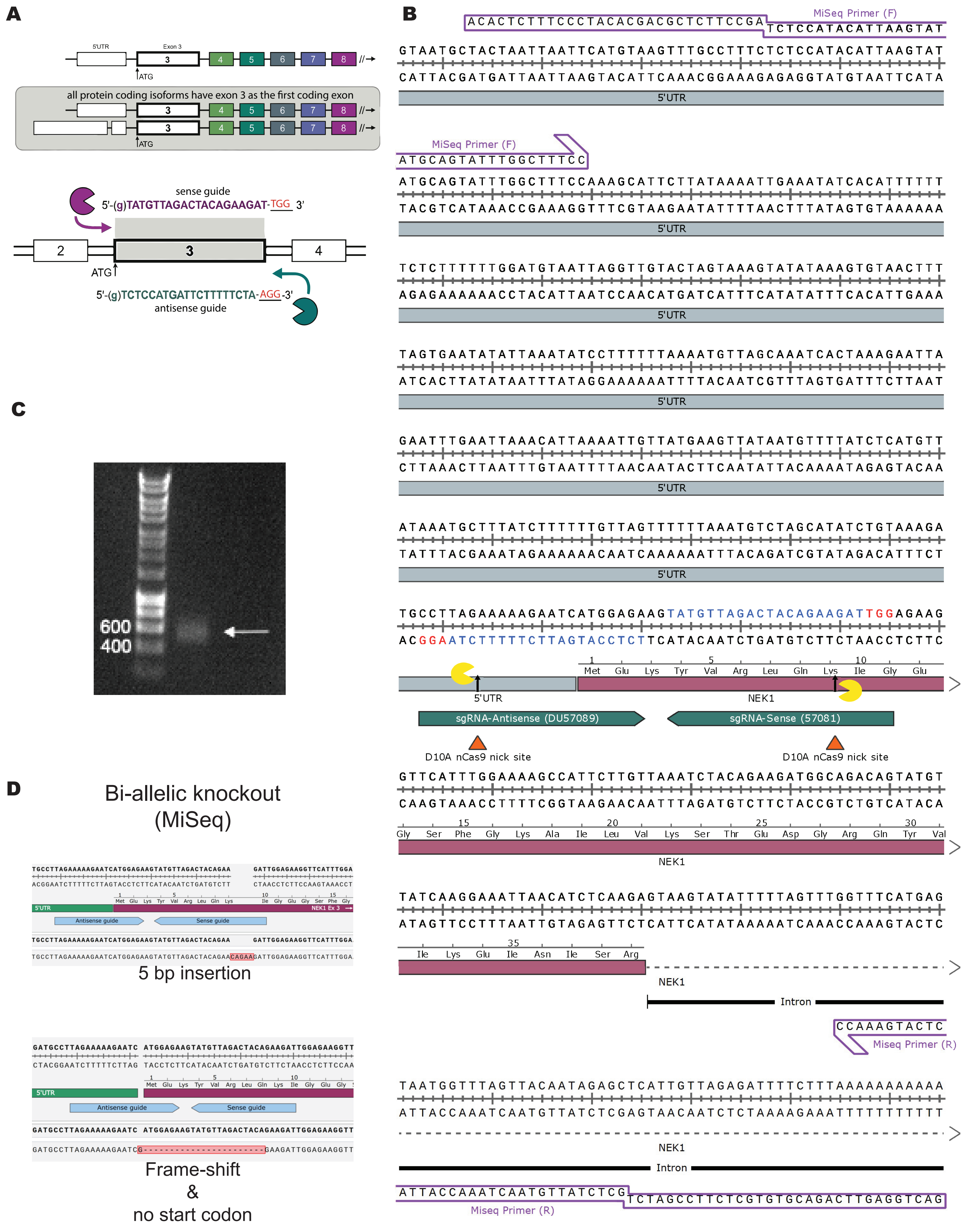
A
5'UTR
Exon 3
ATG
all protein coding isoforms have exon 3 as the first coding exon
sense guide
5'-(g)TATGTTAGACTACAGAAGAT-TGG 3'
ATG
5'-(g)TCTCCATGATTCTTTTTCTA-AGG-3'
antisense guide
B
MiSeq Primer (F)
ACACTCTTTCCCTACACGACGCTCTTCCGA
TCTCCATACATTAAGTAT
GTAATGCTACTAATTAATTCATGTAAGTTTGCCTTTCTCTCCATACATTAAGTAT
CATTACGATGATTAATTAAGTACATTCAAACGGAAAGAGAGGTATGTAATTCATA
5'UTR
MiSeq Primer (F)
ATGCAGTATTTGGCTTTCC
ATGCAGTATTTGGCTTTCCAAAGCATTCTTATAAAATTGAAATATCACATTTTTT
TACGTCATAAACCGAAAGGTTTCGTAAGAATATTTTAACTTTATAGTGTAAAAAA
5'UTR
TCTCTTTTTTGGATGTAATTAGGTTGTACTAGTAAAGTATATAAAGTGTAACTTT
AGAGAAAAAACCTACATTAATCCAACATGATCATTTCATATATTTCACATTGAAA
5'UTR
TAGTGAATATATTAAATATCCTTTTTTAAAATGTTAGCAAATCACTAAAGAATTA
ATCACTTATATAATTTATAGGAAAAAATTTTACAATCGTTTAGTGATTTCTTAAT
5'UTR
GAATTTGAATTAAACATTAAAATTGTTATGAAGTTATAATGTTTTATCTCATGTT
CTTAAACTTAATTTGTAATTTTAACAATACTTCAATATTACAAAATAGAGTACAA
5'UTR
ATAAATGCTTTATCTTTTTTGTTAGTTTTTTAAATGTCTAGCATATCTGTAAAGA
TATTTACGAAATAGAAAAAACAATCAAAAAATTTACAGATCGTATAGACATTTCT
5'UTR
TGCCTTAGAAAAAGAATCATGGAGAAGTATGTTAGACTACAGAAGATTGGAGAAG
ACGGAATCTTTTTCTTAGTACCTCTTCATACAATCTGATGTCTTCTAACCTCTTC
Met Glu Lys Tyr Val Arg Leu Gln Lys Ile Gly Glu
5'UTR
NEK1
sgRNA-Antisense (DU57089)
sgRNA-Sense (57081)
D10A nCas9 nick site
D10A nCas9 nick site
GTTCATTTGGAAAAGCCATTCTTGTTAAATCTACAGAAGATGGCAGACAGTATGT
CAAGTAAACCTTTTCGGTAAGAACAATTTAGATGTCTTCTACCGTCTGTCATACA
Gly Ser Phe Gly Lys Ala Ile Leu Val Lys Ser Thr Glu Asp Gly Arg Gln Tyr Val
NEK1
TATCAAGGAAATTAACATCTCAAGAGTAAGTATATTTTTAGTTTGGTTTCATGAG
ATAGTTCCTTTAATTGTAGAGTTCTCATTCATATAAAAATCAAACCAAAGTACTC
Ile Lys Glu Ile Asn Ile Ser Arg
NEK1
Intron
CCAAAGTACTC
Miseq Primer (R)
TAATGGTTTAGTTACAATAGAGCTCATTGTTAGAGATTTTCTTTAAAAAAAAAAA
ATTACCAAATCAATGTTATCTCGAGTAACAATCTCTAAAAGAAATTTTTTTTTTT
NEK1
Intron
ATTACCAAATCAATGTTATCTCG
TCTAGCCTTCTCGTGTGCAGACTTGAGGTCAG
Miseq Primer (R)
C
600
400
D
Bi-allelic knockout
(MiSeq)
5'UTR
Antisense guide
Sense guide
5 bp insertion
5'UTR
Antisense guide
Sense guide
Frame-shift
&
no start codon
