# Supplementary material for: NEK1 autophosphorylation is disrupted by amyotrophic lateral sclerosis-associated missense variants: activity biomarkers and structural insights
Source: Open Biol. 2026 Oct 28;16(10):260209. doi: 10.1098/rsob.260209 (PMC13714390; doi:10.1098/rsob.260209)
Supplement: Supplementary file 1 [file rsob.260209.zip › rsob.260209/rsob260209_si_004.pdf]

TRYPTIC DIGESTION COVERAGE FOR WILD-TYPE GFP-NEK1

FIG S4

Over-expression, HCD; 83%

```
   1 MVSKGEELFT GVVPILVELD GDVNGHKFSV SGEGEGDATY GKLTLKFICT
  51 TGKLPVPWPT LVTTLTYGVQ CFSRYPDHMK QHDFFKSAMP EGYVQERTIF
 101 FKDDGNYKTR AEVKFEGDTL VNRIELKGID FKEDGNILGH KLEYNYNSHN
 151 VYIMADKQKN GIKVNFKIRH NIEDGSVQLA DHYQQNTPIG DGPVLLPDNH
 201 YLSTQSALSK DPNEKRDHMV LLEFVTAAGI TLGMDELYKS GLGSMEKYVR
 251 LQKIGEGSFG KAILVKSTED GRQYVIKEIN ISRMSSKERE ESRREVAVLA
 301 NMKHPNIVQY RESFEENGSL YIVMDYCEGG DLFKRINAQK GVLFQEDQIL
 351 DWFVQICLAL KHVHDRKILH RDIKSQNIFL TKDGTVQLGD FGIARVLNST
 401 VELARTCIGT PYYLSPEICE NKPYNNKSDI WALGCVLYEL CTLKHAFEAG
 451 SMKNLVLKII SGSFPPVSLH YSYDLRSLVS QLFKRNPRDR PSVNSILEKG
 501 FIAKRIEKFL SPQLIAEEFC LKTFSKFGSQ PIPAKRPASG QNSISVMPAQ
 551 KITKPAAKYG IPLAYKKYGD KKLHEKKPLQ KHKQAHQTPE KRVNTGEERR
 601 KISEEAARKR RLEFIEKEKK QKDQIISLMK AEQMKRQEKE RLERINRARE
 651 QGWRNVLSAG GSGEVKAPFL GSGGTIAPSS FSSRGQYEHY HAIFDQMQQQ
 701 RAEDNEAKWK REIYGRGLPE RGILPGVRPG FPYGAAGHHH FPDADDIRKT
 751 LKRLKAVSKQ ANANRQKGQL AVERAKQVEE FLQRKREAMQ NKARAEGHMG
 801 ILQNLAAMYG GRPSSSRGGK PRNKEEEVYL ARLRQIRLQN FNERQQIKAK
 851 LRGEKKEANH SEGQEGSEEA DMRRKKIESL KAHANARAAV LKEQLERKRK
 901 EAYEREKKVW EEHLVAKGVK SSDVSPPLGQ HETGGSPSKQ QMRSVISVTS
 951 ALKEVGVDSS LTDTRETSEE MQKTNNAISS KREILRRLNE NLKAQEDEKG
1001 KQNLSDTFEI NVHEDAKEHE KEKSVSSDRK KWEAGGQLVI PLDELTLDTS
1051 FSTTERHTVG EVIKLGPNGS PRRAWGKSPT DSVLKILGEA ELQLQTELLE
1101 NTTIRSEISP EGEKYKPLIT GEKKVQCISH EINPSAIVDS PVETKSPEFS
1151 EASPQMSLKL EGNLEEPDDL ETEILQEPSG TNKDESLPCT ITDVWISEEK
1201 ETKETQSADR ITIQENEVSE DGVSSTVDQL SDIHIEPGTN DSQHSKCDVD
1251 KSVQPEPFFH KVVHSEHLNL VPQVQSVQCS PEESFAFRSH SHLPPKNKNK
1301 NSLLIGLSTG LFDANNPKML RTCSLPDLSK LFRTLMDVPT VGDVRQDNLE
1351 IDEIEDENIK EGPSDSEDIV FEETDTDLQE LQASMEQLLR EQPGEEYSEE
1401 EESVLKNSDV EPTANGTDVA DEDDNPSSES ALNEEWHSDN SDGEIASECE
1451 CDSVFNHLEE LRLHLEQEMG FEKFFEVYEK IKAIHEDEDE NIEICSKIVQ
1501 NILGNEHQHL YAKILHLVMA DGAYQEDNDE
```

Stable expression, HCD; 74%

```
   1 MVSKGEELFT GVVPILVELD GDVNGHKFSV SGEGEGDATY GKLTLKFICT
  51 TGKLPVPWPT LVTTLTYGVQ CFSRYPDHMK QHDFFKSAMP EGYVQERTIF
 101 FKDDGNYKTR AEVKFEGDTL VNRIELKGID FKEDGNILGH KLEYNYNSHN
 151 VYIMADKQKN GIKVNFKIRH NIEDGSVQLA DHYQQNTPIG DGPVLLPDNH
 201 YLSTQSALSK DPNEKRDHMV LLEFVTAAGI TLGMDELYKS GLGSMEKYVR
 251 LQKIGEGSFG KAILVKSTED GRQYVIKEIN ISRMSSKERE ESRREVAVLA
 301 NMKHPNIVQY RESFEENGSL YIVMDYCEGG DLFKRINAQK GVLFQEDQIL
 351 DWFVQICLAL KHVHDRKILH RDIKSQNIFL TKDGTVQLGD FGIARVLNST
 401 VELARTCIGT PYYLSPEICE NKPYNNKSDI WALGCVLYEL CTLKHAFEAG
 451 SMKNLVLKII SGSFPPVSLH YSYDLRSLVS QLFKRNPRDR PSVNSILEKG
 501 FIAKRIEKFL SPQLIAEEFC LKTFSKFGSQ PIPAKRPASG QNSISVMPAQ
 551 KITKPAAKYG IPLAYKKYGD KKLHEKKPLQ KHKQAHQTPE KRVNTGEERR
 601 KISEEAARKR RLEFIEKEKK QKDQIISLMK AEQMKRQEKE RLERINRARE
 651 QGWRNVLSAG GSGEVKAPFL GSGGTIAPSS FSSRGQYEHY HAIFDQMQQQ
 701 RAEDNEAKWK REIYGRGLPE RGILPGVRPG FPYGAAGHHH FPDADDIRKT
 751 LKRLKAVSKQ ANANRQKGQL AVERAKQVEE FLQRKREAMQ NKARAEGHMG
 801 ILQNLAAMYG GRPSSSRGGK PRNKEEEVYL ARLRQIRLQN FNERQQIKAK
 851 LRGEKKEANH SEGQEGSEEA DMRRKKIESL KAHANARAAV LKEQLERKRK
 901 EAYEREKKVW EEHLVAKGVK SSDVSPPLGQ HETGGSPSKQ QMRSVISVTS
 951 ALKEVGVDSS LTDTRETSEE MQKTNNAISS KREILRRLNE NLKAQEDEKG
1001 KQNLSDTFEI NVHEDAKEHE KEKSVSSDRK KWEAGGQLVI PLDELTLDTS
1051 FSTTERHTVG EVIKLGPNGS PRRAWGKSPT DSVLKILGEA ELQLQTELLE
1101 NTTIRSEISP EGEKYKPLIT GEKKVQCISH EINPSAIVDS PVETKSPEFS
1151 EASPQMSLKL EGNLEEPDDL ETEILQEPSG TNKDESLPCT ITDVWISEEK
1201 ETKETQSADR ITIQENEVSE DGVSSTVDQL SDIHIEPGTN DSQHSKCDVD
1251 KSVQPEPFFH KVVHSEHLNL VPQVQSVQCS PEESFAFRSH SHLPPKNKNK
1301 NSLLIGLSTG LFDANNPKML RTCSLPDLSK LFRTLMDVPT VGDVRQDNLE
1351 IDEIEDENIK EGPSDSEDIV FEETDTDLQE LQASMEQLLR EQPGEEYSEE
1401 EESVLKNSDV EPTANGTDVA DEDDNPSSES ALNEEWHSDN SDGEIASECE
1451 CDSVFNHLEE LRLHLEQEMG FEKFFEVYEK IKAIHEDEDE NIEICSKIVQ
1501 NILGNEHQHL YAKILHLVMA DGAYQEDNDE
```

Over-expression, EThcD; 83%

```
   1 MVSKGEELFT GVVPILVELD GDVNGHKFSV SGEGEGDATY GKLTLKFICT
  51 TGKLPVPWPT LVTTLTYGVQ CFSRYPDHMK QHDFFKSAMP EGYVQERTIF
 101 FKDDGNYKTR AEVKFEGDTL VNRIELKGID FKEDGNILGH KLEYNYNSHN
 151 VYIMADKQKN GIKVNFKIRH NIEDGSVQLA DHYQQNTPIG DGPVLLPDNH
 201 YLSTQSALSK DPNEKRDHMV LLEFVTAAGI TLGMDELYKS GLGSMEKYVR
 251 LQKIGEGSFG KAILVKSTED GRQYVIKEIN ISRMSSKERE ESRREVAVLA
 301 NMKHPNIVQY RESFEENGSL YIVMDYCEGG DLFKRINAQK GVLFQEDQIL
 351 DWFVQICLAL KHVHDRKILH RDIKSQNIFL TKDGTVQLGD FGIARVLNST
 401 VELARTCIGT PYYLSPEICE NKPYNNKSDI WALGCVLYEL CTLKHAFEAG
 451 SMKNLVLKII SGSFPPVSLH YSYDLRSLVS QLFKRNPRDR PSVNSILEKG
 501 FIAKRIEKFL SPQLIAEEFC LKTFSKFGSQ PIPAKRPASG QNSISVMPAQ
 551 KITKPAAKYG IPLAYKKYGD KKLHEKKPLQ KHKQAHQTPE KRVNTGEERR
 601 KISEEAARKR RLEFIEKEKK QKDQIISLMK AEQMKRQEKE RLERINRARE
 651 QGWRNVLSAG GSGEVKAPFL GSGGTIAPSS FSSRGQYEHY HAIFDQMQQQ
 701 RAEDNEAKWK REIYGRGLPE RGILPGVRPG FPYGAAGHHH FPDADDIRKT
 751 LKRLKAVSKQ ANANRQKGQL AVERAKQVEE FLQRKREAMQ NKARAEGHMG
 801 ILQNLAAMYG GRPSSSRGGK PRNKEEEVYL ARLRQIRLQN FNERQQIKAK
 851 LRGEKKEANH SEGQEGSEEA DMRRKKIESL KAHANARAAV LKEQLERKRK
 901 EAYEREKKVW EEHLVAKGVK SSDVSPPLGQ HETGGSPSKQ QMRSVISVTS
 951 ALKEVGVDSS LTDTRETSEE MQKTNNAISS KREILRRLNE NLKAQEDEKG
1001 KQNLSDTFEI NVHEDAKEHE KEKSVSSDRK KWEAGGQLVI PLDELTLDTS
1051 FSTTERHTVG EVIKLGPNGS PRRAWGKSPT DSVLKILGEA ELQLQTELLE
1101 NTTIRSEISP EGEKYKPLIT GEKKVQCISH EINPSAIVDS PVETKSPEFS
1151 EASPQMSLKL EGNLEEPDDL ETEILQEPSG TNKDESLPCT ITDVWISEEK
1201 ETKETQSADR ITIQENEVSE DGVSSTVDQL SDIHIEPGTN DSQHSKCDVD
1251 KSVQPEPFFH KVVHSEHLNL VPQVQSVQCS PEESFAFRSH SHLPPKNKNK
1301 NSLLIGLSTG LFDANNPKML RTCSLPDLSK LFRTLMDVPT VGDVRQDNLE
1351 IDEIEDENIK EGPSDSEDIV FEETDTDLQE LQASMEQLLR EQPGEEYSEE
1401 EESVLKNSDV EPTANGTDVA DEDDNPSSES ALNEEWHSDN SDGEIASECE
1451 CDSVFNHLEE LRLHLEQEMG FEKFFEVYEK IKAIHEDEDE NIEICSKIVQ
1501 NILGNEHQHL YAKILHLVMA DGAYQEDNDE
```

Stable expression, EThcD; 75%

```
   1 MVSKGEELFT GVVPILVELD GDVNGHKFSV SGEGEGDATY GKLTLKFICT
  51 TGKLPVPWPT LVTTLTYGVQ CFSRYPDHMK QHDFFKSAMP EGYVQERTIF
 101 FKDDGNYKTR AEVKFEGDTL VNRIELKGID FKEDGNILGH KLEYNYNSHN
 151 VYIMADKQKN GIKVNFKIRH NIEDGSVQLA DHYQQNTPIG DGPVLLPDNH
 201 YLSTQSALSK DPNEKRDHMV LLEFVTAAGI TLGMDELYKS GLGSMEKYVR
 251 LQKIGEGSFG KAILVKSTED GRQYVIKEIN ISRMSSKERE ESRREVAVLA
 301 NMKHPNIVQY RESFEENGSL YIVMDYCEGG DLFKRINAQK GVLFQEDQIL
 351 DWFVQICLAL KHVHDRKILH RDIKSQNIFL TKDGTVQLGD FGIARVLNST
 401 VELARTCIGT PYYLSPEICE NKPYNNKSDI WALGCVLYEL CTLKHAFEAG
 451 SMKNLVLKII SGSFPPVSLH YSYDLRSLVS QLFKRNPRDR PSVNSILEKG
 501 FIAKRIEKFL SPQLIAEEFC LKTFSKFGSQ PIPAKRPASG QNSISVMPAQ
 551 KITKPAAKYG IPLAYKKYGD KKLHEKKPLQ KHKQAHQTPE KRVNTGEERR
 601 KISEEAARKR RLEFIEKEKK QKDQIISLMK AEQMKRQEKE RLERINRARE
 651 QGWRNVLSAG GSGEVKAPFL GSGGTIAPSS FSSRGQYEHY HAIFDQMQQQ
 701 RAEDNEAKWK REIYGRGLPE RGILPGVRPG FPYGAAGHHH FPDADDIRKT
 751 LKRLKAVSKQ ANANRQKGQL AVERAKQVEE FLQRKREAMQ NKARAEGHMG
 801 ILQNLAAMYG GRPSSSRGGK PRNKEEEVYL ARLRQIRLQN FNERQQIKAK
 851 LRGEKKEANH SEGQEGSEEA DMRRKKIESL KAHANARAAV LKEQLERKRK
 901 EAYEREKKVW EEHLVAKGVK SSDVSPPLGQ HETGGSPSKQ QMRSVISVTS
 951 ALKEVGVDSS LTDTRETSEE MQKTNNAISS KREILRRLNE NLKAQEDEKG
1001 KQNLSDTFEI NVHEDAKEHE KEKSVSSDRK KWEAGGQLVI PLDELTLDTS
1051 FSTTERHTVG EVIKLGPNGS PRRAWGKSPT DSVLKILGEA ELQLQTELLE
1101 NTTIRSEISP EGEKYKPLIT GEKKVQCISH EINPSAIVDS PVETKSPEFS
1151 EASPQMSLKL EGNLEEPDDL ETEILQEPSG TNKDESLPCT ITDVWISEEK
1201 ETKETQSADR ITIQENEVSE DGVSSTVDQL SDIHIEPGTN DSQHSKCDVD
1251 KSVQPEPFFH KVVHSEHLNL VPQVQSVQCS PEESFAFRSH SHLPPKNKNK
1301 NSLLIGLSTG LFDANNPKML RTCSLPDLSK LFRTLMDVPT VGDVRQDNLE
1351 IDEIEDENIK EGPSDSEDIV FEETDTDLQE LQASMEQLLR EQPGEEYSEE
1401 EESVLKNSDV EPTANGTDVA DEDDNPSSES ALNEEWHSDN SDGEIASECE
1451 CDSVFNHLEE LRLHLEQEMG FEKFFEVYEK IKAIHEDEDE NIEICSKIVQ
1501 NILGNEHQHL YAKILHLVMA DGAYQEDNDE
```
