# Supplementary material for: NEK1 autophosphorylation is disrupted by amyotrophic lateral sclerosis-associated missense variants: activity biomarkers and structural insights
Source: Open Biol. 2026 Oct 28;16(10):260209. doi: 10.1098/rsob.260209 (PMC13714390; doi:10.1098/rsob.260209)
Supplement: Supplementary file 1 [file rsob.260209.zip › rsob.260209/rsob260209_si_005.pdf]

FIG S5

A

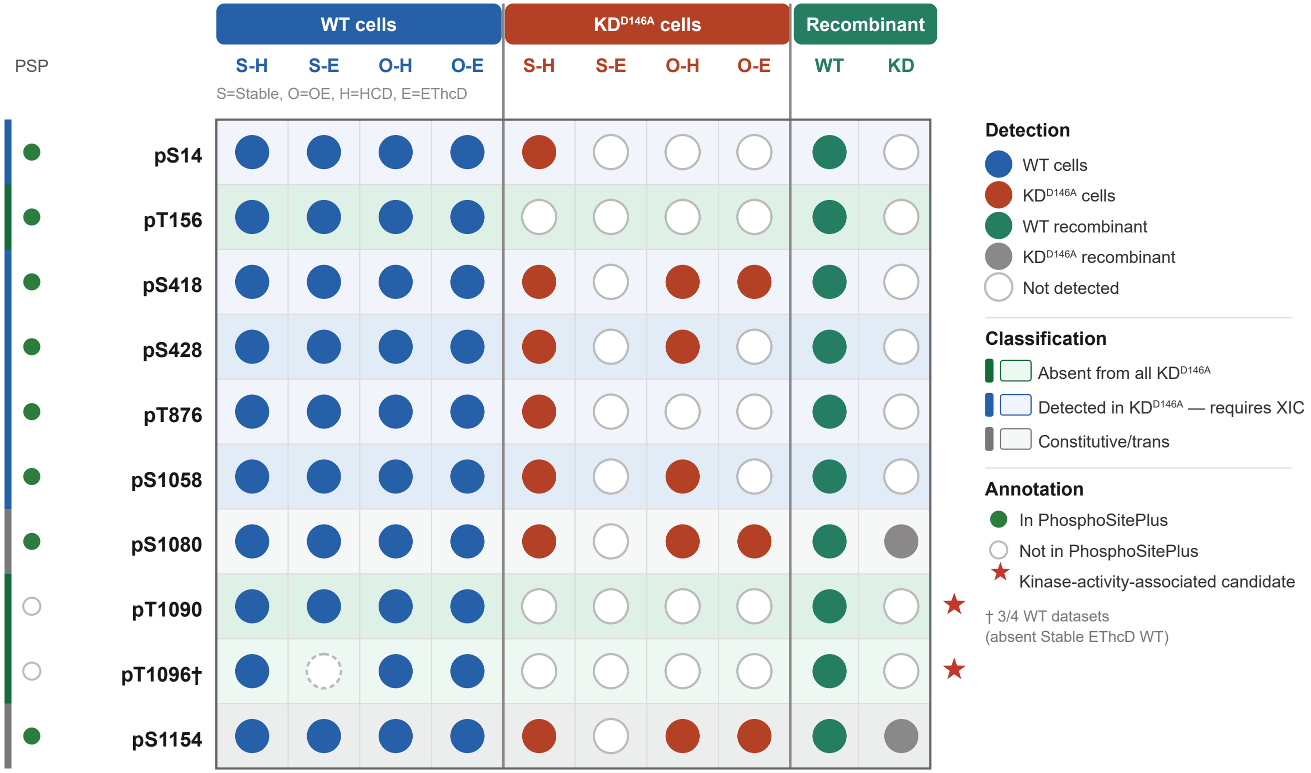

WT cells
KD[D146A] cells
Recombinant
PSP
S-H
S-E
O-H
O-E
WT
KD
S=Stable, O=OE, H=HCD, E=EThcD
pS14
pT156
pS418
pS428
pT876
pS1058
pS1080
pT1090
pT1096†
pS1154
Detection
WT cells
KD[D146A] cells
WT recombinant
KD[D146A] recombinant
Not detected
Classification
Absent from all KD[D146A]
Detected in KD[D146A] — requires XIC
Constitutive/trans
Annotation
In PhosphoSitePlus
Not in PhosphoSitePlus
Kinase-activity-associated candidate
† 3/4 WT datasets
(absent Stable EThcD WT)

B

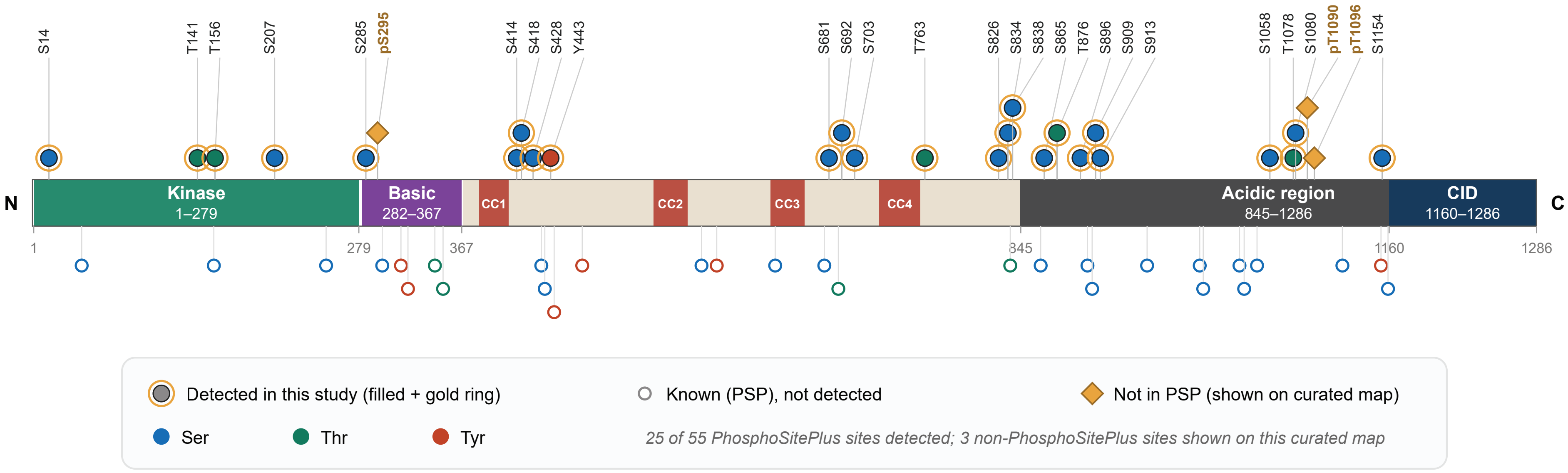

S14
T141
T156
S207
S285
pS295
S414
S418
S428
Y443
S681
S692
S703
T763
S826
S834
S838
S865
T876
S896
S909
S913
S1058
T1078
S1080
pT1090
pT1096
S1154
N
Kinase
1–279
Basic
282–367
CC1
CC2
CC3
CC4
Acidic region
845–1286
CID
1160–1286
C
1
279
367
845
1160
1286
Detected in this study (filled + gold ring)
Known (PSP), not detected
Not in PSP (shown on curated map)
Ser
Thr
Tyr
25 of 55 PhosphoSitePlus sites detected; 3 non-PhosphoSitePlus sites shown on this curated map
