# Supplementary material for: NEK1 autophosphorylation is disrupted by amyotrophic lateral sclerosis-associated missense variants: activity biomarkers and structural insights
Source: Open Biol. 2026 Oct 28;16(10):260209. doi: 10.1098/rsob.260209 (PMC13714390; doi:10.1098/rsob.260209)
Supplement: Supplementary file 1 [file rsob.260209.zip › rsob.260209/rsob260209_si_006.pdf]

FIG S6

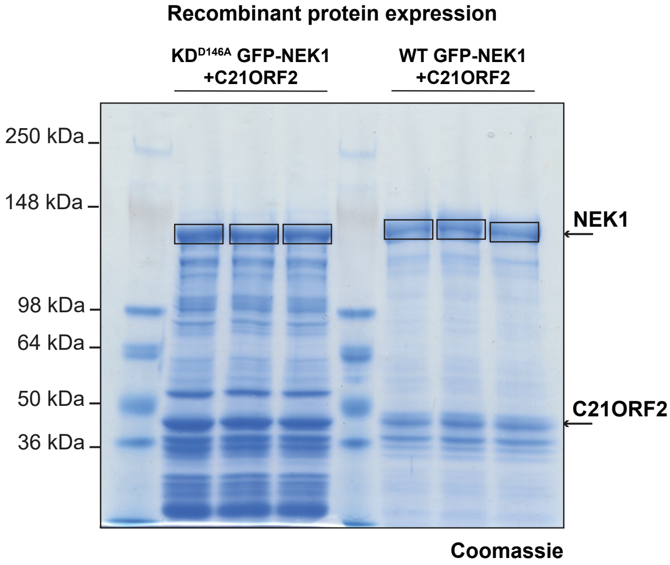
Recombinant protein expression
KDD146A GFP-NEK1 +C21ORF2
WT GFP-NEK1 +C21ORF2
250 kDa
148 kDa
98 kDa
64 kDa
50 kDa
36 kDa
NEK1
C21ORF2
Coomassie
