# Supplementary material for: NEK1 autophosphorylation is disrupted by amyotrophic lateral sclerosis-associated missense variants: activity biomarkers and structural insights
Source: Open Biol. 2026 Oct 28;16(10):260209. doi: 10.1098/rsob.260209 (PMC13714390; doi:10.1098/rsob.260209)
Supplement: Supplementary file 1 [file rsob.260209.zip › rsob.260209/rsob260209_si_007.pdf]

OVEREXPRESSION, WILD-TYPE GFP-NEK1

FIG S7

A

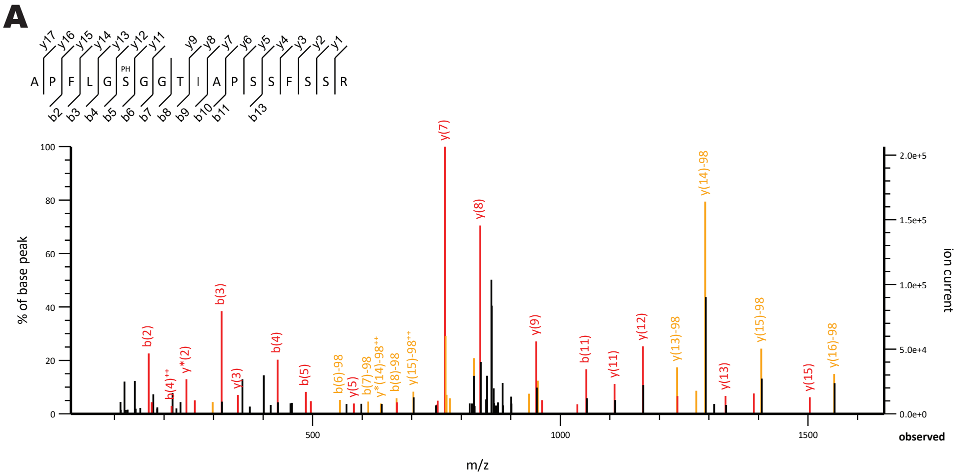

pS428 (HCD; Ion Score 117.6)
Observed m/z: 909.9266 (2+)
Site Analysis: Phospho S6 99.91%
ptmRS: S6 (100%)

B

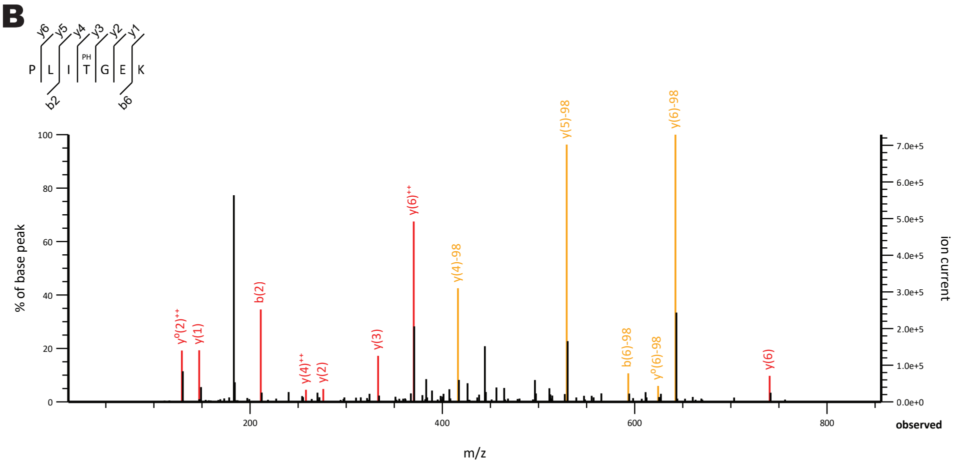

pT876 (HCD; Ion Score 45.5)
Observed m/z: 419.2095 (2+)
ptmRS: T4 (100.0%)

C

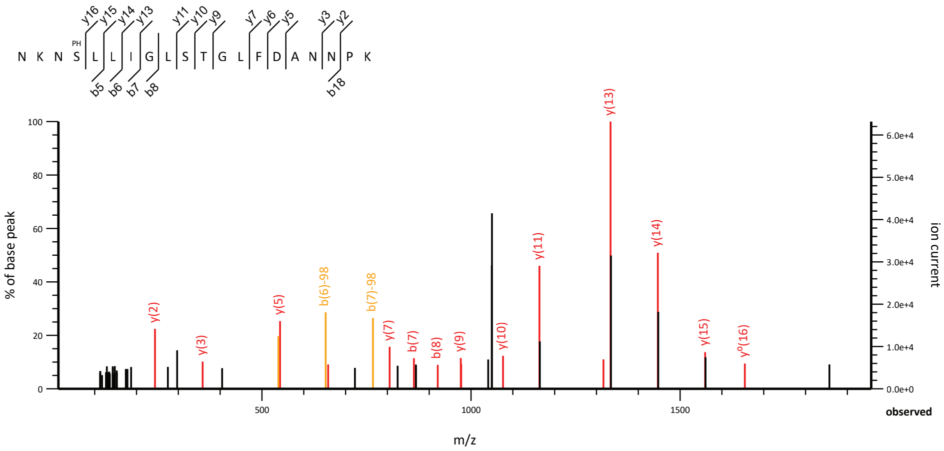

pS1058 (HCD; Ion Score 72.1)
Observed m/z: 1098.5580 (2+)
Site Analysis: Phospho S4 99.99%
ptmRS: S4 (100%)

D

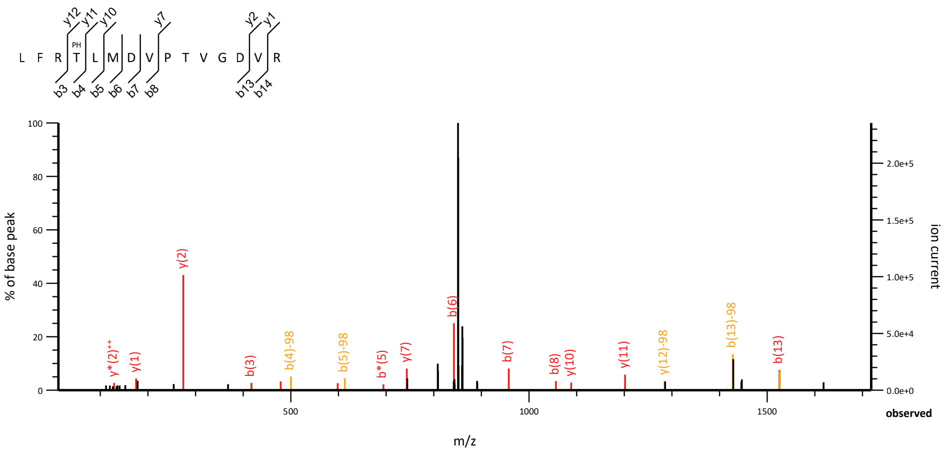

pT1090 (HCD; Ion Score 44.8)
Observed m/z: 899.9522 (2+)
Site Analysis: Phospho T4 99.98%
ptmRS: T4 (100%)
