# Supplementary material for: NEK1 autophosphorylation is disrupted by amyotrophic lateral sclerosis-associated missense variants: activity biomarkers and structural insights
Source: Open Biol. 2026 Oct 28;16(10):260209. doi: 10.1098/rsob.260209 (PMC13714390; doi:10.1098/rsob.260209)

FIG S8

A OVEREXPRESSION, WILD-TYPE GFP-NEK1

pT1090 (EThcD; Ion Score 66.7)
Observed m/z: 600.3016 (3+)
Site Analysis: Phospho T4 100%
ptmRS: T4 (100%)

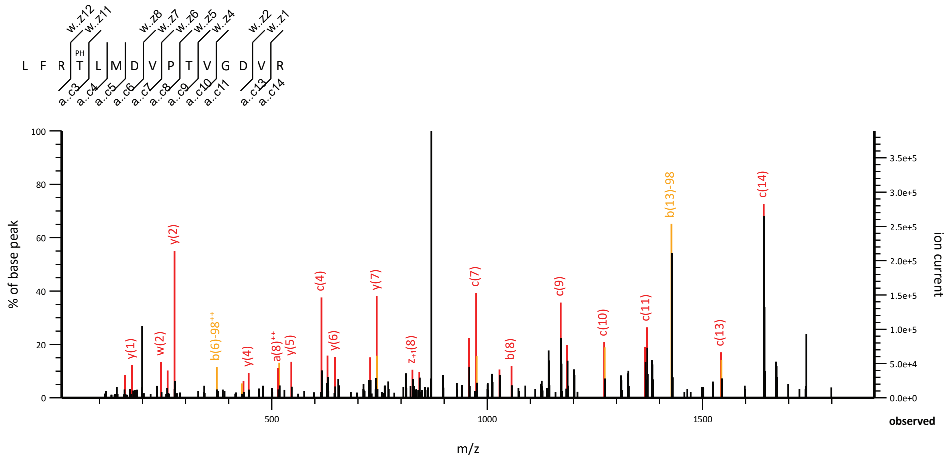

B RECOMBINANT PROTEIN, WILD-TYPE NEK1

pT1090 (HCD; Ion Score 32.2)
Observed m/z: 899.9515 (2+)
Site Analysis: Phospho T4 99.74%
ptmRS: T4 (100%)

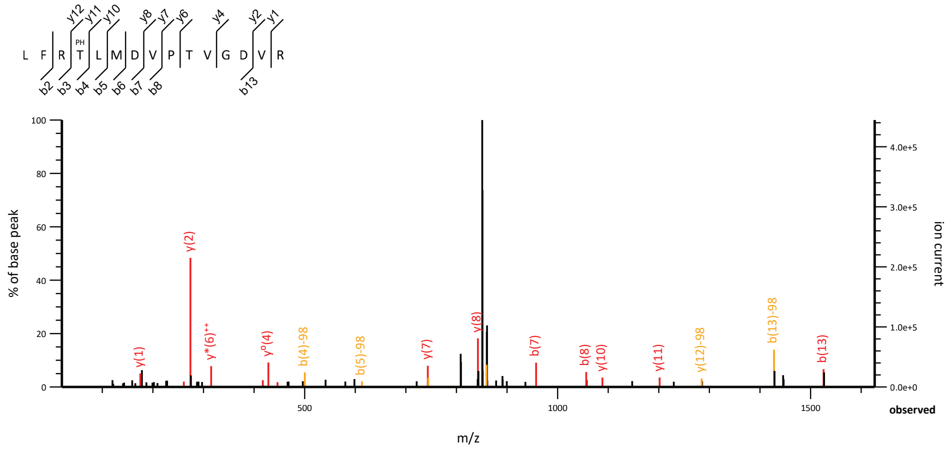

C OVEREXPRESSION, WILD-TYPE GFP-NEK1

pT1096 (HCD; Ion Score 57.6)
Observed m/z: 691.8244 (2+)
Site Analysis: Phospho T7 99.99%
ptmRS: T7 (99.99%)

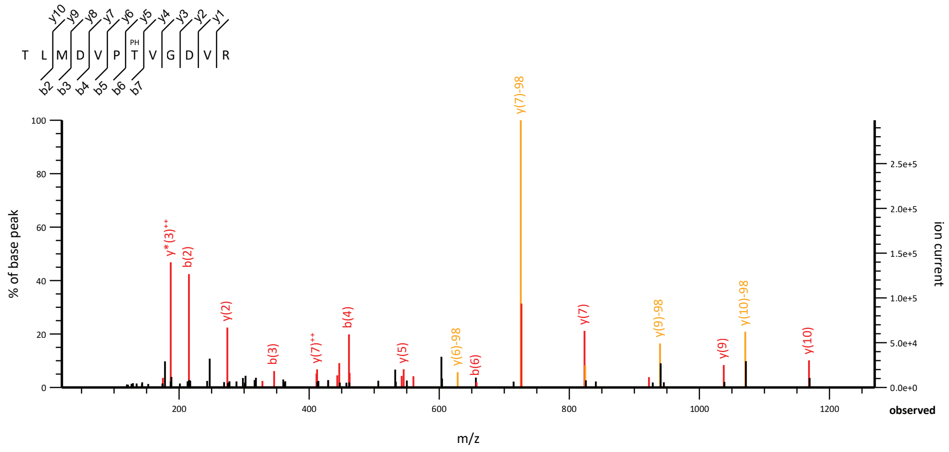

D OVEREXPRESSION, WILD-TYPE GFP-NEK1

pT1096 (EThcD; Ion Score 53.6)
Observed m/z: 691.8231 (2+)
Site Analysis: Phospho T7 100%
ptmRS: T7 (100%)

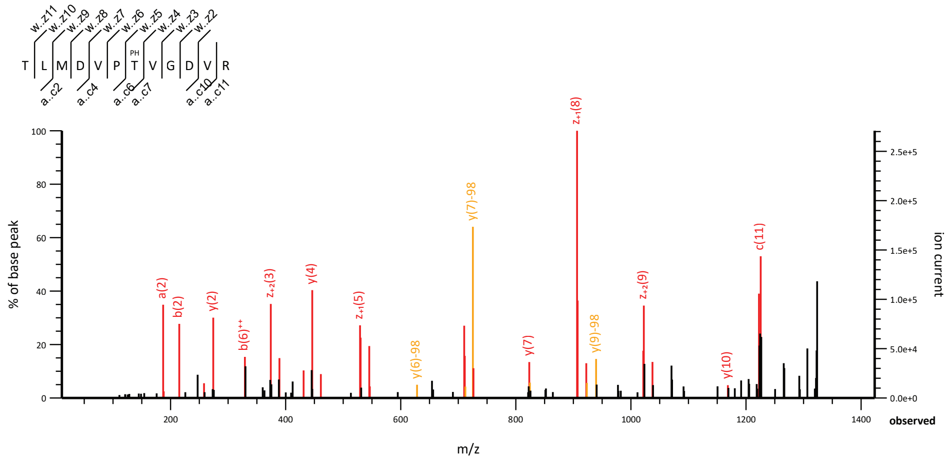

Supplement: Supplementary file 1 [file rsob.260209.zip › rsob.260209/rsob260209_si_008.pdf]
