# Supplementary material for: NEK1 autophosphorylation is disrupted by amyotrophic lateral sclerosis-associated missense variants: activity biomarkers and structural insights
Source: Open Biol. 2026 Oct 28;16(10):260209. doi: 10.1098/rsob.260209 (PMC13714390; doi:10.1098/rsob.260209)

OVEREXPRESSION, WILD-TYPE GFP-NEK1

FIG S9

A

pS14 (HCD; Ion Score 48.2)
Observed m/z: 437.6889
ptmRS: S5 (100%)

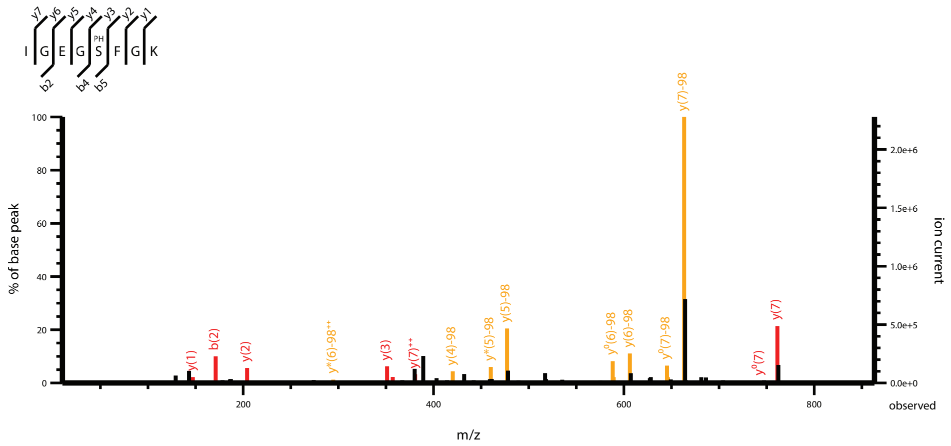

B

pT156 (HCD; Ion Score 63.2)
Observed m/z: 591.3002
Site Analysis: Phospho T5 89.57% (Phospho S4 10.43%)
ptmRS: T5 (99.99%)

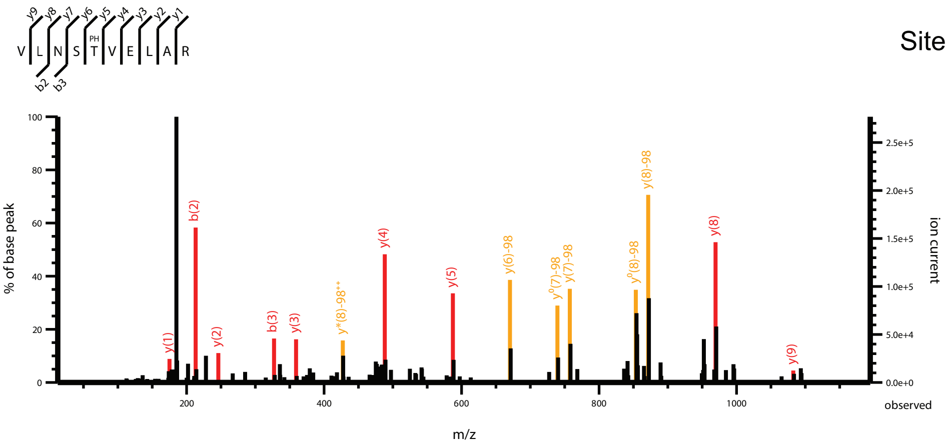

C

pT156 (EThcD; Ion Score 65.2)
Observed m/z: 591.2996
Site Analysis: Phospho T5 95.32% (Phospho S4 4.68%)
ptmRS: T5 (99.96%)

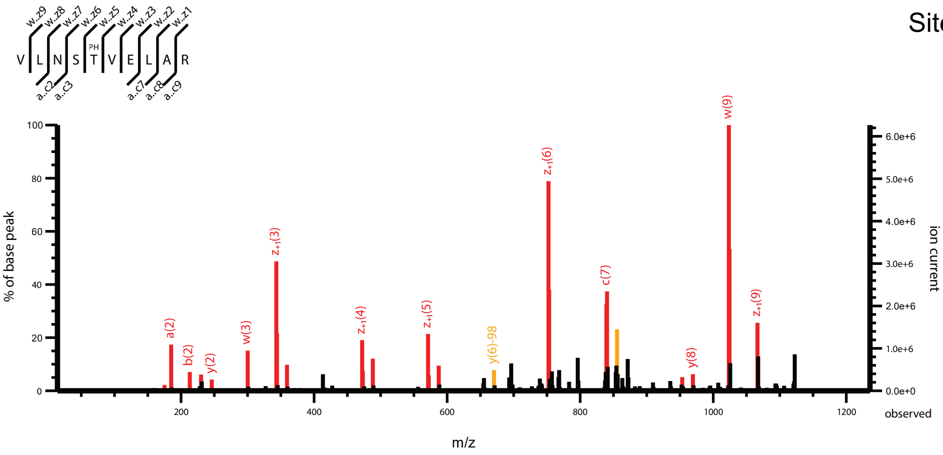

D

pS418 (HCD; Ion Score 60.2)
Observed m/z: 599.2790
Site Analysis: Phospho S8 99.89%
ptmRS: S8 (100%)

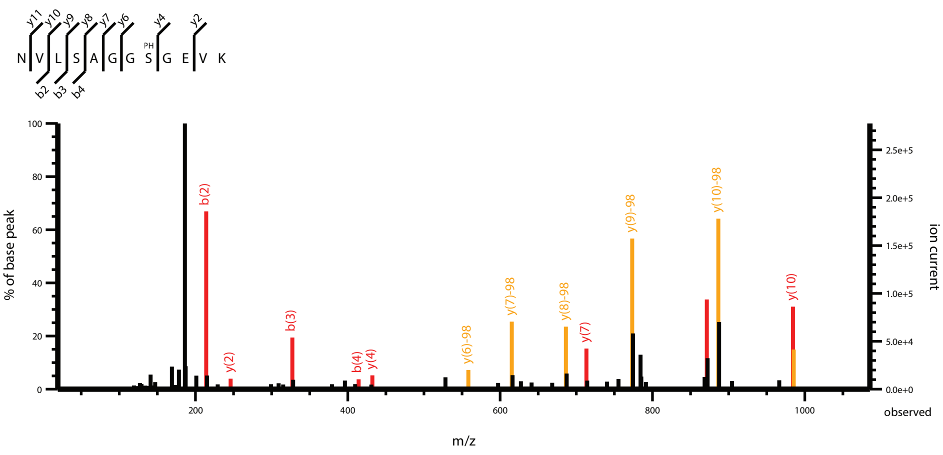

Supplement: Supplementary file 1 [file rsob.260209.zip › rsob.260209/rsob260209_si_009.pdf]
