# Supplementary material for: NEK1 autophosphorylation is disrupted by amyotrophic lateral sclerosis-associated missense variants: activity biomarkers and structural insights
Source: Open Biol. 2026 Oct 28;16(10):260209. doi: 10.1098/rsob.260209 (PMC13714390; doi:10.1098/rsob.260209)

STABLE EXPRESSION, WILD-TYPE GFP-NEK1

FIG S10

A

pS14 (HCD; Ion Score 32.4)
Observed m/z: 437.6895
ptmRS: S5 (100%)

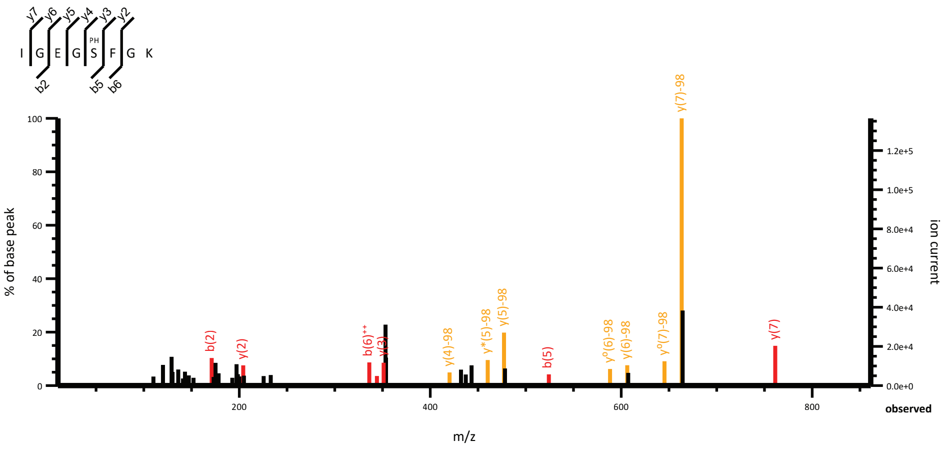

B

pT156 (HCD; Ion Score 51.2)
Observed m/z: 591.3008
Site Analysis: Phospho T5 88.21% (Phospho S4 11.79%)
ptmRS: T5 (100%)

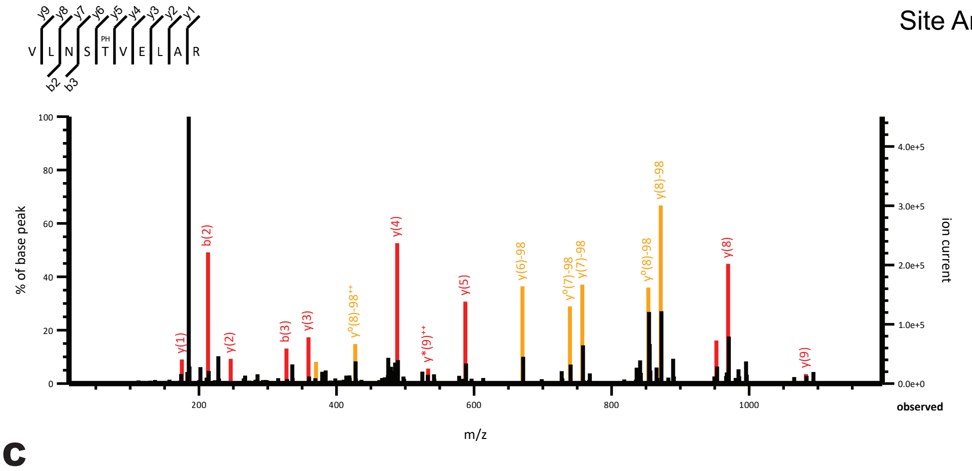

C

pT156 (EThcD; Ion Score 46.2)
Observed m/z: 591.2993
Site Analysis: Phospho T5 94.43% (Phospho S4 5.57%)
ptmRS: T5 (99.04%)

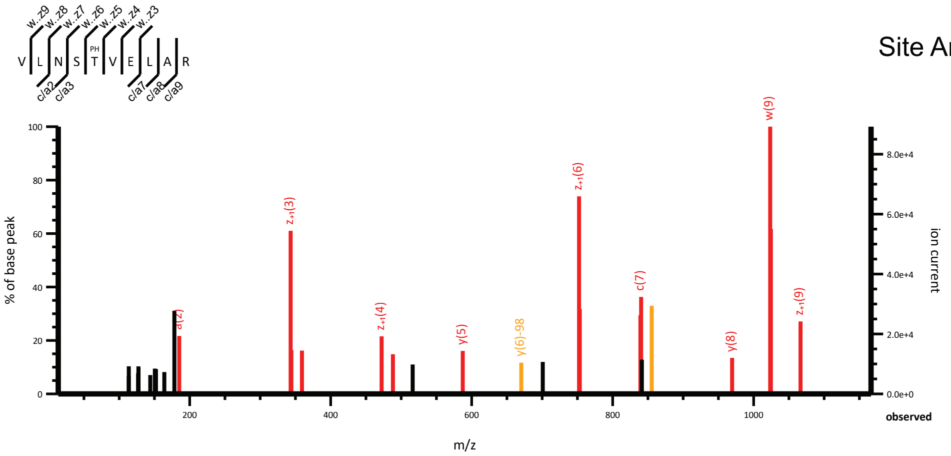

D

pS418 (HCD; Ion Score 43.4)
Observed m/z: 599.2793
Site Analysis: Phospho S8 98.64%
ptmRS: S8 (100%)

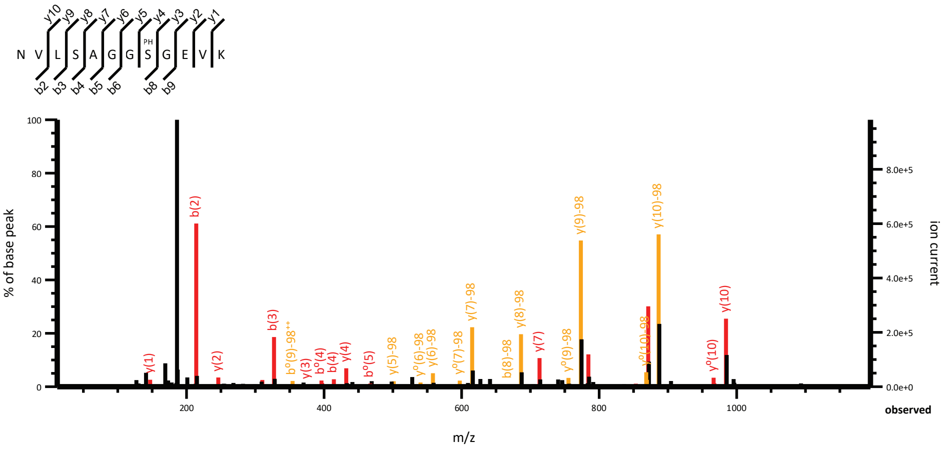

Supplement: Supplementary file 1 [file rsob.260209.zip › rsob.260209/rsob260209_si_010.pdf]
