# Supplementary material for: NEK1 autophosphorylation is disrupted by amyotrophic lateral sclerosis-associated missense variants: activity biomarkers and structural insights
Source: Open Biol. 2026 Oct 28;16(10):260209. doi: 10.1098/rsob.260209 (PMC13714390; doi:10.1098/rsob.260209)
Supplement: Supplementary file 1 [file rsob.260209.zip › rsob.260209/rsob260209_si_011.pdf]

FIG S11

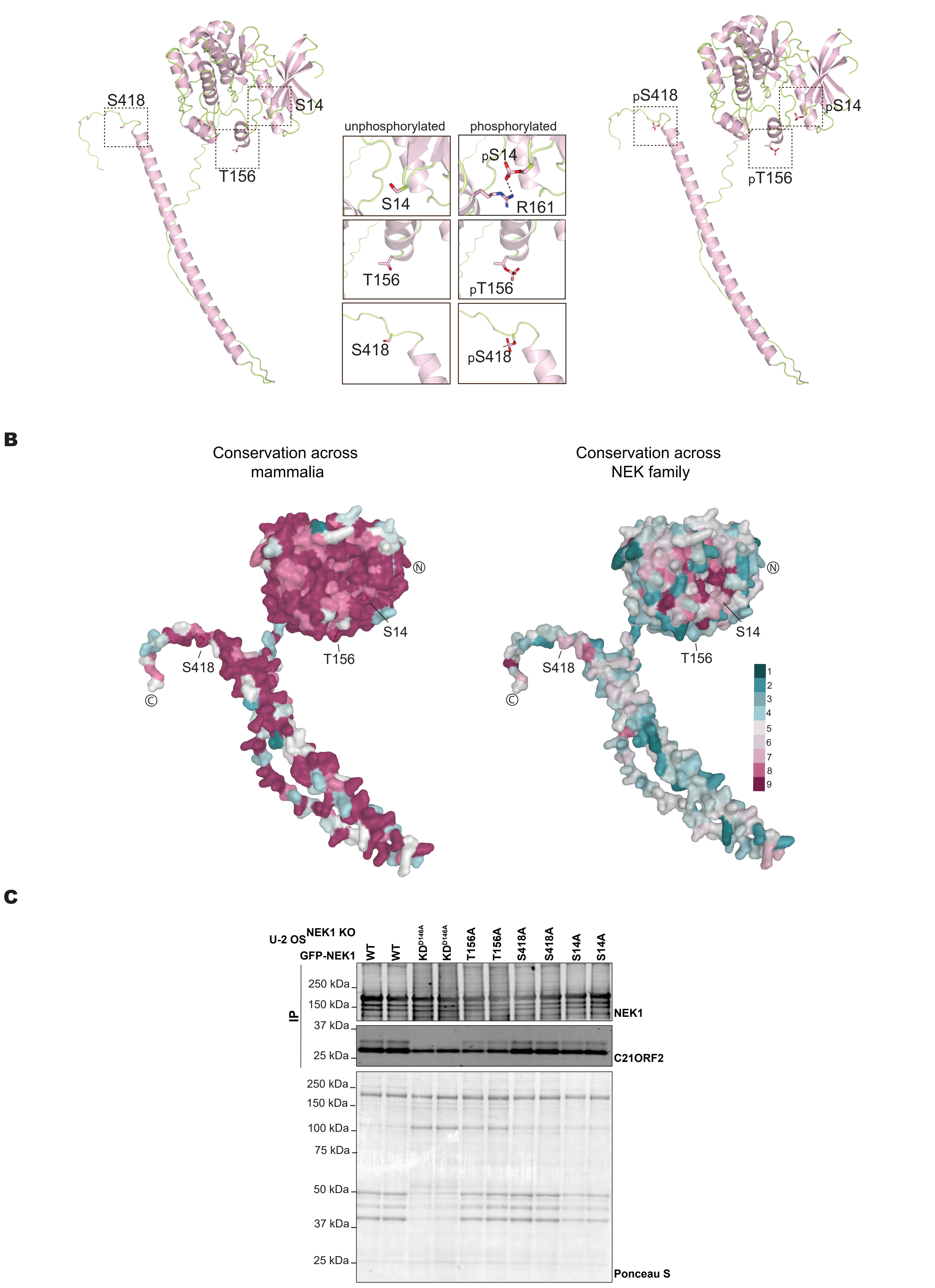
A
S418
S14
T156
unphosphorylated
phosphorylated
S14
pS14
R161
T156
pT156
S418
pS418
pS418
pS14
pT156
B
Conservation across mammalia
Conservation across NEK family
S14
T156
S418
Ⓝ
Ⓒ
1
2
3
4
5
6
7
8
9
C
U-2 OS NEK1 KO
GFP-NEK1
WT
WT
KD D146A
KD D146A
T156A
T156A
S418A
S418A
S14A
S14A
IP
250 kDa
150 kDa
37 kDa
25 kDa
NEK1
C21ORF2
250 kDa
150 kDa
100 kDa
75 kDa
50 kDa
37 kDa
25 kDa
Ponceau S
