# Supplementary material for: NEK1 autophosphorylation is disrupted by amyotrophic lateral sclerosis-associated missense variants: activity biomarkers and structural insights
Source: Open Biol. 2026 Oct 28;16(10):260209. doi: 10.1098/rsob.260209 (PMC13714390; doi:10.1098/rsob.260209)
Supplement: Supplementary file 1 [file rsob.260209.zip › rsob.260209/rsob260209_si_012.pdf]

FIG S12

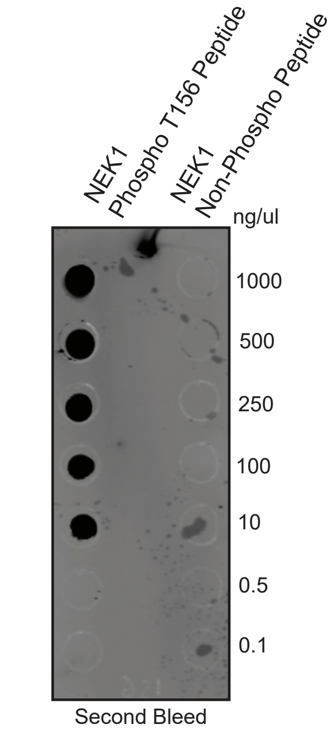

NEK1 pT156 peptide:
IARVLNSpTVELARTC

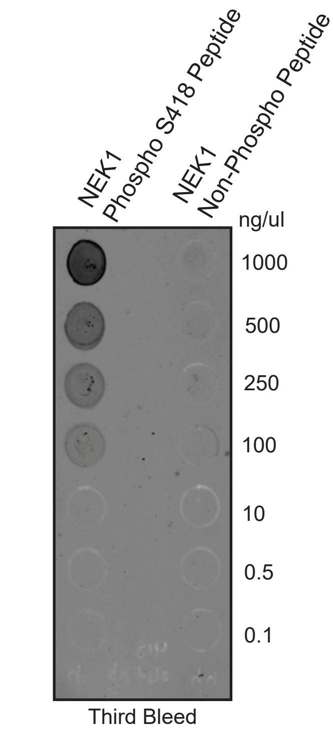

NEK1 pS418 peptide:
NVLSAGGpSGEVKA

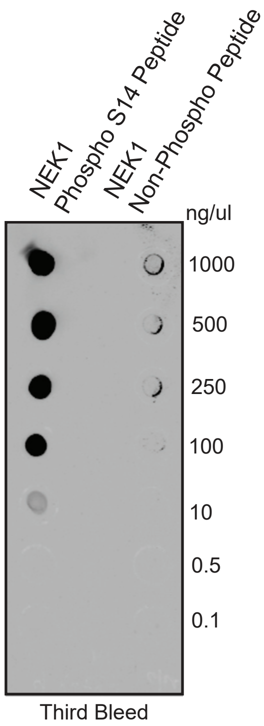

NEK1 pS14 peptide:
QKIGEGpSFGKAILVK

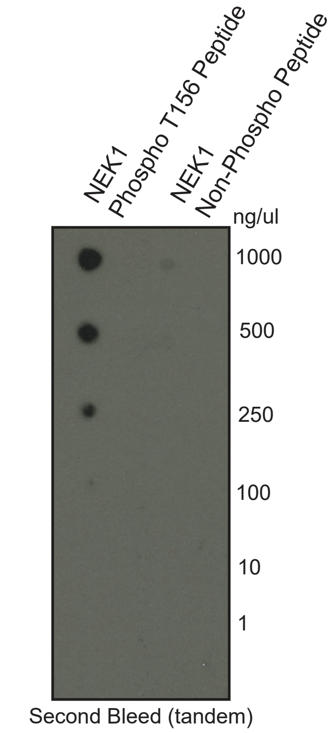

NEK1 pT156 peptide:
IARVLNSpTVELARTC
