# Supplementary material for: NEK1 autophosphorylation is disrupted by amyotrophic lateral sclerosis-associated missense variants: activity biomarkers and structural insights
Source: Open Biol. 2026 Oct 28;16(10):260209. doi: 10.1098/rsob.260209 (PMC13714390; doi:10.1098/rsob.260209)
Supplement: Supplementary file 1 [file rsob.260209.zip › rsob.260209/rsob260209_si_013.pdf]

A

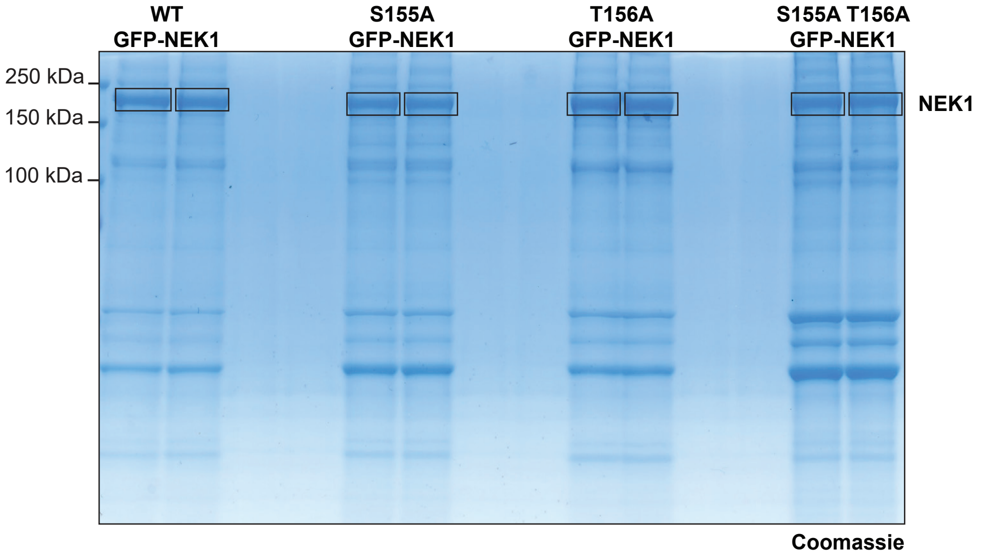
WT
GFP-NEK1
S155A
GFP-NEK1
T156A
GFP-NEK1
S155A T156A
GFP-NEK1
250 kDa
150 kDa
100 kDa
NEK1
Coomassie

B

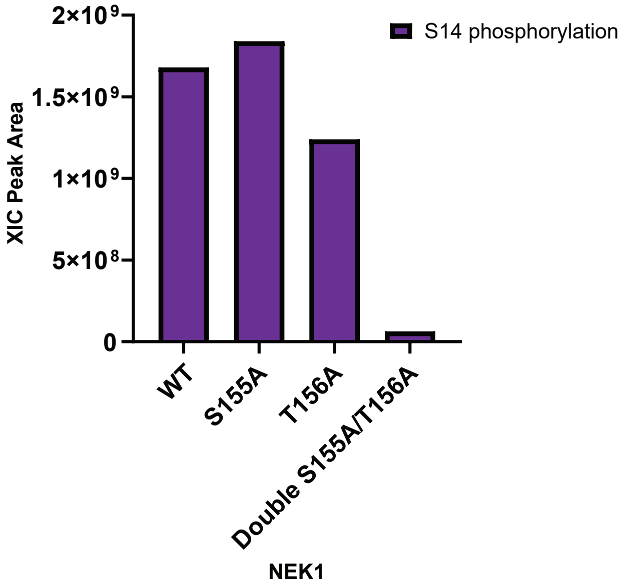
2×10⁹
1.5×10⁹
1×10⁹
5×10⁸
0
XIC Peak Area
S14 phosphorylation
WT
S155A
T156A
Double S155A/T156A
NEK1

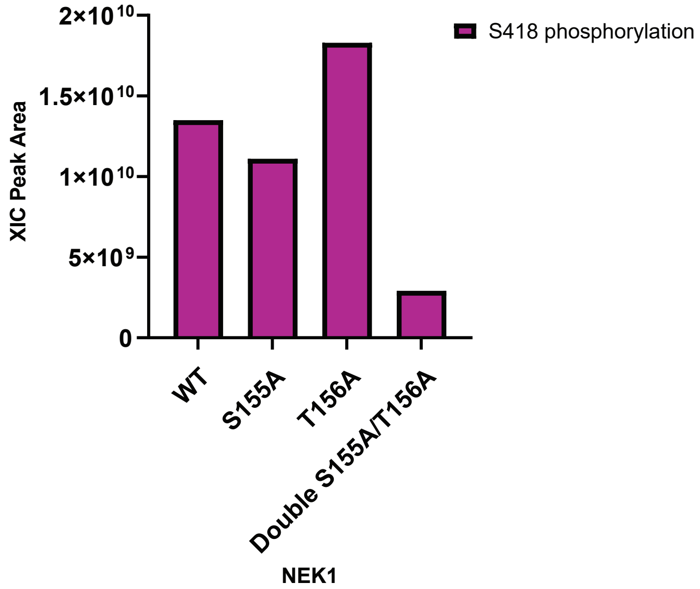
2×10¹⁰
1.5×10¹⁰
1×10¹⁰
5×10⁹
0
XIC Peak Area
S418 phosphorylation
WT
S155A
T156A
Double S155A/T156A
NEK1
