# Supplementary material for: NEK1 autophosphorylation is disrupted by amyotrophic lateral sclerosis-associated missense variants: activity biomarkers and structural insights
Source: Open Biol. 2026 Oct 28;16(10):260209. doi: 10.1098/rsob.260209 (PMC13714390; doi:10.1098/rsob.260209)
Supplement: Supplementary file 1 [file rsob.260209.zip › rsob.260209/rsob260209_si_014.pdf]

FIG S14

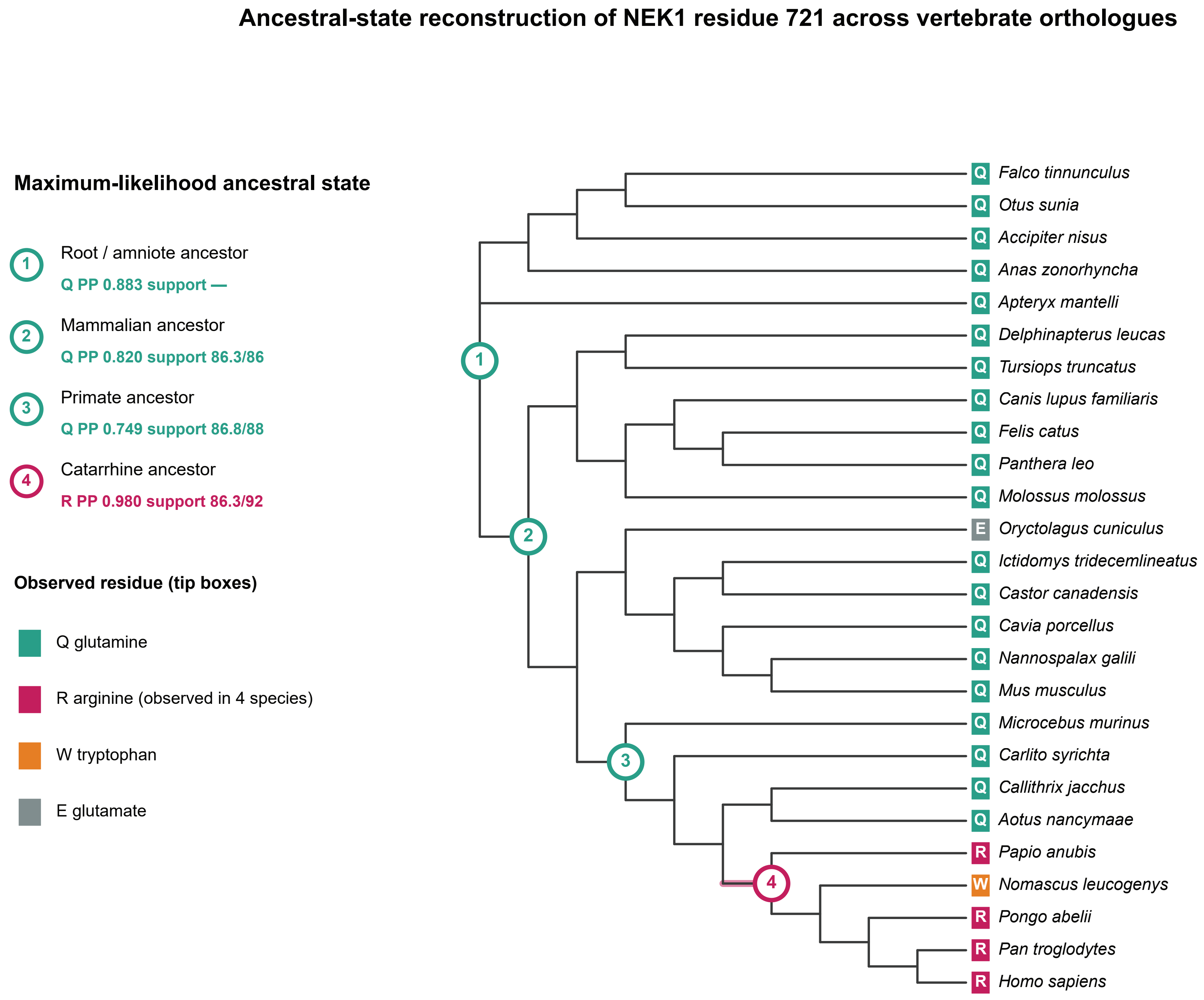
Ancestral-state reconstruction of NEK1 residue 721 across vertebrate orthologues
Maximum-likelihood ancestral state
1
Root / amniote ancestor
Q PP 0.883 support —
2
Mammalian ancestor
Q PP 0.820 support 86.3/86
3
Primate ancestor
Q PP 0.749 support 86.8/88
4
Catarrhine ancestor
R PP 0.980 support 86.3/92
Observed residue (tip boxes)
Q glutamine
R arginine (observed in 4 species)
W tryptophan
E glutamate
Q Falco tinnunculus
Q Otus sunia
Q Accipiter nisus
Q Anas zonorhyncha
Q Apteryx mantelli
Q Delphinapterus leucas
Q Tursiops truncatus
Q Canis lupus familiaris
Q Felis catus
Q Panthera leo
Q Molossus molossus
E Oryctolagus cuniculus
Q Ictidomys tridecemlineatus
Q Castor canadensis
Q Cavia porcellus
Q Nannospalax galili
Q Mus musculus
Q Microcebus murinus
Q Carlito syrichta
Q Callithrix jacchus
Q Aotus nancymaae
R Papio anubis
W Nomascus leucogenys
R Pongo abelii
R Pan troglodytes
R Homo sapiens
