# Supplementary material for: NEK1 autophosphorylation is disrupted by amyotrophic lateral sclerosis-associated missense variants: activity biomarkers and structural insights
Source: Open Biol. 2026 Oct 28;16(10):260209. doi: 10.1098/rsob.260209 (PMC13714390; doi:10.1098/rsob.260209)
Supplement: Supplementary file 1 [file rsob.260209.zip › rsob.260209/rsob260209_si_015.pdf]

FIG S15

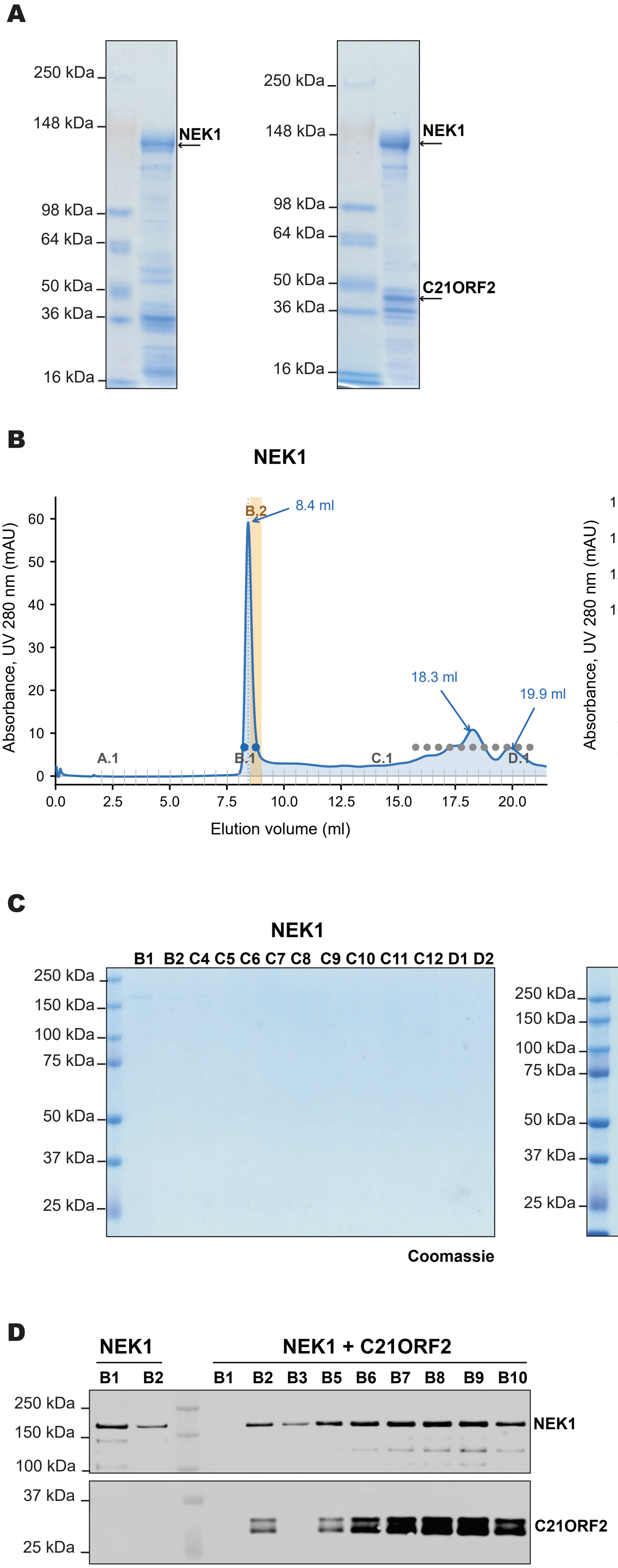
A
250 kDa
148 kDa
98 kDa
64 kDa
50 kDa
36 kDa
16 kDa
NEK1
C21ORF2
B
NEK1
NEK1 + C21ORF2
Absorbance, UV 280 nm (mAU)
Elution volume (ml)
8.4 ml
18.3 ml
19.9 ml
8.7 ml
12.3 ml
B.2
B.9
A.1
B.1
C.1
D.1
C
NEK1
B1 B2 C4 C5 C6 C7 C8 C9 C10 C11 C12 D1 D2
NEK1 + C21ORF2
B1 B2 B3 B5 B6 B7 B8 B9 B10 B11 C5 C6 C7 C8 C9 C10 C11
250 kDa
150 kDa
100 kDa
75 kDa
50 kDa
37 kDa
25 kDa
Coomassie
D
NEK1
NEK1 + C21ORF2
B1 B2
B1 B2 B3 B5 B6 B7 B8 B9 B10
250 kDa
150 kDa
100 kDa
37 kDa
25 kDa
NEK1
C21ORF2
