# Supplementary material for: NEK1 autophosphorylation is disrupted by amyotrophic lateral sclerosis-associated missense variants: activity biomarkers and structural insights
Source: Open Biol. 2026 Oct 28;16(10):260209. doi: 10.1098/rsob.260209 (PMC13714390; doi:10.1098/rsob.260209)
Supplement: Supplementary file 1 [file rsob.260209.zip › rsob.260209/rsob260209_si_016.pdf]

FIG S16

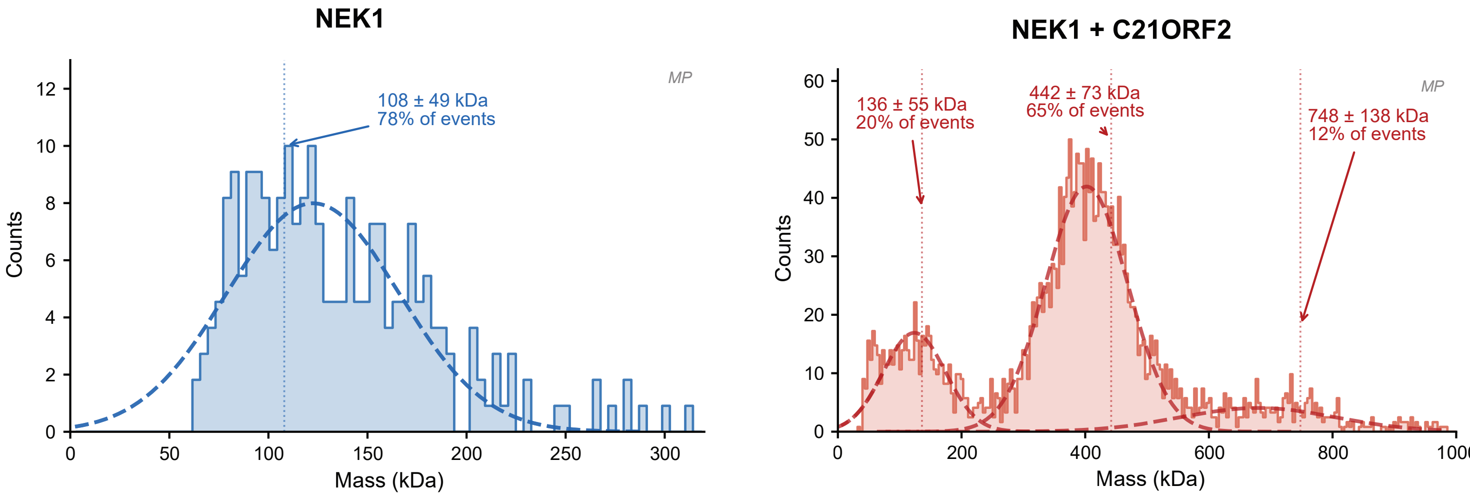

NEK1
108 ± 49 kDa
78% of events
MP
Counts
Mass (kDa)
NEK1 + C21ORF2
136 ± 55 kDa
20% of events
442 ± 73 kDa
65% of events
748 ± 138 kDa
12% of events
MP
Counts
Mass (kDa)
