# Supplementary material for: NEK1 autophosphorylation is disrupted by amyotrophic lateral sclerosis-associated missense variants: activity biomarkers and structural insights
Source: Open Biol. 2026 Oct 28;16(10):260209. doi: 10.1098/rsob.260209 (PMC13714390; doi:10.1098/rsob.260209)
Supplement: Supplementary file 1 [file rsob.260209.zip › rsob.260209/rsob260209_si_023.pdf]

NEK1 autophosphorylation is disrupted by amyotrophic lateral sclerosis-associated missense variants: activity biomarkers and structural insights

Open Biology | Manuscript RSOB-260209 | DOI 10.1098/rsob.260209

Corresponding Author: Arpan R. Mehta – amehta001@dundee.ac.uk

Contents

Supplementary figures

Figure S1: Extended rare variant association statistics and structural mapping of ALS-associated *NEK1* missense variants.

Figure S2: Evolutionary conservation of NEK1 across 26 vertebrate species.

Figure S3: CRISPR-Cas9 nickase-mediated generation and validation of a *NEK1* homozygous knockout U-2 OS cell line.

Figure S4: Tryptic digestion sequence coverage of wild-type GFP-NEK1.

Figure S5: Detection of NEK1 phosphorylation sites across all experimental datasets.

Figure S6: Recombinant expression and purification of NEK1 and the NEK1–C21ORF2 complex.

Figure S7: Mascot-derived annotated peptide-spectrum match reports supporting phosphosite localisation for pSer428, pThr876, pSer1058 and pThr1090 in wild-type GFP-NEK1 following transient overexpression.

Figure S8: Mascot-derived annotated peptide-spectrum match reports supporting phosphosite localisation for pThr1090, detected by EThcD and recombinant HCD, and pThr1096, detected by HCD and EThcD, in wild-type cell-based GFP-NEK1 and recombinant NEK1 datasets.

Figure S9: Mascot-derived annotated peptide-spectrum match reports supporting phosphosite localisation for pSer14, pThr156 and pSer418 in wild-type GFP-NEK1—transient overexpression.

Figure S10: Mascot-derived annotated peptide-spectrum match reports supporting phosphosite localisation for pSer14, pThr156 and pSer418 in wild-type GFP-NEK1—stable expression.

Figure S11: Structural context and conservation of NEK1 autophosphorylation sites, and C21ORF2 binding by phosphosite mutants.

Figure S12: Dot blot validation of phosphospecific antibodies against NEK1 pSer14, pThr156 and pSer418.

Figure S13: Activation-loop mutagenesis reveals coupling between the Ser155/Thr156 region and distal NEK1 autophosphorylation sites.

Figure S14: Ancestral-state reconstruction of NEK1 residue 721 across vertebrate orthologues.

Figure S15: Size-exclusion chromatography of NEK1 and the NEK1–C21ORF2 complex.

Figure S16: Mass photometry of NEK1 and the NEK1–C21ORF2 complex.

Supplementary tables

Table S1: Cell-based phosphoproteomic map of wild-type NEK1.

Table S2: Cell-based phosphoproteomic map of kinase-dead NEK1.

Table S3: Recombinant NEK1 phosphoproteomic map (wild-type and kinase-dead).

Table S4: Source data for XIC quantification of NEK1 phosphosites (Figure 3B).

Table S5: Key resources and reagents.

Table S6: Single-variant and gene-burden association statistics for *NEK1* in ALS.

Electronic supplementary material figure legends

Figure S1: Extended rare variant association statistics and structural mapping of ALS-associated *NEK1* missense variants. (**A**) Lollipop plot of association statistics for rare *NEK1* variants of moderate or high predicted impact, plotted by coding DNA sequence position (ENST00000507142.6). As in Figure 1B, the plot is restricted to variants passing the call-rate quality-control filters (see Methods) and with a minor allele frequency below 0.05. Each point represents one variant; point size reflects |Log(OR)|, shape indicates predicted mutational impact (triangle: HIGH; circle: MODERATE), and fill colour indicates –log10(P) on a scale truncated at 4, chosen so that variation amongst the majority of variants is resolvable. Variants exceeding the plotted y-axis range are shown above an axis break and annotated with their exact P value. Variants drawn in red, in place of the –log10(P) colour scale, are the nine missense variants selected for functional characterisation in this study, the same nine shown in Figure 1B; note that further variants of high predicted impact also reach $P < 0.05$ but were not selected. These nine are labelled by amino acid substitution, with leader lines indicating the corresponding data points. Yellow shading demarcates individual exons. Variants orientated below the baseline are less frequent in ALS cases than in matched controls. MODERATE impact variants comprise missense and inframe deletion variants, and HIGH impact variants comprise nonsense and frameshift mutations, all predicted using SnpEff, together with splice acceptor/donor variants predicted using dbscSNV. (**B**) AlphaFold3 structural model of full-length NEK1 with all ALS-associated missense variants selected for functional characterisation indicated at their

respective positions (V223M, R232C/H, R261C/H, A313T, R440Q, K648E, R721Q). D146, which when mutated to alanine renders NEK1 kinase-dead, is also indicated as a structural reference point.

Figure S2: Evolutionary conservation of NEK1 across 26 vertebrate species. Multiple sequence alignment of NEK1 protein sequences from 26 vertebrate species, generated using MAFFT with default parameters. Per-residue evolutionary conservation scores were calculated using ConSurf with human NEK1 isoform 3 (UniProtKB Q96PY6-3) as the query, and are displayed using the standard nine-colour ConSurf palette (1, most variable; 9, most conserved). Residues in the human sequence corresponding to alignment gaps are uncoloured.

Figure S3: CRISPR-Cas9 nickase-mediated generation and validation of a *NEK1* homozygous knockout U-2 OS cell line. (**A**) Schematic of the CRISPR-Cas9 nickase targeting strategy. Exon 3 was selected as the target because it is the first coding exon shared by all *NEK1* protein-coding isoforms, ensuring complete knockout of all expressed variants. A paired nickase approach using D10A Cas9 was employed, with sense (DU57081) and antisense (DU57089) guide RNAs positioned to nick opposite strands in close proximity to the ATG start codon. (**B**) Annotated sequence map of the *NEK1* exon 3 locus, showing the positions of the sense and antisense sgRNAs, D10A nCas9 nick sites, MiSeq primer binding sites (forward and reverse), the 5′UTR, and the beginning of the *NEK1* coding sequence with translated amino acids indicated. (**C**) 1% Agarose gel showing the 481 bp wild-type PCR amplicon (arrow) spanning the CRISPR target site, used as input for amplicon-based next-generation sequencing. (**D**) MiSeq sequencing confirmation of biallelic *NEK1* gene disruption. Both alleles harbour distinct frameshift-inducing indels: one allele carries a 5 bp insertion introducing a premature stop codon, and the second allele carries a frameshift mutation that abolishes the start codon, confirming complete loss of NEK1 protein expression from both alleles.

Figure S4: Tryptic digestion sequence coverage of wild-type GFP-NEK1. Sequence coverage maps for wild-type GFP-NEK1 following Trypsin/Lys-C in-gel digestion and LC-MS/MS analysis, shown as a representative example of coverage achieved across all samples. Data are shown for transient overexpression and stable expression conditions using HCD and EThcD fragmentation (83%, 83%, 74%, and 75% coverage respectively). Detected peptides are highlighted in red; undetected regions are shown in black. In these maps EGFP occupies construct residues 1–239, the Ser-Gly-Leu-Gly-Ser linker residues 240–244 and NEK1 isoform 3 residues 245–1530; NEK1 isoform 3 numbering is obtained by subtracting 244.

Figure S5: Detection of NEK1 phosphorylation sites across all experimental datasets. (**A**) Matrix summarising detection across the cell-based acquisitions—WT and KD^{D146A}

NEK1, each in the stable and overexpression systems by HCD and EThcD—together with the recombinant WT and KD^{D146A} datasets. Rows (isoform 3 coordinates) comprise the eight kinase-activity-associated candidate phosphosites carried forward for XIC quantification (pSer14, pThr156, pSer418, pSer428, pThr876, pSer1058, pThr1090 and pThr1096), together with the two recurrent sites detected in recombinant KD^{D146A} (pSer1080 and pSer1154; classified as constitutive/trans-phosphorylated). Filled symbols denote detection and open symbols non-detection in each dataset; the left-hand column indicates annotation in PhosphoSitePlus® (filled, annotated; open, not annotated). Colour banding classifies each site as absent from kinase-dead datasets, detected in KD^{D146A} datasets and therefore requiring XIC quantification, or constitutive/trans-phosphorylated. Sites prioritised as kinase-activity-associated candidates (pThr1090 and pThr1096) are starred; pThr1096 was detected in three of the four WT cellular datasets (absent from the Stable EThcD dataset). (**B**) All 55 PhosphoSitePlus®-annotated phosphorylation sites mapped onto the NEK1 isoform 3 domain architecture (UniProtKB Q96PY6-3; 1,286 aa). Sites detected in this study are shown above the protein bar as filled circles with a gold ring ($n = 25$); known PhosphoSitePlus® sites not detected are shown below as open circles ($n = 30$). Three selected non-PhosphoSitePlus® sites are indicated by gold diamonds on this curated map: pSer295, pThr1090 and pThr1096. Additional non-PhosphoSitePlus® detections present in subset datasets are listed in Table S1. Residue type is colour-coded (blue, Ser; green, Thr; red, Tyr). Dark red segments within the linker region denote the four predicted coiled-coil domains (CC1, 382–407; CC2, 531–560; CC3, 631–660; CC4, 724–759).

Figure S6: Recombinant expression and purification of NEK1 and the NEK1–C21ORF2 complex. Coomassie Brilliant Blue-stained SDS-PAGE of WT and KD^{D146A} NEK1, co-expressed with C21ORF2 in Sf9 insect cells as GFP-tagged constructs, purified by anti-GFP affinity chromatography and recovered by RV-3C cleavage before SDS-PAGE (three independent preparations per construct). Bands corresponding to NEK1 (boxed) and C21ORF2 are indicated; the NEK1 bands were excised for in-gel digestion and LC-MS/MS analysis. Molecular-weight markers (kDa) are shown at left.

Figure S7: Mascot-derived annotated peptide-spectrum match reports supporting phosphosite localisation for pSer428, pThr876, pSer1058 and pThr1090 in wild-type GFP-NEK1 following transient overexpression. Representative Mascot-derived annotated HCD MS/MS spectra from transient overexpression of wild-type GFP-NEK1 in *NEK1* knockout U-2 OS cells. Sequence ladders above each spectrum indicate matched backbone cleavages, with the phosphorylated residue marked PH. Sequence ions (b- and y-series) are shown in red; phosphorylation-directed neutral loss ions ($-H_3PO_4$, −98 Da) are shown in orange; unassigned peaks are shown in black. Positional annotations refer to residue numbering within the tryptic peptide; NEK1 isoform 3 site identities are given in parentheses. (**A**) pSer428, identified by HCD fragmentation of tryptic peptide

APFLGSGGTIAPSSFSSR (Ion Score 117.6; observed m/z 909.9266, $[M+2H]^{2+}$; Mascot site analysis: Phospho S6 99.91%; ptmRS: S6 100%). (**B**) pThr876, identified by HCD fragmentation of tryptic peptide PLITGEK (Ion Score 45.5; observed m/z 419.2095, $[M+2H]^{2+}$; ptmRS: T4 100.0%). Thr4 is the sole phosphorylatable residue within this peptide; site localisation is therefore unambiguous by definition. The prominent unlabelled peak at m/z 183.15 corresponds to the a(2) ion (PL; b2 – CO), a characteristic N-terminal fragment not annotated by default in Mascot. (**C**) pSer1058, identified by HCD fragmentation of tryptic peptide NKNSLLIGLSTGLFDANNPK (Ion Score 72.1; observed m/z 1098.5580, $[M+2H]^{2+}$; Mascot site analysis: Phospho S4 99.99%; ptmRS: S4 100%). (**D**) pThr1090, identified by HCD fragmentation of tryptic peptide LFRTLMDVPTVGDVR (Ion Score 44.8; observed m/z 899.9522, $[M+2H]^{2+}$; Mascot site analysis: Phospho T4 99.98%; ptmRS: T4 100%). The dominant unlabelled peak at m/z 899.95 corresponds to the unfragmented doubly-charged precursor ion.

Figure S8: Mascot-derived annotated peptide-spectrum match reports supporting phosphosite localisation for pThr1090, detected by EThcD and recombinant HCD, and pThr1096, detected by HCD and EThcD, in wild-type cell-based GFP-NEK1 and recombinant NEK1 datasets. Annotated Mascot-derived MS/MS spectra confirming phosphosite localisation for pThr1090 and pThr1096. (**A, D**) EThcD panels: c-ions and z-radical ions arising from the electron-transfer component are shown in red alongside HCD-derived b/y-ions; sequence ladder tick marks denote matched a/c-ions (below sequence) and w/z-ions (above sequence) as annotated by Mascot for EThcD data. (**B, C**) HCD panels: sequence ions (b- and y-series) are shown in red; phosphorylation-directed neutral loss ions ($-H_3PO_4$, –98 Da) are shown in orange. Unassigned peaks are shown in black throughout. Positional annotations refer to residue numbering within the tryptic peptide. (**A**) pThr1090, confirmed by EThcD fragmentation of tryptic peptide LFRTLMDVPTVGDVR from transient overexpression of WT GFP-NEK1 in *NEK1* knockout U-2 OS cells (Ion Score 66.7; observed m/z 600.3016, $[M+3H]^{3+}$; Mascot site analysis: Phospho T4 100%; ptmRS: T4 100%). The dominant unlabelled peak at m/z ~900 corresponds to the charge-reduced precursor ($[M+2H]^{2+\bullet}$), a characteristic artefact of electron-transfer fragmentation. (**B**) pThr1090, confirmed by HCD fragmentation of tryptic peptide LFRTLMDVPTVGDVR from recombinant WT NEK1 purified from Sf9 insect cells after GFP-affinity purification and RV-3C tag cleavage (Ion Score 32.2; observed m/z 899.9515, $[M+2H]^{2+}$; Mascot site analysis: Phospho T4 99.74%; ptmRS: T4 100%). This spectrum provides independent, cell-free support for pThr1090 detection in WT recombinant NEK1. (**C**) pThr1096, identified by HCD fragmentation of tryptic peptide TLMDVPTVGDVR from transient overexpression of WT GFP-NEK1 in *NEK1* knockout U-2 OS cells (Ion Score 57.6; observed m/z 691.8244, $[M+2H]^{2+}$; Mascot site analysis: Phospho T7 99.99%; ptmRS: T7 99.99%). Note that this peptide spans both Thr1090 (T1) and Thr1096 (T7); the spectrum unambiguously localises phosphorylation to Thr1096 (T7, 99.99%), with Thr1090

unphosphorylated in this PSM. (**D**) pThr1096, confirmed by EThcD fragmentation of tryptic peptide TLMDVPTVGDVR from transient overexpression of WT GFP-NEK1 in *NEK1* knockout U-2 OS cells (Ion Score 53.6; observed m/z 691.8231, $[M+2H]^{2+}$; Mascot site analysis: Phospho T7 100%; ptmRS: T7 100%).

Figure S9: Mascot-derived annotated peptide-spectrum match reports supporting phosphosite localisation for pSer14, pThr156 and pSer418 in wild-type GFP-NEK1—transient overexpression. Representative Mascot-derived annotated MS/MS spectra from transient overexpression of WT GFP-NEK1. (**A**) pSer14, identified by HCD fragmentation (Ion Score 48.2, observed m/z 437.6889, ptmRS: S5 100%). (**B**) pThr156, identified by HCD fragmentation (**Ion Score 63.2**, observed m/z 591.3002, Phospho T5 89.57%, ptmRS: T5 99.99%). (**C**) pThr156, identified by EThcD fragmentation (Ion Score 65.2, observed m/z **591.2996**, Phospho T5 95.32%, ptmRS: T5 99.96%). (**D**) pSer418, identified by HCD fragmentation (Ion Score 60.2, observed m/z 599.2790, Phospho S8 99.89%, ptmRS: S8 100%). Fragment ions are annotated with b-ions (red) and y-ions (orange); the observed spectrum is shown in black alongside the theoretical spectrum.

Figure S10: Mascot-derived annotated peptide-spectrum match reports supporting phosphosite localisation for pSer14, pThr156 and pSer418 in wild-type GFP-NEK1—stable expression. Representative Mascot-derived annotated MS/MS spectra from stable doxycycline-inducible expression of WT GFP-NEK1. (**A**) pSer14, identified by HCD fragmentation (Ion Score 32.4, observed m/z 437.6895, ptmRS: S5 100%). (**B**) pThr156, identified by HCD fragmentation (Ion Score 51.2, observed m/z 591.3008, Phospho T5 88.21%, ptmRS: T5 100%). (**C**) pThr156, identified by EThcD fragmentation (Ion Score 46.2, observed m/z 591.2993, Phospho T5 94.43%, ptmRS: T5 99.04%). (**D**) pSer418, identified by HCD fragmentation (Ion Score 43.4, observed m/z 599.2793, Phospho S8 98.64%, ptmRS: S8 100%). Fragment ions are annotated with b-ions (red) and y-ions (orange); the observed spectrum is shown in black alongside the theoretical spectrum.

Figure S11: Structural context and conservation of NEK1 autophosphorylation sites, and C21ORF2 binding by phosphosite mutants. (**A**) Predicted structural context of the three NEK1 autophosphorylation sites before and after phosphorylation. AlphaFold3 models of WT NEK1 (residues 1–1,286), with residues 1–431 displayed, showing Ser14, Thr156 and Ser418 in the unphosphorylated state (left) and following phosphorylation (right); in each, the kinase domain is shown at the top and the extended C-terminal coiled-coil below. Boxed regions are enlarged in the central column, unphosphorylated (left) and phosphorylated (right) for each site. Ser14 lies within the glycine-rich (G-) loop of the kinase N-lobe, a region that is disordered and therefore unresolved in the available crystal structure, so its side-chain environment can only be assessed from the model. In the unphosphorylated state, the Ser14 side chain is oriented inwards, towards the body of the N-lobe, whereas Thr156 and Ser418 are solvent-exposed; none of the three residues makes

a notable intramolecular contact. The inward orientation of Ser14 should, however, be interpreted with caution: the G-loop is highly mobile, so Ser14 may sample solvent-exposed positions or adopt alternative side-chain rotamers not captured by a single static model. Following phosphorylation, pSer14 is positioned to form an ionic interaction with the guanidinium group of Arg161 (dashed line), whereas pThr156 and pSer418 do not appear to engage in any intramolecular interaction and remain largely solvent-exposed. The engagement of pSer14 by Arg161 suggests that this phosphosite may be partially occluded once formed, which could limit the accessibility of the epitope to phosphospecific antibodies raised against pSer14. (**B**) Surface representation of the same region (residues 1–431) coloured by ConSurf conservation grade (1–9; variable to conserved) across mammalian NEK1 orthologues (left) and across the human NEK kinase family (right), with the N-terminus and the end of the displayed region (residue 431) indicated. All three sites are surface-accessible and highly conserved within the NEK1 lineage, but show more variable conservation across the broader NEK family. (**C**) Anti-GFP co-immunoprecipitation from *NEK1* knockout U-2 OS cells expressing WT, KD^{D146A}, or phosphosite-mutant (T156A, S418A, S14A) GFP-NEK1, immunoblotted for NEK1 and C21ORF2. C21ORF2 co-precipitates with NEK1 in all constructs, indicating that the phosphosite substitutions do not disrupt the NEK1–C21ORF2 interaction. A NEK1-dependent C21ORF2 mobility shift is present in WT and in the phosphosite-mutant constructs, but is absent in KD^{D146A}. Ponceau S staining confirms comparable protein loading.

Figure S12: Dot blot validation of phosphospecific antibodies against NEK1 pSer14, pThr156 and pSer418. (**A**) Dot blot analysis of affinity-purified sheep polyclonal antibodies against pThr156 (second bleed; upper left), pSer418 (third bleed; upper middle) and pSer14 (third bleed; upper right). Serial dilutions of phosphorylated peptide (1000, 500, 250, 100, 10, 0.5, and 0.1 ng/µl), and the corresponding non-phosphorylated peptide counterpart were spotted onto nitrocellulose and probed with each antibody. All three antibodies detect their cognate phosphopeptide in a concentration-dependent manner, with no signal against the non-phospho peptide, confirming phosphospecificity. The pThr156 antibody was additionally subjected to tandem depletion purification (second bleed, tandem; lower panel), in which serum was first passed over non-phosphopeptide resin to remove antibodies recognising the peptide backbone independently of phosphorylation. The tandem-depleted preparation detects the cognate phosphopeptide to 250 ng/µl, compared with 10 ng/µl for the single-step preparation; this reduction in titre is the expected cost of the depletion step and is acceptable at the working concentrations used. The upper and lower panels were acquired as separate experiments, with different exposure settings and slightly different dilution series, and should not be read as a matched side-by-side comparison of sensitivity. The tandem-depleted preparation was used for the

immunoblotting experiments reported here. Phosphorylated residues are indicated in red in the peptide sequences shown below each blot.

Figure S13: Activation-loop mutagenesis reveals coupling between the Ser155/Thr156 region and distal NEK1 autophosphorylation sites. (**A**) Colloidal Blue-stained SDS-PAGE gel following anti-GFP immunoprecipitation from *NEK1* knockout U-2 OS cells transiently overexpressing WT, S155A, T156A or S155A/T156A GFP-NEK1. Excised NEK1 bands were subjected to in-gel Trypsin/Lys-C digestion and LC-MS/MS analysis. (**B**) XIC peak-area quantification for pSer14 and pSer418 across the indicated GFP-NEK1 constructs, normalised to total ion current within each run. S155A has no appreciable effect on pSer14 or pSer418 relative to WT NEK1, supporting the conclusion that Ser155 is not responsible for the signal assigned to pThr156. The S155A/T156A double mutant causes marked loss of pSer14, an effect not recapitulated by either single mutant, consistent with coupling between pSer14 and the Ser155/Thr156 activation-loop region rather than a strict requirement for Thr156 phosphorylation alone. T156A increases pSer418 relative to WT NEK1, whereas the S155A/T156A double mutant reduces pSer418 relative to T156A, suggesting altered distribution of autophosphorylation within the mutant proteins.

Figure S14: Ancestral-state reconstruction of NEK1 residue 721 across vertebrate orthologues. A maximum-likelihood phylogeny was inferred from a MAFFT alignment of 26 vertebrate NEK1 protein sequences using IQ-TREE v3.0.1 via the CIPRES Science Gateway. ModelFinder selected Q.BIRD+I+G4 as the best-fit model according to BIC, and branch support was assessed using 1000 SH-aLRT and 1000 ultrafast bootstrap replicates. The tree was rooted using avian taxa as the outgroup. Human NEK1 isoform 3 residue 721, corresponding to UniProtKB Q96PY6-3 and alignment column 745, was analysed using PastML with MAP prediction under the F81 model. Tip boxes indicate observed residues; numbered internal nodes indicate highlighted ancestral-state reconstructions with marginal posterior probabilities, with node support shown as SH-aLRT/UFBoot. The branch on which arginine was inferred to arise is highlighted in magenta. Glutamine (Q) was inferred at the root/amniote, mammalian and primate ancestral nodes, whereas arginine (R) was inferred at the catarrhine ancestor, consistent with a catarrhine-lineage gain of R followed by a gibbon-specific W state. Topology is shown for clarity; branch lengths are not shown to scale. The human R721Q substitution is therefore consistent with reversion toward the inferred ancestral glutamine state.

Figure S15: Size-exclusion chromatography of NEK1 and the NEK1–C21ORF2 complex. (**A**) Coomassie-stained SDS-PAGE of purified NEK1 alone and NEK1 co-purified with C21ORF2, with the NEK1 and C21ORF2 bands indicated. (**B**) Size-exclusion chromatograms (Superose 6 Increase 10/300) of NEK1 alone (left) and the NEK1–C21ORF2 complex (right); the void volume for this column is ~8 ml, and the fractions selected for mass photometry (NEK1 alone, fraction B2, eluting at 8.4 ml; NEK1–

C21ORF2, fraction B9, eluting at 12.3 ml) are indicated. (**C**) SDS-PAGE and (**D**) anti-NEK1 and anti-C21ORF2 immunoblot analysis of the collected fractions, confirming co-elution of NEK1 and C21ORF2.

Figure S16: Mass photometry of NEK1 and the NEK1–C21ORF2 complex. Mass distributions measured by mass photometry and fitted with Gaussian functions. NEK1 alone (left) resolves as a predominant species of 108 ± 49 kDa (78% of events), consistent with a monomer; the NEK1–C21ORF2 complex (right) resolves as a major species of 442 ± 73 kDa (65% of events), consistent with a putative 2:4 NEK1–C21ORF2 assembly, together with minor populations of 136 ± 55 kDa (20%) and 748 ± 138 kDa (12%). We note that, in size-exclusion chromatography, NEK1 alone elutes far earlier than expected for a NEK1 monomer (Figure S15B), giving an apparent molecular weight well above the mass measured here. Because mass photometry reports mass independently of shape, this discrepancy is most parsimoniously explained by a non-globular, extended conformation and/or self-association of NEK1 in the absence of C21ORF2. For the complex, the two measurements are concordant: it elutes well within the fractionation range of the column, and its measured mass approximates that calculated for a 2:4 NEK1–C21ORF2 assembly, consistent with a more compact species.

Table S1: Cell-based phosphoproteomic map of wild-type NEK1. Phosphorylation sites identified by LC-MS/MS following anti-GFP immunoprecipitation of GFP-tagged WT NEK1 from *NEK1* knockout U-2 OS cells, expressed either by transient overexpression or stable doxycycline-inducible expression (Flp-In T-REx system), and analysed using HCD or EThcD fragmentation. All positional numbering is given in NEK1 isoform 3 coordinates (UniProtKB Q96PY6-3; 1,286 amino acids). Raw positions in the workbook are given in the coordinates of the GFP-NEK1 construct entry used for database searching (MRC_Database_1 entry DU58446: EGFP 1–239; Ser-Gly-Leu-Gly-Ser linker 240–244; NEK1 isoform 3 245–1530); subtract 244 to convert a raw construct position to NEK1 isoform 3 coordinates. Localisation thresholds, per-column definitions and colour-coding are detailed in the workbook's Methods & Notes sheet; in brief, sites pass where ptmRS ≥85% (or Mascot PTM confidence ≥85% where ptmRS was not computed), with a Mascot ion score >19 required throughout. Where the same peptide backbone appeared with alternative phosphorylated residues, only the highest-confidence localisation is retained per dataset; ambiguous cases are flagged in the Localisation Note column with alternative residue(s) indicated. Phosphosites detected exclusively via multiply-phosphorylated peptides were excluded where the co-phosphorylated residue on the same peptide was independently localised by a singly-phosphorylated species; this avoids double-counting of localisation confidence that cannot be independently attributed to each residue. Sheet guide — OEx HCD WT: all phosphosites detected in the transient overexpression HCD dataset. Stable HCD WT: all phosphosites detected in the stable expression HCD dataset. OEx EThcD WT: all phosphosites detected in the transient overexpression EThcD dataset.

Stable EThcD WT: all phosphosites detected in the stable expression EThcD dataset. Common All 4 Datasets: phosphosites detected reproducibly across all four acquisition conditions, with Mascot PTM confidence and ptmRS scores from each dataset shown separately; ten sites met this criterion. PhosphoSitePlus® (NEK1 iso3): all phosphorylation sites currently annotated for human NEK1 isoform 3 in PhosphoSitePlus® [32], with indication of whether each site was detected in the present experimental data. Common All 5 (incl. PSP): Phosphosites common to all four experimental datasets and corroborated by PhosphoSitePlus®. Methods & Notes: data processing rules applied throughout, including the multi-phospho peptide curation rule and threshold logic. Summary: overview of the number of phosphosites identified per dataset. Sequences: the GFP-NEK1 construct and NEK1 isoform 3 sequences used for database searching and residue numbering. ✓, detected; ✗, not detected; —, localisation ambiguous or site not in PhosphoSitePlus®. Colour coding (per row): green = detected in both this study and PhosphoSitePlus®; yellow = detected in this study, not in PhosphoSitePlus®; blue = in PhosphoSitePlus® but not detected; white = not applicable. pThr1096 did not meet the strict four-dataset reproducibility criterion (absent from Stable EThcD) and is retained as a provisional candidate.

Table S2: Cell-based phosphoproteomic map of kinase-dead NEK1. Phosphosites detected in KD^{D146A} GFP-NEK1 across transient overexpression and stable doxycycline-inducible expression datasets, analysed using HCD and EThcD fragmentation. Comparison with the wild-type cell-based phosphosite map in Table S1 and the summary matrix in Figure S5A identifies recurrent sites absent from KD^{D146A} cells and sites still detected in KD^{D146A} cells that required recombinant WT/KD^{D146A} mapping and targeted XIC analysis for further assessment. All positional numbering is given in NEK1 isoform 3 coordinates (UniProtKB Q96PY6-3; 1,286 amino acids). Raw positions in the workbook are given in the coordinates of the kinase-dead GFP-NEK1 construct entry used for database searching (MRC_Database_1 entry DU80306, GFP-NEK1 D146A: EGFP 1–239; Ser-Gly-Leu-Gly-Ser linker 240–244; NEK1 isoform 3 245–1530), which shares its numbering with the wild-type entry DU58446; subtract 244 to convert a raw construct position to NEK1 isoform 3 coordinates. The D146A substitution abolishes catalytic activity by disrupting the conserved DFG motif required for Mg^{2+}-dependent ATP positioning and phosphotransfer; phosphorylation sites detected under this condition therefore represent constitutive or trans-phosphorylation events that do not require NEK1 catalytic activity. Column structure, thresholds and curation rules are as for electronic supplementary material, Table S1 (see its Methods & Notes sheet). Phosphosites detected exclusively via multiply-phosphorylated peptides were excluded where the co-phosphorylated residue was independently localised by a singly-phosphorylated species. Sheet guide — Stable HCD KD: stable expression HCD KD^{D146A} dataset. Stable EThcD KD: stable expression EThcD KD^{D146A} dataset. OEx HCD KD: transient overexpression HCD KD^{D146A} dataset. OEx

EThcD KD: transient overexpression EThcD KD^{D146A} dataset. Legend: colour key and threshold notes. Summary: number of sites detected per dataset. Colour coding — green: site also detected in the wild-type dataset (electronic supplementary material, Table S1) and annotated in PhosphoSitePlus®; yellow: in WT dataset, but not in PhosphoSitePlus®; blue: in PhosphoSitePlus® but not detected in WT; white: detected only in the KD^{D146A} condition. Sites detected in both WT and KD^{D146A} conditions should not be classified as NEK1 autophosphorylation sites on the basis of binary detection alone. Conversely, sites detected in WT, but not KD^{D146A} cells, represent candidate kinase-activity-associated phosphosites that require quantitative XIC analysis and/or orthogonal biochemical validation for autophosphorylation-site assignment. Autophosphorylation-site assignment in the main text is based on the combined evidence from electronic supplementary material, Tables S1 and S2, recombinant WT/ KD^{D146A} mapping, XIC quantification where performed (Figure 3B; source data in electronic supplementary material, Table S4), phosphosite mutagenesis and phosphospecific antibody validation.

Table S3: Recombinant NEK1 phosphoproteomic map (wild-type and kinase-dead). Phosphorylation sites identified by LC-MS/MS on recombinant full-length NEK1 that was expressed in Sf9 insect cells as a GFP-tagged construct, co-expressed with C21ORF2, purified by anti-GFP affinity chromatography, and analysed after RV-3C tag cleavage, comparing WT NEK1 with KD^{D146A} NEK1. Each condition was analysed in three independent biological replicates using both HCD and EThcD fragmentation. A stringent 3/3 replicate threshold was applied: a site is included only where it was detected in all three replicates of the relevant condition. All positional numbering is given in NEK1 isoform 3 coordinates (UniProtKB Q96PY6-3; 1,286 amino acids). Raw positions in the workbook are given in the coordinates of the GFP-NEK1 entries of MRC_Database_1 against which the spectra were searched (DU58446 wild-type and DU80306 D146A: EGFP 1–239; Ser-Gly-Leu-Gly-Ser linker 240–244; NEK1 isoform 3 245–1530; both share this numbering); subtract 244 to convert a raw construct position to NEK1 isoform 3 coordinates. The recombinant protein analysed here was expressed from the baculovirus transfer vectors listed in electronic supplementary material, Table S5, whose tag architecture differs; the raw positions therefore refer to the search-database numbering rather than to that of the expression construct. Column structure and threshold logic are as described for electronic supplementary material, Tables S1 and S2. Phosphosites detected exclusively via multiply-phosphorylated peptides were excluded where the co-phosphorylated residue was independently localised by a singly-phosphorylated species (multi-phospho peptide curation rule; details in the Key Notes section of the Legend sheet). The 3/3* notation indicates detection in all three replicates by any phospho form (singly or multiply phosphorylated) but in fewer than three replicates by singly-phosphorylated peptide alone. Sheet guide — WT HCD: phosphosites detected 3/3 in WT HCD replicates. WT EThcD: phosphosites detected 3/3 in WT EThcD replicates. D146A HCD: phosphosites detected

3/3 in KD^{D146A} HCD replicates. D146A EThcD: phosphosites detected 3/3 in KD^{D146A} EThcD replicates. Common All 4: phosphosites detected 3/3 across all four acquisition conditions. Legend: colour coding, column guide, sample key and data-processing notes, including curation decisions. Summary: number of curated sites per dataset, and the prioritised candidate sites absent from the kinase-dead datasets. Sites absent from KD^{D146A} datasets, but present in WT datasets, are candidate NEK1 autophosphorylation sites in the recombinant system; sites present in both conditions are constitutive or trans-phosphorylation events acquired from Sf9 insect cell kinases. Comparison with the cell-based WT and KD^{D146A} maps in electronic supplementary material, Tables S1 and S2, together with XIC quantification where performed (Figure 3B; source data in electronic supplementary material, Table S4), provides orthogonal evidence for autophosphorylation-site assignment in the main text.

Table S4: Source data for XIC quantification of NEK1 phosphosites (Figure 3B). Wild-type *versus* kinase-dead (WT/KD^{D146A}) peak-area quantification for the candidate phosphosites assessed by XIC. Raw XIC Data: integrated peak areas for each phosphopeptide across WT and KD^{D146A} conditions and acquisition methods. Calculations: per-site WT/ KD^{D146A} peak-area ratios across conditions, with minimum and maximum fold-changes. Figure Data (log2): log2-transformed WT/ KD^{D146A} ratios used to generate Figure 3B. Methods Note: data-processing and normalisation details (peak areas normalised to total ion current).

Table S5: Key resources and reagents. Plasmids, antibodies, cell lines and oligonucleotides generated or used in this study, provided as a multi-sheet workbook. Sheet guide — Plasmids – Mammalian: expression constructs for mammalian cell work; Plasmids – CRISPR KO: guide RNA and targeting constructs for *NEK1* knockout generation; Plasmids – Recombinant: constructs for recombinant protein expression in insect cells; Plasmids – Stable Lines: constructs for Flp-In T-REx stable cell line generation; Antibodies: primary and secondary antibodies, including in-house phosphospecific sheep polyclonal antibodies (anti-pSer14, anti-pThr156, anti-pSer418) with antigen sequences and identifiers; Cell Lines: parental and engineered cell lines with sources; Oligonucleotides: primers and guide sequences. Catalogue numbers, internal identifiers, and source details are provided per item where applicable.

Table S6: Single-variant and gene-burden association statistics for *NEK1* in ALS. Association results for *NEK1* variants from a large-scale exome-wide rare variant study comprising 13,138 ALS cases and 69,775 controls [11]. Variants were tested using Firth logistic regression with the Rare Variant Association Toolkit (RVAT; [78]), with correction for sex, genome-wide burden of synonymous variants, and population structure; a nominal significance threshold of $P < 0.05$ was applied. Variant nomenclature follows the MANE Select transcript (NM_001199397.3 / ENST00000507142.6), encoding NEK1 isoform 3

(1,286 amino acids). **Sheet 1 (NEK1_sv_full) — single-variant association.** Columns: CHROM, POS (GRCh38 genomic coordinates); ID (dbSNP rsID; "." if none); REF/ALT (reference and alternate alleles); HGVSc, HGVSp (cDNA- and protein-level nomenclature); domain (annotated protein domain or region); pass_callrate (TRUE/FALSE; see below); caseMAF, ctrlMAF (minor allele frequency in cases and controls); OR, ORCIlower, ORCIupper (odds ratio and 95% confidence interval); *P* (association *P* value). Variants are ordered by ascending *P*. Rows with blank *P*, OR and MAF fields correspond to variants that were observed but lacked a computable association statistic, because the variant had too few observations (a single observation) to support a valid statistical test. The pass_callrate column indicates whether a variant passes the stringent call-rate quality-control filters used in the exome-wide discovery analysis (filtering to TRUE reproduces the discovery variant set). Variants failing this filter (FALSE) are retained because these thresholds, whilst appropriate for unbiased exome-wide discovery, exclude some established ALS-associated variants; for targeted interrogation of a known ALS gene such as *NEK1* these remain informative, consistent with the ALS Gene Curation Expert Panel (GCEP) framework applied in the original study. **Sheet 2 (NEK1_burden) — gene-burden association.** Burden test results (Firth logistic regression) for four variant sets (MAF < 0.05): missense; missense excluding the recurrent p.Arg261His variant; missense + loss-of-function (LOF); and missense + LOF excluding p.Arg261His. Columns: varSetName, bin; nvar; meanCaseScore, meanCtrlScore; OR, ORCIlower, ORCIupper; *P*. Exclusion of p.Arg261His markedly attenuates the missense-only signal but the missense + LOF burden remains significant, indicating contributions from both the recurrent variant and additional rare deleterious alleles.
